# FcγR- and CD9-dependent synapse-engulfing microglia in the thalamus drive cognitive impairment following cortical brain damage in mice

**DOI:** 10.1038/s41467-026-74904-1

**Published:** 2026-07-09

**Authors:** Ken Matoba, Takahiro Kochi, Oluwaseun Fatoba, Md Sorwer Alam Parvez, Inssaf Berkiks, Yassin R. Mreyoud, Harrison Strong, Jana H. Badrani, Hency Patel, Yoshiko Nagaoka-Kamata, Masakazu Kamata, Hiroshi Tsujioka, Toshihide Yamashita, David K. Crossman, Minae Niwa, Shin-ichi Kano

**Affiliations:** 1 Department of Psychiatry and Behavioral Neurobiology, University of Alabama at Birmingham Heersink School of Medicine, Birmingham, AL USA; 2 Department of Mental Disorder Research, National Institute of Neuroscience, National Center of Neurology and Psychiatry, Tokyo, Japan; 3 Department of Pathology, University of Alabama at Birmingham Heersink School of Medicine, Birmingham, AL USA; 4 Department of Microbiology, University of Alabama at Birmingham Heersink School of Medicine, Birmingham, AL USA; 5 O’Neal Comprehensive Cancer Center, University of Alabama at Birmingham Heersink School of Medicine, Birmingham, AL USA; 6 Department of Molecular Neuroscience, Graduate School of Medicine, The University of Osaka, Osaka, Japan; 7 Department of Genetics, University of Alabama at Birmingham Heersink School of Medicine, Birmingham, AL USA; 8 Department of Neurobiology, University of Alabama at Birmingham Heersink School of Medicine, Birmingham, AL USA; 9 Department of Biomedical Engineering, University of Alabama at Birmingham School of Engineering, Birmingham, AL USA

**Keywords:** Neuroimmunology, Neuroimmunology

## Abstract

Chronic neuroinflammation gives rise to diverse microglial states across the brain, yet how region-specific microglial remodeling contributes to cognitive dysfunction remains unclear. Here we report that synapse-engulfing microglia in the thalamus drive cognitive impairment after cortical brain damage in mice, primarily studied in females. Region-specific manipulations of microglia during the chronic phase show that reactive microglial changes in the thalamus, but not in the hippocampus, impair recognition memory. Single-cell RNA sequencing reveals an enrichment of synapse-engulfing CD9^hi^ microglia in the thalamus. Antibody-based CD9 blockade in the thalamus, as well as microglia-selective CD9 disruption, rescues thalamic synaptic loss, restores neuronal activity, and improves recognition memory. Further analysis shows that the blood-brain barrier disruption and subsequent γ-immunoglobulin (IgG) extravasation facilitate the generation of CD9^hi^ microglia in an Fcγ receptor III-dependent manner. These findings demonstrate that the induction of synapse-engulfing CD9^hi^ microglia in the thalamus by IgG/FcγRIII signaling drives recognition memory deficits following cortical damage.

## Abstract (web summary)

Cortical brain injury leads to chronic neuroinflammation and cognitive deficits. Here, the authors show in a mouse model of cortical damage that IgG/FcγR-driven induction of CD9hi microglia in the thalamus causes post-injury memory impairment via synapse removal.

## Introduction

Microglia are resident immune cells in the central nervous system and play both neuroprotective and neurotoxic roles in neuroinflammation^1–4^. Microglia respond to infection and tissue injury by recognizing danger molecules, secreting inflammatory mediators, performing phagocytosis, and facilitating the infiltration of other immune cells. As they transition into reactive states, microglia also lose their homeostatic function to support neurons, dysregulating neuronal activities and damaging neurons. Recent single-cell transcriptomic studies have revealed diverse states of reactive microglia across neurodegenerative conditions, such as disease-associated microglia (DAM)—first described in mouse models carrying Alzheimer’s disease-related mutations^5–8^. Reactive microglial changes are also heterogeneous across different brain regions^9–11^. However, the impact of various microglial states on neuronal function and cognitive outcomes is not fully understood.

Traumatic brain injury (TBI) causes a long-lasting impact on the brain, inducing chronic neuroinflammation and impairing cognitive function, with increased risks of developing dementia^12–22^. Reactive microglial changes occur rapidly in the primary injury area and later appear in multiple brain regions, such as the hippocampus, thalamus, and corpus callosum, as part of secondary injury^20,23–26^. Similar to other chronic neuroinflammatory conditions, microglial states after brain injuries are highly heterogeneous, both temporally and regionally^25,27^. These reactive microglia secrete pro-inflammatory cytokines, phagocytose cellular debris and synapses, promote immune cell infiltration, and facilitate brain repair processes^26,28,29^. Several studies using mouse models of cortical brain injuries have reported that microglia depletion mitigates post-injury cognitive impairment, underscoring their causal involvement in persistent neuronal dysfunction^30–32^. Nevertheless, single-cell-level analyses of microglial diversity in the injured brain remain limited compared with studies in neurodegenerative diseases, such as Alzheimer’s disease^33–37^. Consequently, the mechanistic links between distinct microglial states and neuronal dysfunction, particularly those in secondary injury, remain poorly defined. Elucidating such relationships will be critical for understanding the long-lasting effects of cortical damage on cognition and for identifying microglia-targeted therapeutic strategies capable of modifying disease trajectory.

In this study, we combine the region-specific and microglia-selective manipulations to define how reactive microglia contribute to neuronal dysfunction and cognitive impairment in the chronic phase after cortical damage, using female mice with additional analyses in males. We determine reactive microglial changes in the thalamus as primary drivers of recognition memory deficits, and identify CD9^hi^ microglia, defined by single-cell RNA sequencing and flow cytometry, that mediate synapse loss, neuronal dysfunction, and recognition memory deficits. Finally, we demonstrate that the blood-brain barrier (BBB) dysfunction and subsequent IgG extravasation in the thalamus promote the generation of CD9^hi^ microglia via Fcγ receptor III, revealing a mechanistic link between vascular damage and pathogenic microglial states.

## Results

### Cortical damage induces delayed thalamic microglia changes that correlate with recognition memory deficits in mice

**Fig. 1:**
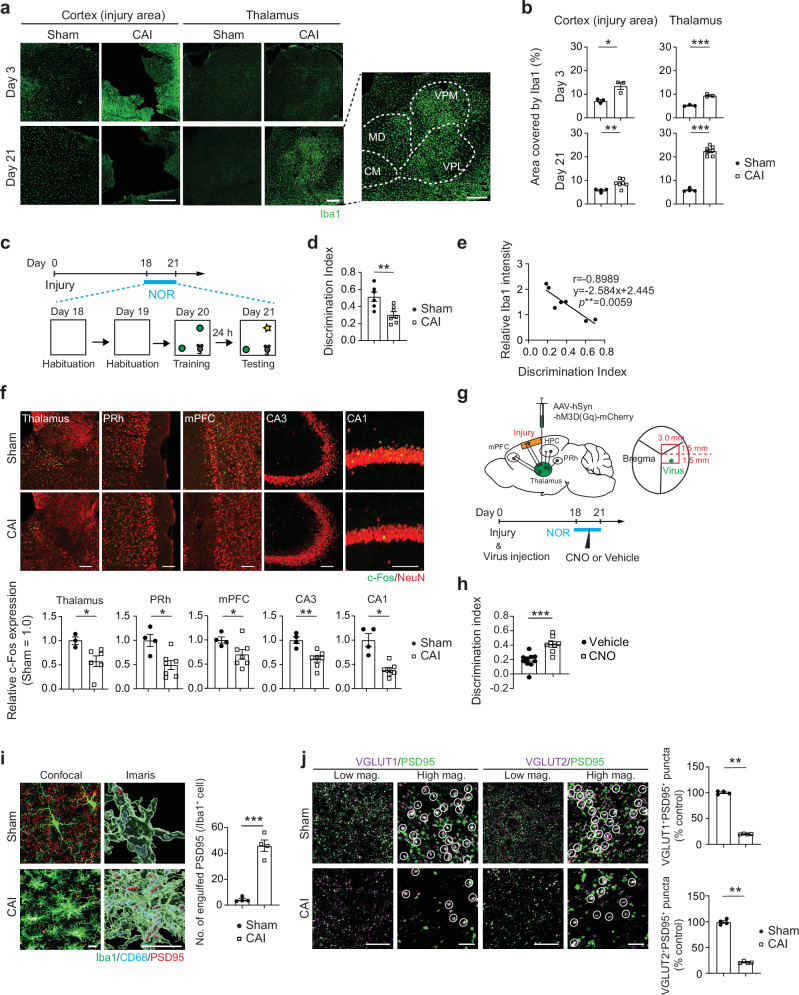
Cortical brain damage induces reactive changes in thalamic microglia, reduces thalamic neuronal synapses, and impairs recognition memory. **a** Representative images of Iba1^+^ cells in the cortex (primary lesion) and the thalamus, 3 and 21 days after unilateral cortical ablation injury (CAI) and sham surgery. MD mediodorsal nucleus, CM central medial nucleus, VPL ventral posterolateral nucleus, VPM ventral posteromedial nucleus. Scale bars, 500 µm. **b** Quantification of the area covered by Iba1^+^ cells (%) in the cortex and thalamus (day 3, *n* = 3 mice for sham and *n* = 3 mice for CAI; day 21, *n* = 4 for sham and *n* = 7 for CAI). **c** Experimental timeline for novel object recognition (NOR) test after CAI. **d** Discrimination index in the NOR test (*n* = 6 mice for sham; *n* = 7 mice for CAI). The discrimination index was calculated as ([time spent at the novel object] − [time spent at the familiar object])/([time spent at the novel object] + [time spent at the familiar object]). **e** Correlation plot of the discrimination index and thalamic Iba1 intensity 21 days after CAI and sham surgery (*n* = 7 mice). Pearson’s correlation coefficient (*r*) and two-tailed *p* values are shown in the plot. **f** Representative images (left) and quantification (right) of neuronal c-Fos expression in the thalamus, PRh, mPFC, and HPC (CA1, CA3) 21 days after CAI and sham surgery. Scale bars, 100 µm. Quantification data show neuronal c-Fos expression in the CAI group (thalamus, *n* = 3 mice; other regions, *n* = 7 mice) relative to the sham group (thalamus, *n* = 3 mice; other regions, *n* = 4 mice). **g** Experimental scheme and timeline for neuronal DREADD rescue experiment. AAV-hSyn-hM3D(Gq)-mCherry was injected into the thalamus on the day of CAI, and CNO or vehicle was administered intraperitoneally before the test session in the NOR test. **h** Discrimination index in the NOR test for vehicle-treated (*n* = 10 mice) and CNO-treated mice (*n* = 8 mice). **i** Representative confocal and Imaris-reconstructed 3D images (left) and quantification (right) of PSD95 engulfment by Iba1^+^ cells in the thalamus 21 days after CAI or sham surgery. Scale bars, 10 μm. The number of engulfed PSD95 puncta inside CD68^+^ lysosomes is quantified per cell. (*n* = 4 mice per group). **j** Representative images (left) and quantification (right) of VGLUT1/PSD95 and VGLUT2/PSD95 synapses in the thalamus 21 days after CAI or sham surgery. Scale bars, 10 μm (low magnification; Low mag.) and 2 μm (high magnification; High mag.). Quantification data show the synapse density in the thalamus of the CAI group relative to that of the sham group (*n* = 4 mice per group). In bar graphs, data are shown as the mean ± s.e.m, and each dot represents an individual animal. n indicates individual mice. For **d**, **f**, **h**, data were collected from 3 independent cohorts and pooled for analysis. For the others, data were collected from a single cohort with multiple mice. *p < 0.05, **p < 0.01, and ***p < 0.001; n.s. not significant (unpaired two-tailed t-test). Further details of the statistical analysis are available in the Supplementary Data S8. Source data are provided as a Source data file.

To identify the reactive microglial changes most critical for cognitive impairment after cortical damage, we first systematically assessed microglial changes across various brain regions using cortical ablation injury (CAI)^11,38,39^, targeting the primary sensory and motor cortex. In the CAI model, the cortical tissues were surgical resected to recapitulate cortical tissue loss by traumatic brain injuries (Supplementary Fig. 1a). Increased levels of Iba1 protein expression, an indicator of reactive microglial changes, were observed in the primary cortical lesion area immediately after injury and attenuated by 21 days post-injury (Fig. 1a, b). In contrast, Iba1 expression in other brain regions began to increase on day 7 and later. By day 21, the thalamus stood out as one of the regions with the most remarkable Iba1 expression (Fig. 1a, b, and Supplementary Fig. 1b–d). The increased Iba1 expression was also associated with microglial morphological changes of rounder somas with shortened processes (Supplementary Fig. 1b). In the thalamus, microglial changes began in the ventrolateral nucleus (VL) around day 7 and spread to the ventral posterolateral nucleus (VPL), anterodorsal nucleus (AD), anteroventral nucleus (AV), centromedian nucleus (CM), and mediodorsal nucleus (MD) by day 21 (Supplementary Fig. 1d). In addition to the drastic morphological changes, thalamic microglia expressed high levels of a pro-inflammatory cytokine, tumor necrosis factor-α (TNF-α) and a lysosome-associated protein, CD68 (Supplementary Fig. 1e, f). We also observed similar microglial reactive changes in the open controlled cortical impact (CCI) and repeated closed CCI, two representative mouse TBI models (Supplementary Fig. 1g–i). For the rest of the studies, we primarily used CAI unless otherwise described.

Cortical damage led to deficits in the novel object recognition (NOR) test and the Y-maze test on day 21 (Fig. 1c, d, and Supplementary Fig. 2a). Notably, a strong correlation was observed between NOR deficits (measured by discrimination index) and Iba1 staining intensity, particularly in the thalamus (Fig. 1e and Supplementary Fig. 2b). The data is reminiscent of previous findings in human PET imaging studies where inflammatory signals related to microglia in the thalamus were correlated with cognitive impairment in TBI patients in a chronic phase of injuries^20^. We primarily used female mice in this study because previous human studies indicated worse outcomes in females with traumatic brain injuries^40–42^. Nevertheless, reactive microglial changes in the thalamus and NOR deficits after cortical injuries were similarly observed in male mice (Supplementary Fig. 2c, d). Collectively, our data indicate that thalamic microglia changes may be a critical step leading to cognitive impairment after cortical brain damage.

### Thalamic neuronal dysfunction is causal for NOR deficits after cortical damage

Reduced expression of neuronal c-Fos proteins, an indicator of neuronal activity, was observed in the thalamus of injured mice after the NOR test (Fig. 1f), indicating an impaired activation of thalamic neurons. The NOR behavior is regulated by a network of neurons in multiple brain regions, including the perirhinal cortex (PRh), medial prefrontal cortex (mPFC), and hippocampus (HPC), in addition to the thalamus^43,44^. Notably, reduced c-Fos expression was also observed in the PRh, mPFC, and HPC (Fig. 1f). These results suggested that thalamic neuronal dysfunction could cause recognition memory deficits by negatively impacting the other neuronal activities linked to NOR. To address this possibility, we locally enhanced thalamic neuronal activities using a chemogenetic approach with the designer receptors exclusively activated by designer drugs (DREADD) in the injured mice (Fig. 1g)^45^. Adeno-associated virus vectors expressing an activator DREADD under a neuron-specific promoter [AAV-hSyn-hM3D(Gq)-mCherry] were stereotactically injected into the thalamus of cortically injured mice (Supplementary Fig. 2e). Then, clozapine *N*-oxide (CNO) was administered intraperitoneally to activate DREADD. As expected, neuronal c-Fos expression increased in the thalamus of injured mice upon CNO administration (Supplementary Fig. 2f). Neuronal c-Fos expression also increased in the PRh, mPFC, and HPC, supporting our hypothesis that reduced thalamic neuronal activities impact neuronal activities in these brain regions. Under this condition, the CNO-treated injured mice showed an improved NOR (Fig. 1h). CNO injection did not affect microglial reactive changes, neuronal c-Fos expression, or NOR in the injured mice (Supplementary Fig. 2g–j). CNO injection with AAV-hSyn-hM3D(Gq) also enhanced NOR in the uninjured mice, while CNO injection with control AAV [AAV-hSyn-mCherry] did not affect NOR in the injured mice (Supplementary Fig. 2k, l). Thus, our data demonstrate that the reduced neuronal activities in the thalamus is causal for NOR deficits after cortical damage.

### Cortical damage induces pathological microglial engulfment of glutamatergic synapses, leading to synapse loss in the thalamus

Synaptic and neuronal loss can cause NOR deficits following cortical damage. Indeed, we observed that a significant population of thalamic microglia in the injured mice contained PSD95, a postsynaptic scaffold protein, within their enlarged CD68^+^ lysosomes on day 21, suggesting microglial phagocytosis of synapses (Fig. 1i). Consistent with these data, the overall number of glutamatergic synapses (both VGLUT1/PSD95 and VGLUT2/PSD95 synapses) in the thalamus significantly decreased in the injured mice compared to the sham mice (Fig. 1j). We also observed a significant loss of neurons in the thalamus although thalamic microglia did not seem to directly phagocytose neuronal perikaryal (Supplementary Fig. 3a, b). In contrast, synaptic and neuronal loss were absent in other regions, such as the mPFC, PRh, and HPC, although we observed mild reactive changes in microglia in the HPC (Supplementary Fig. 3c, d). Reactive microglial changes and synaptic/neuronal loss in the thalamus were detected even 35 and 63 days after cortical damage (Supplementary Figs. 1c and 3e, f). These findings demonstrate that reactive microglial changes lead to long-lasting loss of glutamatergic synapses and neurons in the thalamus of injured mice.

We also conducted single-nucleus RNA-seq (snRNA-seq) to further determine the changes in thalamic neurons at the molecular level. We identified 16 neuronal subclusters; Three clusters (clusters 1-3) belong to Gad1^+^ GABAergic inhibitory neurons, and the other 13 clusters (clusters 4–16) belong to Slc17a6^+^ glutamatergic excitatory neurons (Supplementary Fig. 4a). Cortical brain damage altered the gene expression patterns in neuronal clusters, particularly glutamatergic #3 (Supplementary Fig. 4b, c). Specifically, glutamatergic neurons (#3, #1, and #2) show a significant reduction in genes related to protein translation at synapses and in the cytoplasm, possibly indicating high cellular stress. Consistent with these data, we observed a significant decrease in thalamic excitatory neuron termini (VGLUT2^+^ puncta), but no change in thalamic GABAergic neuron termini (GAD67^+^ puncta) (Supplementary Fig. 4d). Our data suggest that thalamic reactive microglial changes following cortical damage negatively impact mostly glutamatergic neurons.

### Microglial reactive changes in the thalamus are necessary and sufficient for neuronal dysfunction and recognition memory deficits

**Fig. 2:**
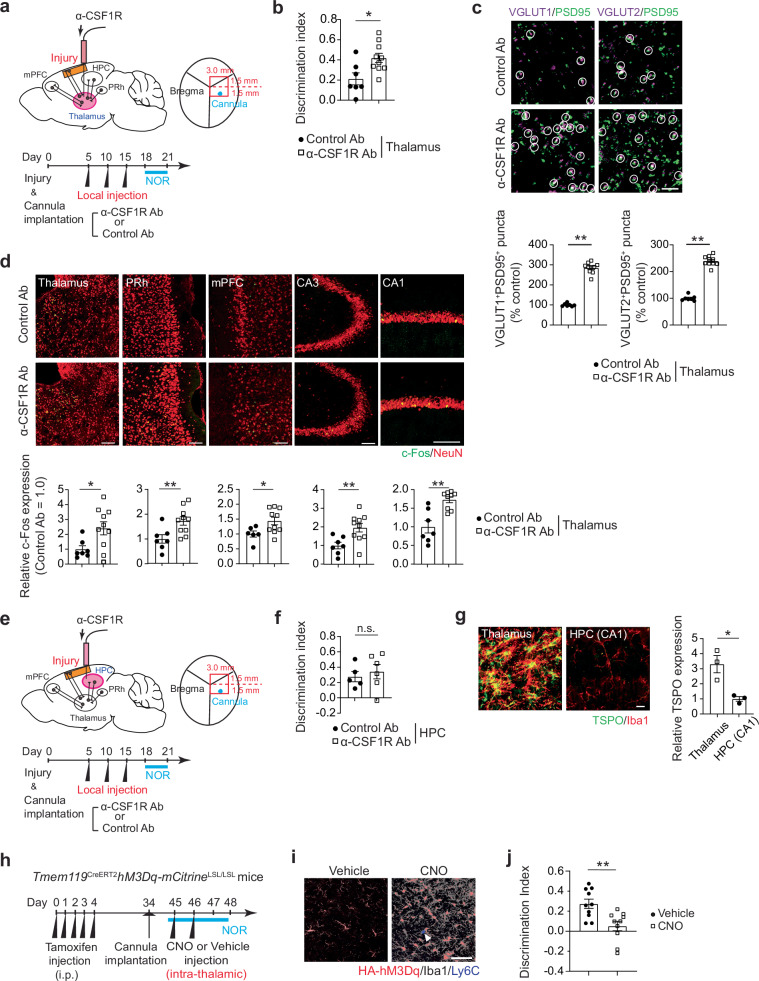
Reactive changes in thalamic microglia are necessary and sufficient to impair neuronal function and recognition memory. **a** Experimental scheme and timeline for local depletion of thalamic Iba1^+^ cells by anti-CSF1R Ab injection. **b** Discrimination index in the NOR test for the control Ab group (*n* = 7 mice) and the anti-CSF1R Ab group (*n* = 10 mice) after CAI. **c** Representative images (left) and quantification (right) of VGLUT1/PSD95 and VGLUT2/PSD95 synapses in the thalamus 21 days after CAI in the control Ab-injected and anti‑CSF1R Ab-injected group. Scale bar, 2 μm. Quantification data show the synapse density in the thalamus of the anti‑CSF1R Ab-injected group (*n* = 10 mice) relative to that of the control Ab-injected group (*n* = 7 mice). **d** Representative images (top) and quantification (bottom) of neuronal c-Fos expression in the thalamus, PRh, mPFC, and hippocampus (CA1, CA3) 21 days after CAI in the control Ab-injected and anti-CSF1R Ab-injected group. Scale bars, 100 µm. Quantification data show neuronal c-Fos expression in the anti-CSF1R Ab-injected group (*n* = 10 mice) relative to the control Ab-injected group (mPFC, *n* = 6 mice; other regions, *n* = 7 mice). **e** Experimental strategy for local depletion of hippocampal Iba1^+^ cells by anti-CSF1R Ab injection. **f** Discrimination index in the NOR test for the mice with intrahippocampal injections of control Ab (*n* = 5 mice) and anti-CSF1R Ab (*n* = 6 mice). **g** Representative images (left) and quantification (right) of TSPO expression in Iba1^+^ cells of the thalamus and hippocampus (HPC) (CA1) 21 days after CAI. Scale bar, 10 µm. Quantification data show relative TSPO expression in Iba1^+^ cells in the thalamus relative to the HPC (*n* = 3 mice per region). **h** Experimental timeline for DREADD experiments. *Tmem119*^*CreERT2*^*hM3Dq-mCitrine*^*LSL/LSL*^ mice received intraperitoneal (i.p.) injections of tamoxifen for 5 consecutive days to induce hM3D(Gq)-mCitrine expression in Tmem119^+^ microglia. Then, the mice were implanted with cannulas into the thalamus. Two weeks after the cannula implantation, the mice underwent the NOR test. CNO or vehicle was injected via implanted cannula 30 min before the habituation and training sessions of the NOR test. **i** Representative images of HA-tagged hM3D(Gq) DREADD expression in Iba1^+^ Ly6C^−^ microglia or Iba1^+^ Ly6C^+^ infiltrating macrophages in the thalamus in *Tmem119*^*CreERT2*^*hM3Dq-mCitrine*^*LSL/LSL*^ mice after tamoxifen injections and CNO or vehicle injection. Images are representative of 3 mice per group. An arrowhead indicates Iba1^+^ Ly6C^+^ cells. Scale bar, 10 µm. **j** Discrimination index in the NOR test for the vehicle- and CNO-injected group (*n* = 10 mice per group). In bar graphs, data are shown as the mean ± s.e.m, and each dot represents an individual animal. *n* indicates individual mice. For **b**–**d**, **j**, data were collected from 3 independent cohorts and pooled for analysis. For **f**, data were collected from 2 independent cohorts and pooled for analysis. For the others, data were collected from a single cohort. *p < 0.05, **p < 0.01; n.s. not significant (unpaired two-tailed t-test). Further details of the statistical analysis are available in the Supplementary Data S8. Source data are provided as a Source data file.

To address the role of thalamic microglial reactive changes in neuronal dysfunction and cognitive impairment, we locally injected anti-colony stimulating factor 1 receptor antibodies (anti-CSF1R Ab), which have been used to deplete macrophages and microglia^46–49^, into the thalamus of injured mice via implanted cannulas (Fig. 2a). The antibody was restricted to the thalamus at 1 and 6 h after injection and diminished by 24 h (Supplementary Fig. 5a), validating our local injection method as region-selective manipulation strategy. Repeated injections in cortically injured mice reduced the number of reactive microglia in the thalamus (Supplementary Fig. 5b). Flow cytometry analysis also confirmed the reduction of CD45^+^CD11b^+^ cells in the thalamus, but not in the peripheral blood after cortical damage (Supplementary Fig. 5c–e). Although the infiltration of Ly6C^+^ macrophages was detected in the thalamus, Ly6C^−^ microglia occupied the majority (>90%) of CD45^+^CD11b^+^ cells in the thalamus and were significantly reduced by anti-CSF1R Ab injections (Supplementary Fig. 5d). Other potential targets of anti-CSF1R Ab, such as border-associated macrophages and choroid plexus macrophages, were also evaluated but sparse in the thalamic inflammatory area (Supplementary Fig. 5f). These results validate that local injections of anti-CSF1R Ab primarily reduce microglia in the thalamus of injured mice.

Anti-CSF1R Ab injections into the injured mice also attenuated their NOR and Y-maze deficits, restored neuronal c-Fos expression in the thalamus and other areas related to NOR, and reduced neuronal and synapse loss in the thalamus (Fig. 2b–d and Supplementary Fig. 5g, h). Anti-CSF1R Ab injection similarly restored NOR in the mice after the open CCI (Supplementary Fig. 5i, j). Hippocampal (HPC) microglia have been widely investigated in cognitive impairment after cortical brain injuries^30,31^. Unexpectedly, local injection of anti-CSF1R Ab into the HPC of injured mice did not restore NOR, despite the reduction of Iba1^+^ cells (Fig. 2e, f, and Supplementary Fig. 5k). Indeed, microglial changes in the HPC of injured mice were more subtle than those in the thalamus, with little expression of translocator protein (TSPO), a mitochondrial activation marker and an established target of microglia tracer in human PET imaging^20,50–52^ (Fig. 2g). Neuronal loss was also not evident in the HPC (Supplementary Fig. 3d). Together, these data show that reactive microglial changes after cortical damage vary across brain regions, and thalamic microglial changes are primarily responsible for NOR deficits.

We next tested whether reactive changes in thalamic microglia were sufficient to impair NOR in uninjured mice. We first injected lipopolysaccharides (LPS) locally into the thalamus of non-injured mice via cannulas to induce microglial reactive changes (Supplementary Fig. 6a). LPS injection resulted in remarkable thalamic microglial reactive changes and NOR deficits, accompanied by reduced neuronal c-Fos expression in the thalamus and other NOR-related brain regions (Supplementary Fig. 6b–d). We also used a recently reported method of chemogenetic activation of microglia with an activator DREADD, hM3Dq^53^. We generated a conditional transgenic mouse line in which hM3Dq was expressed in microglia by Tmem119-CreERT2 (*Tmem119*^*CreERT2*^*hM3Dq-mCitrine*^*LSL/LSL*^ mice)^54^, to induce thalamic microglial reactive changes by local CNO injection into the thalamus via implanted cannula (Fig. 2h). We chose *Tmem119*^*CreERT2*^ mice because Tmem119-CreERT2 targets microglia more specifically than other microglia-targeting Cre drives (e.g., Cx3Cr1-CreER, P2ry12-CreER), with few Cre-dependent recombination in border-associated and peripheral macrophages^55,56^. To further eliminate possible contributions from rare peripheral macrophages that can be targeted by Tmem119-CreERT2^56^, tamoxifen injections were followed by a 30-day turnover period for peripheral macrophages^55,57–59^, restricting DREADD expression in long-lived microglia but not in peripheral macrophages. Finally, CNO was injected multiple times into the thalamus via implanted cannula to activate DREADD in microglia. DREADD activation in uninjured mice induced reactive microglial changes in the thalamus (Fig. 2i), promoted microglial engulfment of PSD95 synaptic proteins (Supplementary Fig. 6e), reduced overall numbers of glutamatergic synapses (Supplementary Fig. 6f), and impaired NOR and associated neuronal c-Fos expression (Fig. 2j and Supplementary Fig. 6g). These results demonstrate that reactive microglial changes in the thalamus are sufficient to produce NOR deficits and neuronal dysfunction similar to those observed in cortically injured mice.

### A distinct microglial state with high expression of *Cd9* and phagocytosis-related genes increases in the thalamus after cortical damage

**Fig. 3:**
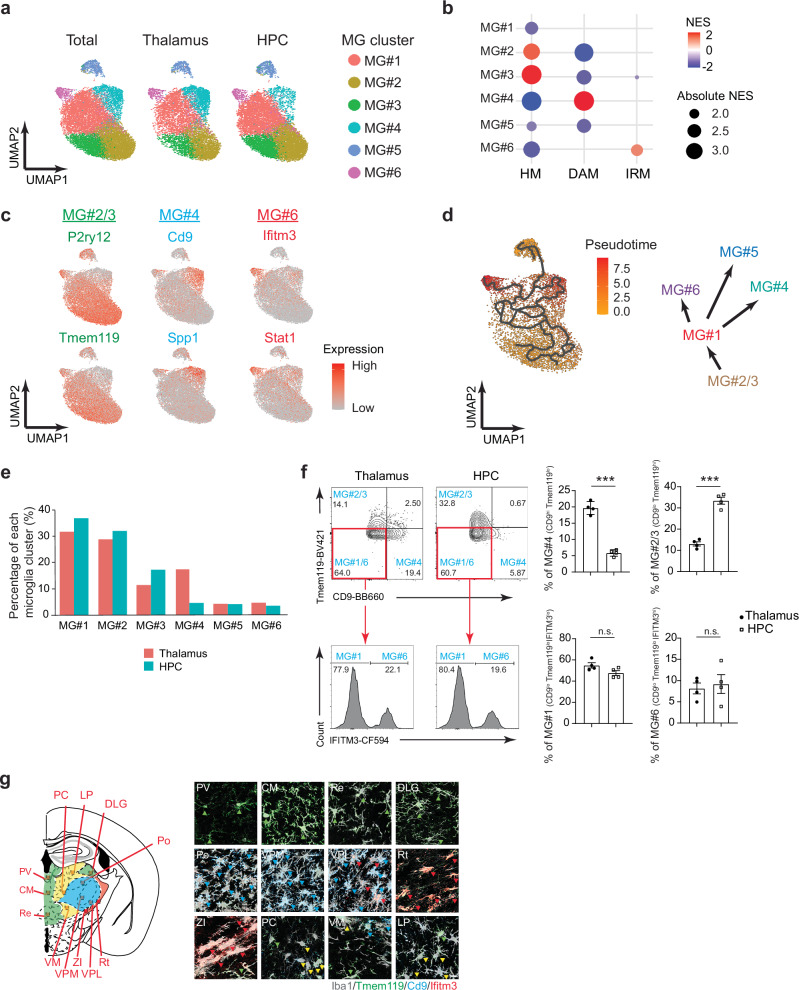
Microglia with high phagocytic capability are enriched in the thalamic inflammation secondary to cortical damage. **a** Uniform manifold approximation and projection (UMAP) plot highlighting 6 microglia clusters after CAI. **b** Dot plots showing enrichment of gene expression features for HM, DAM, and IRM across 6 microglia clusters based on the gene set enrichment analysis (GSEA). The circle sizes reflect absolute NES, and their colors indicated positive (red) and negative (blue) values. The data are corrected for multiple comparisons with the Benjamini–Hochberg method, and only the significant results (at adjusted *p*-values below 0.05) are shown. **c** Feature plots showing the genes enriched in MG#2/3 (*P2ry12*, *Tmem119*), MG#4 (*Cd9, Spp1*), MG#6 (*Ifitm3* and *Stat1*) across all the microglia clusters. **d** Pseudo-time analysis showing a cell transition trajectory from MG#2/3 to MG#4, #5, or #6 via MG#1, and a schematic diagram showing how MG#2 and #3 transit to MG#4, #5, and #6 via MG#1. **e** Bar graphs showing the percentages of each microglial cluster in the thalamus (red) and hippocampus (blue), with the percentages reaching 100% for each brain region. **f** Representative flow cytometry plots (left) and quantification (right) of microglial clusters in the thalamus and the HPC 21 days after CAI. Microglia are defined as live singlet CD11b^+^CD45^+^Ly6C^−^ cells (gating strategy available in Supplementary Fig. 4c). MG#2/3, MG#4, and MG#1/6 are identified as CD9^lo^Tmem119^hi^, CD9^hi^Tmem119^lo^, and CD9^lo^Tmem119^lo^. MG#6 is further defined by Ifitm3 expression among CD9^lo^Tmem119^lo^ populations. Quantification data show % of parent population (*n* = 4 mice per group). **g** Spatial distribution of MG#1, MG#2/3, MG#4, and MG#6 in the thalamus 21 days after CAI. Brain areas predominantly occupied by microglia with distinct states are shown with respective colors: MG#1 (yellow), MG#2/3 (green), MG#4 (blue), and MG#6 (red). Representative images of MG#1 (Tmem119^lo^CD9^lo^ Iba1^+^, yellow arrowheads), MG#2/3 (Tmem119^hi^ Iba1^+^, green arrowheads), MG#4 (CD9^hi^ Iba1^+^, blue arrowheads), and MG#6 (Ifitm3^hi^ Iba1^+^, red arrowheads) are shown in each thalamic subregion: paraventricular nucleus (PV), central medial nucleus (CM), nucleus reuniens (Re), dorsal lateral geniculate nucleus (DLG), posterior nucleus (Po), ventral posteromedial nucleus (VPM), ventral posterolateral nucleus (VPL), reticular nucleus (Rt), zona incerta (ZI), paracentral nucleus (PC), ventromedial nucleus (VM), and lateral posterior nucleus (LP). Scale bar, 10 µm. Representative data from *n* = 3 mice are shown. In bar graphs, data are shown as the mean ± s.e.m, and each dot represents an individual animal. *n* indicates individual mice. Data were collected from a single cohort with multiple mice (*n* = 3–4). ***p < 0.001; n.s. not significant (unpaired two-tailed t-test). Further details of the statistical analysis are available in the Supplementary Data S8. Source data are provided as a Source data file.

To better understand the molecular mechanisms underlying thalamic microglial changes in injured mice, we performed single-cell RNA sequencing (scRNA-seq) of CD45^+^ cells in the thalamus and HPC of injured mice. We identified 19 transcriptionally distinct cell types and states, with 6 distinct states belonging to microglia (designated as MG#1-6) (Fig. 3a, Supplementary Fig. 7a, and Supplementary Data S1). The 6 distinct microglial states were then compared to previously reported microglial states, including homeostatic microglia (HM), DAM and other DAM-related microglia, and interferon-responsive microglia (IRM) (Fig. 3b, c, Supplementary Fig. 7b, c, and Supplementary Data S2)^8^. MG#2 and #3 were enriched with HM-related genes, such as *P2ry12* and *Tmem11*9. MG#4 was enriched with DAM-related genes, such as *Cd9*, *Spp1, and Spp1*. MG#6 was enriched with IRM-related genes, such as *Ifitm3* and *Stat1*. MG#1 had an intermediate state that partially overlapped with HM, DAM, and IRM. MG#5 was different from the other states, showing a significantly reduced expression of ribosomal protein genes, such as *Rps16*, *Rps23*, and *Rpl19*. Pseudotime trajectory analysis revealed that MG#2 and #3 progressed toward MG#4, #5, or #6 through several common intermediate states in MG#1 (Fig. 3d and Supplementary Data S3).

Although MG#4 showed an overlap with DAM in their gene expression profiles, the expression of *Trem2*, a representative gene of DAM^60–62^, was not restricted to MG#4 (Supplementary Fig. 7d). We also observed that microglia with low CD9 and high Trem2 expression (CD9^lo^Trem2^hi^ microglia) were present both in the thalamus and the HPC, whereas microglia with high CD9 expression (both CD9^hi^Trem2^lo^ and CD9^hi^Trem2^hi^ microglia) were enriched in the thalamus (Supplementary Fig. 7e, f). These data suggested that MG#4 might be a state different from Trem2 expressing DAM. Notably, MG#4 was more than three-fold enriched in the thalamus than in the HPC (Fig. 3e). We also found that the gene expression patterns of thalamic MG#4 was distinct from those of HPC MG#4; genes related to phagocytosis (*Spp1*, *Cd9*, and *Lpl)* and lysosomes (*Cts7, Ctsl, Ctsb*, and *Ctsd*) were significantly higher in the thalamic MG#4 than the HPC MG#4 (Supplementary Fig. 7g and Supplementary Data S4). These findings indicate MG#4 in the thalamus has enhanced phagocytotic activities compared to those in the HPC.

These distinct microglia states in the thalamus and the HPC were confirmed by flow cytometry (Fig. 3f). Within the CD45^+^CD11b^+^Ly6C^−^ population, CD9^hi^Tmem119^lo^ microglia corresponding to MG#4 were markedly increased in the thalamus compared to the HPC, while CD9^lo^Tmem119^hi^ microglia corresponding to MG#2 and MG#3 were reduced. Among CD9^lo^Tmem119^lo^ microglia, we identified Ifitm3^hi^ cells as MG#6 and Ifitm3^lo^ cells as MG#1, neither of which exhibited region-specific changes. Immunohistochemistry further supported these findings, showing a substantially higher abundance of CD9^hi ^Iba1^+^ cells in the thalamus than in the HPC (Supplementary Fig. 7h).

We next examined the spatial distribution of microglia with distinct states in the thalamus of injured mice by immunohistochemistry (Fig. 3g). MG#2 and #3, visualized as Tmem119^hi^ Iba1^+^ cells, were found in a region distant from the center of the accumulation of microglia, such as the paraventricular nucleus (PV), central medial nucleus (CM), nucleus reuniens (Re), and dorsal lateral geniculate nucleus (DLG). MG#4, visualized as CD9^hi^ Iba1^+^ cells, were distributed around the posterior nucleus (Po), ventral posteromedial nucleus (VPM), and ventral posterolateral nucleus (VPL), regions where microglial morphological changes were most remarkable. MG#6, visualized as Ifitm3^hi^ Iba1^+^ cells, was found in the reticular nucleus (Rt) and zona incerta (ZI). MG#1, visualized as cells expressing Tmem119 and CD9 at lower levels, was observed in the paracentral nucleus (PC), ventromedial nucleus (VM), and lateral posterior nucleus (LP). In summary, MG#4 (CD9^hi^) was highly accumulated in the area where the most remarkable microglial morphological changes and the highest expression of Iba1 were observed (blue area); MG#6 (Iftm3^hi^) was located in the periphery (pink area); MG#2 and #3 (Tmem119^hi^) were located at distant sites (green area); and MG#1 (Tmem119^lo^CD9^lo^) was located between the areas occupied by MG#4 and #2/3 (yellow area) (Fig. 3g). The time-course immunohistochemistry analysis also revealed that CD9^hi^ Iba1^+^ cells were nearly absent in the intact thalamus and on day 7 but emerged by day 14 and increased toward day 21 (Supplementary Fig. 7i). These spatial and temporal data indicate that MG#4 (CD9^hi ^Iba1^+^ cells) in the inflammation core plays a pivotal role in thalamic inflammation and neuronal dysfunction after cortical damage.

### Synapse-engulfing thalamic microglia develop from reactive microglia precursors in a CD9-dependent manner and drive recognition memory deficits

**Fig. 4:**
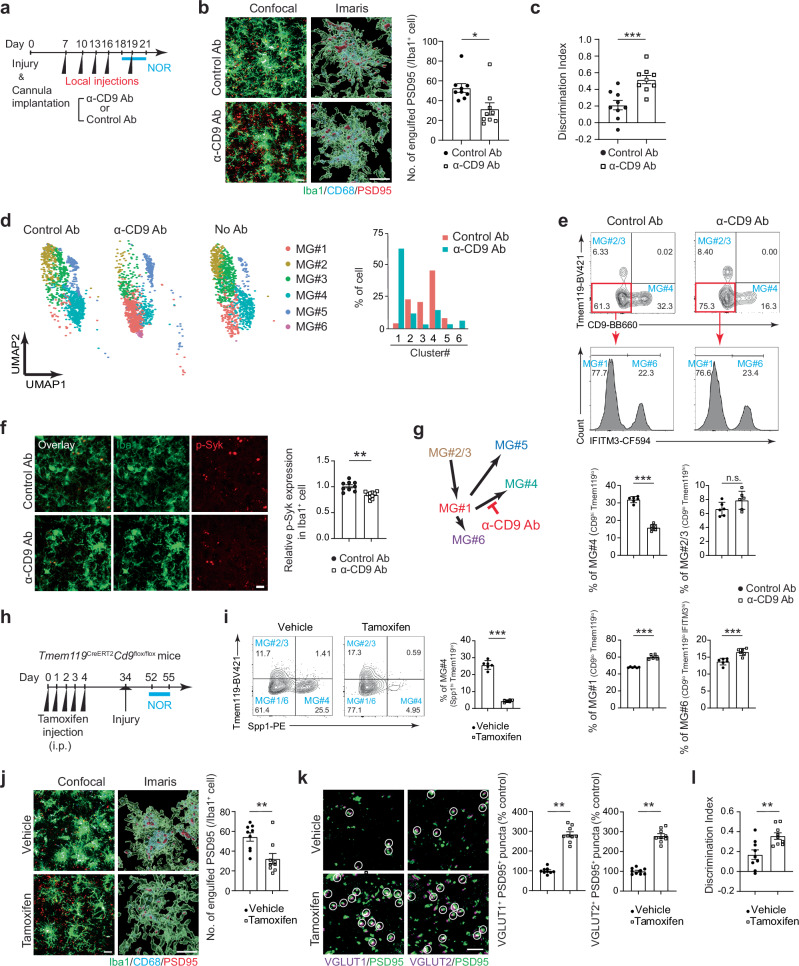
CD9 facilitates the generation of synapse-engulfing microglia from intermediate microglia, synaptic loss, and cognitive impairment after cortical damage. **a** Experimental timeline for intra-thalamic local injections of anti-CD9 Ab or control Ab via implanted cannulas into mice receiving CAI. **b** Representative confocal and Imaris-reconstructed 3D images (left) and quantification (right) of PSD95 engulfment by Iba1^+^ cells in the thalamus from the control Ab-injected and the anti-CD9 Ab-injected mice 21 days after CAI. Scale bars, 10 μm. The number of engulfed PSD95 puncta inside CD68^+^ lysosomes is quantified per cell (*n* = 9 mice per group). **c** Discrimination index in the NOR test for the control Ab-injected group (*n* = 9 mice) and the anti-CD9 Ab-injected group (*n* = 10 mice). **d** UMAP plots highlighting 6 microglia clusters in the thalamus in the control Ab-injected group and the anti-CD9 Ab-injected group after CAI. No Ab is the thalamus data from Fig. 3a. The bar graphs show the percentages of each microglial cluster in the control Ab group and the anti-CD9 Ab group, with the percentages reaching 100% for each group. **e** Representative flow cytometry plots (left) and quantification (right) of microglial clusters in the thalamus from control Ab- and anti-CD9 Ab-treated mice 21 days after CAI. Microglia are defined as live singlet CD11b^+^CD45^+^Ly6C^−^ cells (gating strategy available in Supplementary Fig. 4c). MG#2/3, MG#4, and MG#1/6 are defined as described in Fig. 3f. Quantification data show % of parent population (*n* = 6 mice per group). Antibodies used to detect CD9 are from different clones than those used for injections. **f** Representative images of phosphorylated Syk (p-Syk) signals in Iba1^+^ cells in the thalamus from the control Ab-injected and the anti-CD9 Ab-injected mice 21 days after CAI. Quantification data show the p-Syk levels in Iba1^+^ cells in the anti-CD9 Ab group (*n* = 10 mice) relative to the control Ab group (*n* = 9 mice). Scale bar, 10 µm. **g** A schematic diagram showing how MG#2 and #3 transition to MG#4, #5, and #6 via MG#1, and how anti-CD9 Ab acts on this pathway. **h** Experimental timeline for microglia-specific CD9 deletion followed by cortical damage and NOR test. **i** Representative flow cytometry plots (left) and quantification (right) of microglial clusters in the thalamus 21 days after CAI in the vehicle- and tamoxifen-treated *Tmem119*^CreERT2^*Cd9*^flox/flox^ mice. Microglia are defined as live singlet CD11b^+^CD45^+^Ly6C^−^ cells (gating strategy available in Supplementary Fig. 4c). MG#2/3, MG#4, and MG#1/6 are defined as Spp1^lo^Tmem119^hi^, Spp1^hi^Tmem119^lo^, and Spp1^lo^Tmem119^lo^, as the *Cd9* gene is deleted in these mice. Quantification data show % of parent population (*n* = 6 mice per group). **j** Representative confocal and Imaris-reconstructed 3D images (left) and quantification (right) of PSD95 engulfment by Iba1^+^ cells in the thalamus from the vehicle- and tamoxifen-treated *Tmem119*^CreERT2^*Cd9*^flox/flox^ mice 21 days after CAI. Scale bars, 10 μm. The number of engulfed PSD95 puncta inside CD68^+^ lysosomes is quantified per cell (*n* = 9 mice per group). **k** Representative images (left) and quantification (right) of VGLUT1/PSD95 and VGLUT2/PSD95 synapses in the thalamus 21 days after CAI in the vehicle- and tamoxifen-treated group. Scale bar, 2 μm. Quantification data show the synapse density in the thalamus of the tamoxifen-treated group relative to that of the vehicle-treated group (*n* = 9 mice per group). **l** Discrimination index in the NOR test for the vehicle- and tamoxifen-treated group (*n* = 9 mice per group). In bar graphs, data are shown as the mean ± s.e.m, and each dot represents an individual animal. *n* indicates individual mice. For **b**, **c**, **f**, **j**–**l**, data were collected from 3 independent cohorts and pooled for analysis. For **e**, **i**, data were collected from 2 independent cohorts and pooled for analysis. For the others, data were collected from a single cohort. *p < 0.05, **p < 0.01, and ***p < 0.001; n.s. not significant (unpaired two-tailed t-test). Further details of the statistical analysis are available in the Supplementary Data S8. Source data are provided as a Source data file.

We then tested if MG#4 caused thalamic synapse loss through phagocytosis, thereby contributing to impaired recognition memory. Given that CD9 is a cell-surface protein readily detected by flow cytometry analysis (Fig. 3f), we reasoned that antibody-mediated CD9 blockade could inhibit MG#4 function. Previous studies demonstrated that anti-CD9 blocking Ab inhibited macrophage phagocytosis and related biological processes in vitro and in vivo^63–65^. Therefore, we delivered anti-CD9 blocking Ab locally into the thalamus via implanted cannulas to assess the impact of MG#4 inhibition on thalamic pathology and recognition memory deficits (Fig. 4a).

Repeated intra-thalamic anti-CD9 Ab injections from days 7 to 19 post-injury significantly reduced the number of CD9^hi^ microglia, the CD68^+^ lysosomal area per microglia, and the amount of PSD95 contained within the CD68^+^ compartment on day 21 (Fig. 4b and Supplementary Fig. 8a, b), indicating the attenuated microglial reactivities and synaptic phagocytosis. Accordingly, the numbers of glutamatergic synapses (VGLUT1^+^PSD95^+^ and VGLUT2^+^PSD95^+^ puncta), total neurons, and c-Fos expressing neurons increased in the thalamus of mice receiving anti-CD9 Ab (Supplementary Fig. 8c–e). Consistent with these immunohistochemistry data, CD9 blockade attenuated NOR deficits induced by cortical damage (Fig. 4c). These findings indicate that MG#4, comprising the majority of CD9^hi^ Iba1^+^ cells, drives synapse engulfment, synapse loss, and subsequent recognition memory deficits.

To determine how CD9 blockade affects MG#4, we used scRNA-seq to examine thalamic microglial states in the injured mice receiving anti-CD9 Ab or control Ab. Six microglial subclusters were identified in the antibody-injected injured mice, similar to the injured mice without antibody injections (Fig. 4d and Supplementary Fig. 8f, g). Anti-CD9 Ab injection markedly reduced the abundance of MG#4 while expanding MG#1 (Fig. 4d). These changes were confirmed by flow cytometry, which showed a significant decrease in CD9^hi^Tmem119^lo^ cells (MG#4) and a reciprocal increase in CD9^lo^Tmem119^lo^ cells (MG#1) within CD11b^+^CD45^+^Ly6C^−^ cells (Fig. 4e). CD9 blockade also reduced the phosphorylation of spleen tyrosine kinase (Syk), a known mediator of CD9 signaling^66,67^, in thalamic microglia (Fig. 4f), and reduced the expression of MG#4-associated genes in MG#1, including genes overlapping with the established Syk-dependent gene signatures (Supplementary Fig. 8h and Supplementary Data S5). Although a fraction of MG#4 persisted after anti-CD9 Ab treatment, these cells exhibited significantly diminished expression of phagocytosis- and lysosome-related genes (Supplementary Fig. 8i and Supplementary Data S6). Collectively, these findings indicate that CD9 blockade from day 7 to day 19 disrupts the transition of MG#1 into MG#4 and limits the acquisition of synapse phagocytosis capability (Fig. 4g).

To further dissect the effects of CD9 blockade on the dynamics of microglial state transitions, we pulse-labeled microglia with 5-ethynyl-2′-deoxyuridine (EdU) on day 7, prior to MG#4 emergence (Supplementary Fig. 7i), and tracked EdU^+^ cells on day 21 (Supplementary Fig. 9a). Although the total number of EdU^+^ Iba1^+^ cells was unchanged, anti-CD9 Ab injections significantly reduced the number of CD9^hi^ EdU^+^ microglia (Supplementary Fig. 9a). Continuous EdU treatments from day 7 to day 19 revealed no effect of anti-CD9 Ab injections on overall microglial proliferation (Supplementary Fig. 9b). Flow cytometry analysis did not detect any increase in cleaved Caspase-3^+^ apoptotic cells within MG#4 (Supplementary Fig. 9c). These data demonstrate that CD9 blockade primarily alters the microglial state transitions into MG#4 rather than influencing cell proliferation or survival (Fig. 4g).

Because macrophages are also Iba1^+^ cells and express Cd9 mRNA in the injured thalamus (Supplementary Fig. 10a), we also examined the effects of CD9 blockade on macrophage states. Macrophages were divided into 6 clusters (Supplementary Fig. 10b), and MP#1 was enriched with the genes associated MG#4 (Supplementary Fig. 10c). Pseudotime trajectory analysis indicated MP#2 and MP#3 as intermediate states (Supplementary Fig. 10d). Notably, anti-CD9 Ab reduced MP#1 and increased MP#2 and MP#3 (Supplementary Fig. 10e), and the flow cytometry analysis confirmed the reduction of CD9^hi^ cells within CD11b^+^CD45^+^Ly6C^+^ cell populations in the thalamus (Supplementary Fig. 10f). These results suggest that CD9 blockade inhibits the generation of phagocytic macrophages (MP#1) from their precursors (MP#2 and MP#3) (Supplementary Fig. 10d), analogous to its inhibitory effects on MG#4 generation from MG#2 and MG#3.

To directly test the role of MG#4 (CD9^hi^ microglia) in synaptic loss and recognition memory deficits, we generated *Tmem119*^*CreERT2*^*Cd9*^*flox/flox*^ conditional knockout mice and restricted CD9 deletion to microglia with tamoxifen injections followed by a 30-day turnover period for peripheral macrophages (Fig. 4h)^55,57–59^. Flow cytometry analysis confirmed the significant loss of CD9 expression in CD45^+^CD11b^+^Ly6C^−^ cells, whereas CD45^+^CD11b^+^Ly6C^+^ cells retained CD9 expression (Supplementary Fig. 10g). Consistent with the findings from CD9 blockade experiments, this microglia-specific CD9 deletion significantly reduced MG#4 (defined as Spp1^hi^Tmem119^lo^ cells, as CD9 is deleted in these mice), impaired microglial synaptic engulfment, and increased thalamic glutamatergic synapse numbers (Fig. 4i–k and Supplementary Fig. 10h). Mice with microglia-specific CD9 deletion also showed improved NOR performance (Fig. 4l). Together, these results further support that CD9^hi^ microglia (MG#4) are key mediators of thalamic synapse loss and cognitive impairment after cortical damage.

### Axl mediates thalamic synapse engulfment by microglia in injured mice

**Fig. 5:**
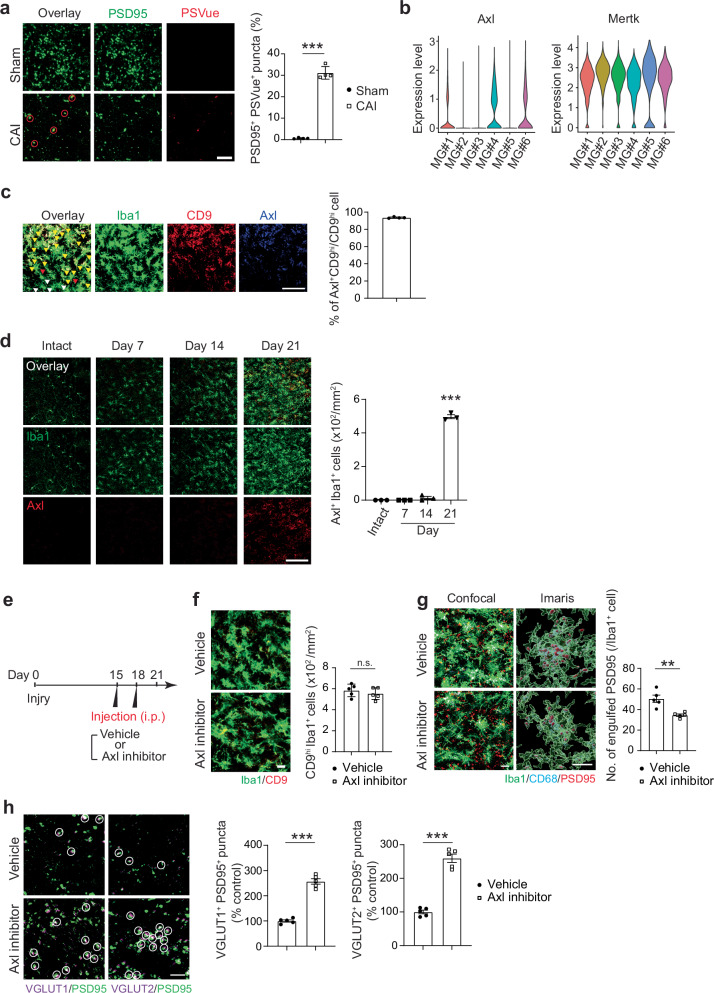
Axl mediates microglial synapse phagocytosis in the thalamus. **a** Representative images (left) and quantification (right) of PSVue^+^ PSD95 puncta in the thalamus 21 days after CAI or sham surgery. Scale bar, 2 µm. Quantification data show the density of PSVue^+^ PSD95 puncta in the CAI group relative to the sham group (*n* = 4 mice per group). **b** Violin plots showing the mRNA expression level of Axl and Mertk in each microglial subcluster in the thalamus 21 days after CAI. **c** Representative images (left) and quantification (right) of Axl^+^ and CD9^hi^ cells in the thalamus 21 days after CAI. Scale bar, 50 µm. Quantification data show % of CD9^hi^Axl^+^Iba1^+^ cells within CD9^hi^ Iba1^+^ cells (*n* = 4 mice). **d** Representative images (left) and quantification (right) of Iba1^+^ and Axl^+^ cells in the thalamus of mice 7, 14, and 21 days after CAI and of intact mice. Scale bar, 100 µm. Quantification data show the density of Axl^+^ Iba1^+^ cells in the thalamus for each group (*n* = 3 mice). **e** Experimental timeline of Axl inhibitor treatment after cortical damage. **f** Representative images (left) and quantification (right) of CD9^hi^ Iba1^+^ cells in the thalamus of the vehicle-injected and the Axl inhibitor-injected mice 21 days after CAI. Scale bar, 10  μm. Quantification data show the density of CD9^hi^ Iba1^+^ cells in the thalamus for each group (*n* = 5 mice per group). **g** Representative confocal and Imaris-reconstructed 3D images (left) and quantification (right) of PSD95 engulfment by thalamic Iba1^+^ cells in mice injected with vehicle or Axl inhibitors. Scale bars, 10 μm. The number of engulfed PSD95 puncta inside CD68^+^ lysosomes is quantified per cell (*n* = 5 mice per group). **h** Representative images (left) and quantification (right) of VGLUT1/PSD95 and VGLUT2/PSD95 synapses in the thalamus of the vehicle- and the Axl inhibitor-injected mice 21 days after CAI. Scale bars, 2 μm. Quantification data show the synapse density in the thalamus of the Axl inhibitor-injected group relative to that of the vehicle-injected group (*n* = 5 mice per group). In bar graphs, data are shown as the mean ± s.e.m, and each dot represents an individual animal. *n* indicates individual mice. Data were collected from a single cohort. **p < 0.01, ***p < 0.001; n.s. not significant [unpaired two-tailed t-test (**a**, **f**–**h**) and one-way ANOVA with post-hoc Dunnett’s test (**d**)]. Further details of the statistical analysis are available in the Supplementary Data S8. Source data are provided as a Source data file.

We next investigated the mechanisms by which MG#4 engulfed thalamic synapses. In the thalamus of injured mice, phosphatidylserine (PS) was prominently exposed on PSD95^+^ synapses (Fig. 5a). Because PS is recognized by TAM‑family receptor tyrosine kinases during the initiation of phagocytosis^68^, we examined the expression of Axl and Mer, the two TAM receptors expressed in microglia^69^. Previous studies have shown that Axl is upregulated in reactive states, such as DAM, whereas Mer is involved in homeostatic phagocytosis^69,70^. Consistent with these reports, Axl mRNA was enriched in MG#4 in our scRNA-seq data of thalamic microglia (Fig. 5b). In contrast, Mertk mRNA (encoding Mer protein) was expressed broadly across microglial clusters.

We therefore tested if Axl mediated synapse engulfment by CD9^hi^ thalamic microglia. Axl protein was expressed in >90% of MG#4 (CD9^hi ^Iba1^+^ microglia) (Fig. 5c), and Axl^+ ^Iba1^+^ microglia appeared only at the late stage (day 21) after cortical damage (Fig. 5d), suggesting the role of Axl in MG#4-mediated synapse phagocytosis. To assess the functional contribution of Axl, we intraperitoneally administered the selective Axl inhibitor, Bemcentinib/R428, into the injured mice. Axl inhibition did not alter the overall abundance of CD9^hi^ microglia in the thalamus, but reduced synapse engulfment and increased thalamic glutamatergic synapse numbers (Fig. 5e–h). These findings indicate that Axl promotes synapse engulfment by MG#4 (CD9^hi^ microglia) in the thalamus of injured mice.

### Blood-brain barrier (BBB) disruption and IgG extravasation in the thalamus promote the generation of synapse engulfing microglia via Fcγ receptor III signaling

**Fig. 6:**
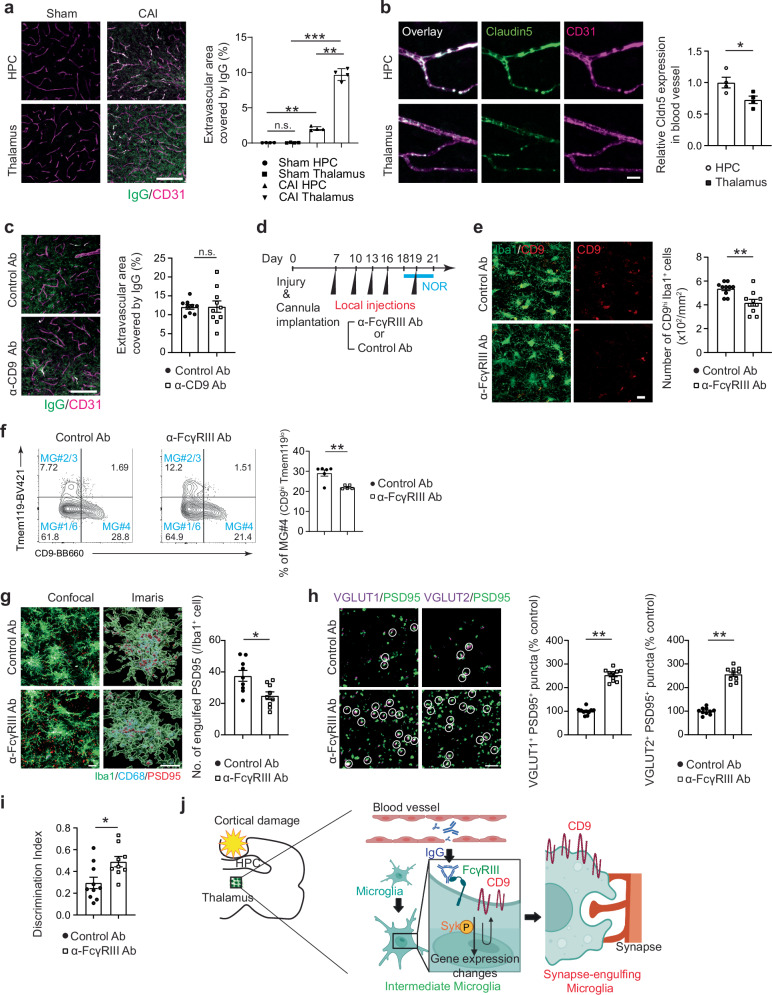
IgG extravasation in the thalamus promotes the generation of synapse-engulfing microglia via FcγRIII signaling. **a** Representative images (left) and quantification (right) of IgG and CD31^+^ blood vessels of the thalamus and the HPC 21 days after CAI or sham surgery. Scale bar, 100 μm. Quantification data show the percentages of area covered by extravascular IgG for the HPC and the thalamus (*n* = 4 mice per group). **b** Representative images (left) and quantification (right) of Claudin5 (Cldn5) and CD31^+^ blood vessels in the thalamus and the HPC 21 days after CAI. Scale bar, 10 μm. Quantification data show the Claudin5 expression levels in CD31^+^ blood vessels in the thalamus relative to the HPC (*n* = 4 mice per group). **c** Representative images (left) and quantification (right) of IgG and CD31^+^ blood vessels in the thalamus from the control Ab- and anti-CD9 Ab-injected mice 21 days after CAI. Scale bar, 100 μm. Quantification data show the percentages of area covered by extravascular IgG relative to total IgG for the control Ab-injected (*n* = 10 mice) and the anti-CD9 Ab-injected (*n* = 9 mice) group. **d** Experimental timeline for intra-thalamic local injections of anti-FcγRIII Ab or control Ab via implanted cannulas into mice receiving CAI. **e** Representative images (left) and quantification (right) of CD9^hi ^Iba1^+^ cells in the thalamus from the control Ab- and anti-FcγRIII Ab-injected mice 21 days after CAI. Scale bar, 10 μm. Quantification data show the number of CD9^hi^ Iba1^+^ cells in the thalamus of the control Ab-injected (*n* = 10 mice) and anti-FcγRIII Ab-injected (*n* = 9 mice) group. **f** Representative flow cytometry plots (left) and quantification (right) of microglial clusters in the thalamus from the control Ab- and anti-FcγRIII Ab-injected mice 21 days after CAI. Microglia are defined as live singlet CD11b^+^CD45^+^Ly6C^−^ cells (gating strategy available in Supplementary Fig. 4c), and microglial clusters are identified as described in Fig. 3f. Quantification data show % of parent population (*n* = 6 mice for the control Ab-injected group; *n* = 5 mice for the anti-FcγRIII Ab-injected group). **g** Representative confocal and Imaris-reconstructed 3D images (left) and quantification (right) of PSD95 engulfment by Iba1^+^ cells in the thalamus from the control Ab- and anti-FcγRIII Ab-injected mice 21 days after CAI. Scale bars, 10 μm. The number of engulfed PSD95 puncta inside CD68^+^ lysosomes is quantified per cell (*n* = 10 mice for the control Ab-injected group; *n* = 9 mice for the anti-FcγRIII Ab-injected group). **h** Representative images (left) and quantification (right) of VGLUT1/PSD95 and VGLUT2/PSD95 synapses in the thalamus from the control Ab- and anti-FcγRIII Ab-injected mice 21 days after CAI. Scale bar, 2 μm. Quantification data show the synapse density in the thalamus of the anti-FcγRIII Ab-injected group (*n* = 9 mice) relative to that of the control Ab-injected group (*n* = 10 mice). **i** Discrimination index in the NOR for the control Ab-injected (*n* = 10 mice) and anti-FcγRIII Ab-injected (*n* = 9 mice) group. **j** Graphic illustration showing how synapse-engulfing microglia (MG#4) develop in response to extravasated IgG via FcγRIII and CD9 signaling, engulfing synapses and impairing neuronal function. Created in BioRender. Kano, S. (2026) BioRender.com/o5w0c0o. In bar graphs, data are shown as the mean ± s.e.m, and each dot represents an individual animal. *n* indicates individual mice. For **c**, **e**, **g**–**i**, data were collected from 3 independent cohorts and pooled for analysis. For **f**, data were collected from 2 independent cohorts and pooled for analysis. For the others, data were collected from a single cohort. *p < 0.05, **p < 0.01, and ***p < 0.001; n.s. not significant [two-way ANOVA followed by Tukey–Kramer’s post-hoc test (**a**) and unpaired two-tailed t-test (**b**, **c**, **e**, **f**–**i**)]. Further details of the statistical analysis are available in the Supplementary Data S8. Source data are provided as a Source data file.

We next explored the mechanisms underlying the selective appearance of CD9^hi^ microglia in the thalamus following cortical damage. Previous studies in human TBI patients and rodent models have reported extravasation of γ-immunoglobulins (IgG) related to BBB leakage during the chronic phase of cortical brain injuries^71–73^, prompting us to examine IgG extravasation in the thalamus of injured mice. We observed substantial accumulation of extravasated IgG in the thalamus, but subtle or none in the HPC, mPFC, and PRh, 21 days after cortical damage (Fig. 6a and Supplementary Fig. 11a, b). The expression of Claudin 5, a tight junction protein, was significantly reduced, while the expression of VCAM1, a cell adhesion molecule associated with increased BBB permeability, was elevated in the thalamus compared to the HPC (Fig. 6b and Supplementary Fig. 11c). IgG extravasation was evident as early as day 7, preceding the appearance of MG#4 (Supplementary Figs. 7i and 11d) and was still detected on day 63, similar to CD9^hi^ microglia (Supplementary Fig. 11e, f). Notably, intra-thalamic injections of anti-CD9 Ab did not reduce IgG extravasation (Fig. 6c). Thus, BBB disruption and subsequent IgG extravasation preceded the generation of synapse-engulfing thalamic microglia (MG#4).

CD9 has been reported to be functionally associated with FcγRIII signaling in macrophages^66^, suggesting that IgG-FcγRIII signaling may promote CD9^hi^ microglia generation. Indeed, FcγRIII was expressed both in CD9^hi^ and CD9^lo^ microglia (Supplementary Fig. 11g). Thus, we speculated that FcγRIII signaling might mediate the effects of extravasated IgG on thalamic microglial state transition. To address this, we locally blocked FcγRIII in the thalamus via intra-thalamic anti-FcγRIII Ab injections (Fig. 6d). FcγRIII blockade reduced the number of CD9^hi^ thalamic microglia, attenuated microglial engulfment of synaptic component PSD95, increased thalamic glutamatergic synapses, and improved NOR in injured mice (Fig. 6e–i). The link between IgG extravasation and CD9^hi^ microglia in the thalamus was not unique to cortical damage. We observed similar IgG extravasation and CD9^hi^ microglia accumulation in the thalamus of 5×FAD mice, a mouse model of amyloid pathology relevant to Alzheimer’s disease (Supplementary Fig. 11h, i). CD9 blockade also improved NOR deficits in 5×FAD mice (Supplementary Fig. 11j, k). Altogether, these findings suggest that BBB disruption and IgG extravasation in the thalamus facilitate the generation of synapse-engulfing microglia (MG#4/CD9^hi^ microglia) via FcγRIII signaling, leading to synaptic loss, neuronal dysfunction, and recognition memory deficits in injured mice (Fig. 6j).

## Discussion

In this study, we demonstrate that reactive microglial changes in the thalamus during secondary injury are crucial for neuronal dysfunction and recognition memory deficits in mice receiving cortical damage. We discover synapse-engulfing microglia (MG#4), marked by high CD9 expression and phagocytosis- and lysosome-related gene programs, that are enriched in the thalamus in the chronic phase of cortical damage. CD9^hi^ MG#4 microglia phagocytose thalamic synapses in an Axl-dependent manner, causing synapse loss and recognition memory deficits. We further show that the BBB disruption and subsequent IgG extravasation promote the generation of MG#4 via FcγRIII signaling. Thus, our study reveals a spatially and temporally restricted microglial state that mediates the transition of acute cortical brain injury into chronic cognitive impairment.

Diverse microglial states have been defined by their gene expression signatures under various neuroinflammatory conditions^5–8^. Nevertheless, their impact on neuropathology and cognitive impairment has not been fully understood. A major barrier has been the lack of tools to allow either region- or state-specific manipulation of microglia in vivo: pharmacological depletion (e.g., PLX compounds)^74^ and Cre transgenic mice (e.g., *Cx3Cr1*^CreERT2^ mice) broadly target microglia, obscuring the roles of discrete microglial populations. As a result, studies in rodent cortical injury models have yielded conflicting results—some reporting that microglial repopulation after depletion improves cognitive outcomes via neurotrophic factor release^30,31^, while others conclude that microglial depletion prevents the development of cognitive impairment^32^. By using intracranial delivery of blocking antibodies to confine the effects to defined regions, our study demonstrates that thalamus‑restricted reactive microglia are the critical drivers of cortical damage-induced recognition memory deficits. As regional heterogeneity in microglia underlies various brain disorders, similar approaches may be helpful in understanding the relative contributions of region-specific microglia to brain pathology and dysfunction in other disease contexts.

Our scRNA-seq analysis has revealed the microglia with multiple distinct states in the two representative secondary injury areas (the thalamus and the HPC) in the chronic phase of cortical damage, including CD9^hi^ microglia (MG#4), Ifitm3^hi^ IFN-responsive microglia (MG#6), Tmem119^hi^ homeostatic microglia (MG#2, #3), and microglia with intermediate phenotype (MG#1). Although MG#4 is characterized by the enriched expression of DAM markers, such as *Cd9* and *Spp1*, the expression of *Trem2* is not selectively enriched in MG#4. MG#4 also causes synapse loss and impairs cognition, while DAM plays a neuroprotective role^5^. These results suggest that MG#4 is functionally distinct from DAM. Previous studies report that TREM2 is required for reactive microglia to become DAM^5^. Although MG#4 and DAM have an overlapped gene expression signature, our data reveal that CD9 expression is more confined to MG#4, while Trem2 is expressed across multiple reactive states in the thalamic microglia in the chronic phase of cortical damage. Thus, CD9 and TREM2 may have differential contributions to the generation of microglia with vigorous phagocytic activities. Both CD9 and TREM2 are cell surface molecules enriched in specific microglial states. Further studies focusing on the role of microglial state-specific cell surface molecules may help reveal the mechanisms underlying the transition of reactive microglial states to specific phenotypes, such as phagocytic microglia.

Reactive microglia also show distinct spatial localization patterns within the thalamus depending on their states; synapse engulfing CD9^hi^ microglia (MG#4) and IFN-responsive microglia (MG#6) are localized to distinct thalamic subregions in an almost mutually exclusive manner. Several recent studies using scRNA-seq have also shown the presence of microglia with distinct transcriptional states in primary injury areas of TBI^33,34,36,37^. However, reactive microglial changes in secondary injury areas remain poorly characterized. Thus, our scRNA-seq data provide molecular insight into reactive microglial changes in secondary injury areas associated with cognitive impairment after cortical brain injury. Future studies would dissect the relationships among these spatially localized distinct microglial states.

Mechanisms to induce synapse engulfing microglia are not fully understood. Our data demonstrate that CD9 signaling is crucial for the development of a distinct microglial state involved in synapse phagocytosis (MG#4) in the thalamus of injured mice. Notably, Syk, a downstream kinase for CD9 signaling, has been recently shown to play a critical role in microglial phagocytosis of neurotoxic materials in mouse models of Alzheimer’s disease and multiple sclerosis^75,76^. As CD9 is reported to be expressed in several microglial states, such as DAM, under Alzheimer’s disease and other neurodegenerative conditions, CD9-dependent generation of phagocytic microglia may be a shared mechanism across multiple disease contexts. Indeed, we found similar CD9^hi^ microglia in the thalamus of 5×FAD mice. Our data also show that CD9 blockade inhibits the expression of a broad range of Syk-independent genes related to MG#4 in MG#1. As a tetraspanin, CD9 can form complexes with multiple membrane proteins within tetraspanin-enriched microdomains and stabilizes their surface expression^77^. Thus, CD9 may influence multiple membrane proteins and their intracellular signaling to control microglial phenotype.

Our findings also reveal that IgG/FcγRIII signaling facilitates the induction of phagocytic phenotypes in microglia. Engagement of activating type I FcγR, such as FcγRIII, and stimulation of downstream signaling pathways by IgG has been shown to induce transcriptions of genes encoding pro-inflammatory mediators, phagocytosis, and lysosomes in neutrophils, macrophages, and other phagocytic cells^78^. FcγR signaling is also known to play a critical role in generating osteoclasts, which are highly phagocytic cells with elevated expression of Osteopontin (Spp1) and CD9^79–81^. Thus, FcγR signaling-mediated induction of phagocytic phenotypes may be shared features across multiple myeloid lineage cells.

We also find that BBB changes (reduced Claudin 5 expression and increased VCAM1 expression) and IgG extravasation, which are likely to be driven by inflammatory changes in endothelial cells^82^, occur predominantly in the thalamus during the chronic phase of cortical brain damage. One possible explanation for this selectivity of vascular inflammation and subsequent BBB disruption in the thalamus is that damage to the highly dense connections between the thalamus and primary cortical injury areas results in the release of excessive axonal debris, inducing massive inflammation involving both microglia and astrocytes and their production of cytokines and chemokines, which contribute to endothelial cell inflammation. Another possibility is that microglia in the thalamus may be more prone to cause endothelial cell inflammation upon reactive changes than those in other brain regions. Regional differences in microglia, as recently reported^83,84^, may also underlie their lower threshold toward reactive changes, leading to endothelial cell inflammation and BBB disruption in the thalamus. Finally, regional differences in endothelial cell molecular phenotypes, as recently described^85–87^, may also contribute to their susceptibility to inflammatory changes, although further research is warranted in this area.

Human positron emission tomography (PET) imaging studies using a translocator protein (TSPO) ligand have consistently revealed persistent thalamic inflammation long after TBI, which is correlated with cognitive impairment^20,52^. Postmortem brain studies have also demonstrated that thalamic damage and neuroinflammation are prevalent in TBI and associated with worse outcomes^88,89^. Rodent studies have also observed significant microglial changes in the thalamus post-TBI^37,90^. Although our primary model used in this study does not recapitulate clinically observed TBI, our findings align with these previous patient observations and further elucidate a pivotal role for thalamic microglial changes in the progression from acute brain damage to chronic cognitive impairment. Further studies on human TBI would reveal the detailed localization of microglia with distinct states in the brains after cortical injuries and advance our understanding of microglial contributions to cognitive impairment, contributing to the development of effective therapeutic interventions to prevent post-TBI cognitive impairment.

This study has several limitations. First, our primary model of cortical damage, CAI, is a surgical resection of cortical tissues, and does not include an impact clinically observed in TBI. Although widely used CCI models recapitulated the thalamic microglial changes in CAI, caution may be required to interpret our findings in terms of the long-term consequences of TBI. Second, antibody delivery provides anatomical specificity but does not fully resolve cell-type specificity. Indeed, we observed local administration influenced multiple myeloid populations within the injected region. Although our microglia-selective genetic deletion complements this limitation in cell-type specificity, future studies that combine region-restricted genetic strategies with longitudinal profiling will be important to define the kinetics of transitions among intermediate and phagocytic microglial states.

Despite these limitations, our findings provide a mechanistic framework linking delayed thalamic BBB disruption and IgG leakage to the emergence of a CD9-dependent, synapse-engulfing microglial program that drives neuronal dysfunction and recognition memory deficits in mice after cortical damage. Notably, similar associations between IgG extravasation and thalamic CD9^hi^ microglia were also observed in 5×FAD mice, suggesting that IgG/FcγRIII and CD9 signaling may represent a convergent pathway through which vascular dysfunction promotes pathological microglial synapse elimination across multiple disorders. Targeting BBB integrity, IgG/FcγRIII signaling, CD9 signaling, and Axl-dependent mechanisms may therefore offer therapeutic opportunities to mitigate chronic neuroinflammation and cognitive impairment following cortical brain injury.

## Methods

### Mice

All animal experiments were approved by and in compliance with the ethical regulations by the University of Alabama at Birmingham (UAB) Institutional Animal Care and Use Committee and Animal Research Resource (IACUC protocol numbers: 21539, 22522, 22992). C57BL/6J (#664), Tmem119-CreERT2 (*Tmem119*^*CreERT2*^, #031820), CAG-LSL-hM3Dq-mCitrine (*hM3Dq-mCitrine*^*LSL/LSL*^, #026220), 5×FAD [B6.Cg-Tg(APPSwFlLon, PSEN1*M146L* L286V)6799Vas/Mmjax, #034848], and FLPo (B6 ROSA26 Flpo, #012930) mice were purchased from the Jackson Laboratory. Cd9-floxed (*Cd9*^*fl/fl*^) mice were generated by crossing *Cd9*^*tm*1*a*H*mgu*^ mice (EMMA ID: EM:07694) [derived from European Mouse Mutant Archive (EMMA) via Institute Clinique de la Souris] with FLPo mice. *Tmem119*^*CreERT2*^*hM3Dq-mCitrine*^*LSL/LSL*^ mice and *Tmem119*^*CreERT2*^*Cd9*^*fl/fl*^ mice were generated by crossing *Tmem119*^*CreERT2*^ mice with *hM3Dq-mCitrine*^*LSL/LSL*^ mice and *Cd9*^*fl/fl*^ mice, respectively. To induce Cre recombination, these mice were injected with 0.1 mL of tamoxifen (20 mg/mL in corn oil; Sigma Aldrich, #T5648) for 5 consecutive days. Female mice were used for most of the experiments unless stated otherwise. All the mice were housed in specific pathogen-free facilities with ad libitum access to food and water under a standard light/dark cycle. During euthanasia, enough care was taken to minimize the suffering of the mice. For non-survival procedures, anesthesia was induced with isoflurane and maintained until spontaneous respiration completely ceased. A secondary physical method, including cervical dislocation and decapitation, was used to confirm death. For survival procedures, such as brain injuries and virus injections, anesthesia was induced and maintained with isoflurane. Carprofen or Buprenorphine was administered as pre- and post-operative analgesics.

### Cortical brain injuries

#### Cortical ablation injury (CAI)

Unilateral lesions of the cortex were induced as described previously^11,38,91–93^. A 3.0 mm-wide burr hole was made in the skull bone of anesthetized mice at a point midway between the lambda and bregma sutures and laterally midway between the central suture and temporalis muscle. Cortical ablation (for sensory motor cortex, 1.5 mm each from bregma to caudal and rostral, 3 mm to the right, and 1.0 mm in depth) was performed by aspiration with a sterilized pipette. Sham-operated mice also received a burr hole without cortical ablation. Compared to a CCI model, this injury damages the brain tissue by a surgical ablation (contusion) but does not provide any blow or violent shaking (concussion).

#### Controlled cortical impact (CCI)

Moderate CCI was performed as previously described by refs. ^30,31,94–96^. After mice were anesthetized, a 4.0-mm-wide burr hole was made in the skull bone at a point midway between the lambda and bregma sutures and laterally midway between the central suture and the temporalis muscle. After removal of the scalp, the tip of the 3-mm impactor (Leica) piston was angled and kept perpendicular to the exposed cortical surface. The cortical impact was given with the following parameters: impact speed, 3.5 m/s; deformation depth, 1.0 mm; and duration, 400 ms. Sham mice underwent the same craniotomy without cortical impact.

#### Repeated closed CCI

Repeated closed CCI was performed using a closed-skull CCI paradigm. On each injury day, mice were anesthetized, and a midline scalp incision was made to expose the skull. A 3-mm impactor tip was positioned vertically 1.5 mm lateral to bregma (right), and an impact was delivered with the following parameters: impact velocity, 5.0 m/s; deformation depth, 1.0 mm; and dwell time, 200 ms. At the end of each session, the scalp was sutured with 6-0 nylon, and mice were allowed to recover. This procedure (anesthesia, scalp incision, impact, and skin closure) was repeated once daily for 3 consecutive days (one impact per day). Sham mice underwent the same daily anesthesia, midline scalp incision/exposure, and skin closure without impact^97,98^.

### Cannula implantation

Immediately after CAI, while mice remained under continuous anesthesia and secured in a stereotaxic frame, a guide cannula was implanted through the CAI craniectomy window in the same surgical session (no additional burr hole was made). A 26‑gauge guide cannula (Plastics One) was implanted ipsilaterally into the right thalamus (AP −1.50 mm, ML +1.50 mm, DV −3.00 mm) or the right hippocampus (AP −1.50 mm, ML +1.50 mm, DV −1.50 mm), relative to bregma. The guide cannula was secured with miniature anchor screws and dental cement. To prevent clogging, a 32‑gauge dummy cannula (Plastics One) was inserted into the guide cannula and remained in place except during injections.

For intracranial injections via the implanted cannula, an internal injector cannula (Plastics One) extending 0.5 mm beyond the tip of the guide cannula was connected to a Hamilton syringe driven by a syringe pump. Reagents were infused at 200 nl/min for a total volume of 1 µl. The injector was left in place for 5 min after infusion to minimize backflow and then removed, after which the dummy cannula was reinserted.

### Intra-thalamic injections of antibodies and reagents

Antibodies and LPS (4 µg/µl; Sigma, L2880) were administered via implanted cannula following the indicated schedule. The following antibodies and respective isotype controls were injected: anti-CSF1R (CD115) (3.0 µg/µl; Bio X Cell, #BE0213) and rat IgG2a isotype control (3.0 µg/µl; Bio X Cell, #BE0089); anti-CD9 (1.0 µg/µl; BD Biosciences, #553758, clone: KMC8) and normal rat IgG control (1.0 µg/µl; BD Biosciences, #553926); anti-FcγRIII (0.5 µg/µl; R&D Systems, #MAB19601) and normal rat IgG control (0.5 µg/µl; R&D Systems, #MAB006). For visualization of antibody distribution in the thalamus, anti-CSF1R Ab was fluorescently labeled using FlexAble 2.0 CoraLite® Plus 647 Antibody Labeling Kit (Proteintech) according to the manufacturer’s instructions. On the injection day, the dummy cannula was replaced with a 33-gauge internal cannula (Plastics One) extending 0.5 mm below the tip of the guide cannula (Plastics One). Unless otherwise stated, 0.5 µl of antibodies or LPS were injected at 100 nl/min using a microinfusion pump (Narishige) per each injection. The internal cannula was left in place for an additional 5 min to diffuse antibodies or reagents properly. PSVue^®^ 550 (1 mM; Molecular Targeting Technologies, P-1005) was stereotactically injected into the thalamus (−1.50 mm AP, +1.50 mm ML, and −3.00 mm DV) using a 10 µl nanofil syringe (WPI) 24 h before perfusion (2 μl of PSVue^®^ 550 per mouse).

### EdU injection and visualization

5-ethynyl-2′-deoxyuridine (Lumiprobe) was prepared following the manufacturer’s instructions. Fifty milligrams per kilogram EdU was administered intraperitoneally every day (day 6–8) or every 3 days (day 7–19). To detect EdU, Click-iT™ EdU Cell Proliferation Kit for Imaging, Alexa Fluor™ 647 dye was used following the manufacturer’s instructions.

### In vivo Axl inhibition

Bemcentinib/R428, a selective inhibitor of Axl (Med Chemical Express), was prepared following the manufacturer’s instructions. Vehicle or 125 mg/kg of bemcentinib was administered intraperitoneally at days 15 and 18.

### DREADD experiments

For neuronal DREADD experiments, AAV injection was conducted as previously described^99^. Briefly, 1 µl of AAV-hSyn-hM3D (Gq) mCherry (3.23 × 10^12^ GC/ml; Addgene, #50474) was stereotactically injected into the thalamus (−1.80 mm AP, +0.50 mm ML, and −3.20 mm DV) of WT mice using a 10 µl nanofil syringe (WPI) at a rate of 200 nl/min immediately before cortical damage (CAI). CNO (10 mg/kg; Cayman, 16882) or vehicle was administered intraperitoneally (i.p.) 30 min before the test session. For microglial DREADD experiments, *Tmem119* ^*CreERT2*^*hM3Dq-mCitrine*^*LSL/LSL*^ mice were injected with 0.5 µl CNO (0.5 mg/mL; Cayman, #16882) or vehicle at a rate of 100 nl/min via an implanted cannula using a microinfusion pump (Narishige) for 3 consecutive days before the analysis.

### Immunohistochemistry

Mice were anesthetized at 2 h after the NOR test and transcardially perfused with ice-cold phosphate-buffered saline (PBS) (pH 7.4) and 4% paraformaldehyde (PFA)/PBS. Brain tissue was post-fixed in 4% PFA overnight, cryoprotected by incubating in 15% and 30% sucrose/PBS, and frozen in the O.C.T. compound. Free-floating sections (30 µm in thickness) were prepared by a Leica cryostat, placed in blocking buffer (5% normal goat serum and 0.1% Triton X-100 in PBS) for 1 h at room temperature, and then incubated with primary antibodies overnight at 4 °C. After washing in PBS, the sections were further incubated with 1:500 dilutions of fluorophore-conjugated secondary antibodies (Thermo Fisher Scientific) for 1 h at room temperature, followed by DAPI staining (10 µg/ml; Thermo Fisher Scientific, D1306) for 10 min. The sections were mounted on slide glasses with ProLong Diamond antifade mounting medium (Thermo Fisher Scientific, P36961) or Fluorescent mounting media (DAKO, S3023). Images were acquired using Zeiss LSM 800 Airyscan confocal microscopes and a ZEN software (Zeiss). Images were collected as *z*-stacks using 10×, 20×, or 63× oil objectives. Laser power, gain, offset, and pinhole size were kept constant within each experiment. The following primary antibodies were used: rabbit polyclonal anti-Iba1 (1:500; WAKO, #019–19741), Alexa Fluor 488-conjugated rabbit monoclonal anti-Iba1 (1:500; abcam, #ab225260), mouse monoclonal anti-Iba1 (1:500; Sigma-Aldrich, #MABN92), Alexa Fluor 647-conjugated mouse monoclonal anti-NeuN (1:500; abcam, #ab190565), rabbit monoclonal anti-NeuN (1:500; abcam, #ab177487), rabbit monoclonal anti-c-Fos (1:500; Cell Signaling Technology, #2250), rabbit monoclonal anti-TSPO (1:100; abcam, #ab109497), rat monoclonal anti-CD68 (1:2000; Novus Biologicals, #NBP2-33337), mouse monoclonal anti-TNF-α (1:100; abcam, #ab1793), rat monoclonal anti-RFP (1:500; Chromotek, #5F8), rabbit monoclonal anti-HA-tag (1:500; Cell Signaling Technology, #3724), armenian hamster monoclonal anti-CD31/PECAM1 (1:100; DSHB, #2H8-s), rabbit polyclonal CD9 (1:100; Proteintech, #20597-1-AP), rat monoclonal CD9 (1:100; BD Biosciences, #553758), rabbit polyclonal anti-FcγRIII (1:100; abcam, #ab203883), rabbit polyclonal anti-p-Syk (1:200; Cell Signaling, #2710 T), mouse monoclonal anti-PSD95 (1:500; Thermo Fisher Scientific, #MA1-046), chicken monoclonal anti-Tmem119 (1:500; Synaptic Systems, #400009), sheep polyclonal anti-Trem2 (1:100; R & D, #AF1729), rabbit polyclonal anti-Claudin5 (1:100, Thermo Fisher Scientific, #34-1600), rabbit polyclonal anti-Ifitm3 (1:100; Proteintech, #11714-1-AP), guinea pig monoclonal anti-GAD67 (1:100; Synaptic systems, #198308), rat monoclonal anti-Vcam1 (1:100; BD Biosciences, #550547), rabbit polyclonal anti-VGLUT1 (1:100; Synaptic systems, #135303), Alexa Fluor 488-conjugated rat monoclonal anti-CD206 (1:100; Biolegend, #141709), Alexa Fluor 647-conjugated rat monoclonal anti-CD169 (1:100; Biolegend, #142407), goat polyclonal anti-Axl (1:100; R & D, #AF854), APC rat monoclonal anti-Ly-6C (1:100; Biolegend, #128016), and rabbit polyclonal anti-VGLUT2 (1:100; Synaptic systems, #135403).

### Image analysis

Image analysis was performed using Fiji/ImageJ software as previously described^99–102^. The number of z-stacked images (7–15 images) and the image intervals were the same across the groups being compared. Maximum intensity projection was used to generate 2D-projected images for analysis unless otherwise noted. For microglial synapse engulfment analysis, 3D surface analysis was performed with Imaris software (Bitplane) using z-stacked images.

#### (1) Definition of regions of interest (ROIs)

- Thalamus; for each mouse, 3-5 ROIs (600 × 600 μm or 1200 × 1200 μm) were sampled from comparable anatomical coordinates (Bregma: −1.50 to −1.80 mm).
- Hippocampus (CA1, CA3, and dentate gyrus); for each mouse, 3 ROIs (600 × 600 μm) were sampled from comparable anatomical coordinates (Bregma: −1.50 to −1.80 mm).
- Corpus callosum (defined as the midline white matter band); ROIs (600 × 600  μm) were sampled, avoiding cannula/injection boundaries.

The ROI sizes and numbers per mouse were the same across the groups being compared.

#### (2) Background subtraction and thresholding

All channels were first processed using rolling-ball background subtraction (radius = 50 pixels) and median filter (radius = 1 pixel). Thresholding was performed by the Otsu method and applied uniformly across samples within each experiment. Thresholds were also verified by visual inspection to avoid loss of the true signal.

#### (3) Quantification and normalization for summary bar graphs

For all immunohistochemistry-based quantifications, raw measurements were first obtained per ROI. When multiple ROIs/fields were analyzed for a given animal, ROI-level values were averaged to yield a single value per mouse (biological replicate). For bar graphs displayed as “relative expression,” each mouse value was normalized to the mean of the corresponding control group processed in parallel within the same experiment (control group set to 1). For bar graphs displayed as “% of control,” each mouse value was normalized to the mean of the corresponding control group processed in parallel within the same experiment (control group set to 100%). Specifically, for each mouse:

$$\%{{\rm{control}}}=\left(\frac{{{{\rm{Mean\; Value}}}}_{{{\rm{treatment\; group}}}}}{{{{\rm{Mean\; Value}}}}_{{{\rm{control\; group}}}}}\right)\times 100$$

Metrics that are intrinsically expressed as percentages or ratios (e.g., extravascular IgG coverage area/total IgG coverage area) were calculated directly as described and were not additionally normalized unless otherwise stated.

#### (3-1) Iba1⁺ and NeuN⁺ cells

Iba1⁺ cell quantification. Iba1 binary masks were generated as described above in (2). Iba1^+^ cells were identified using the “analyze particles” function with the following criteria: size filter = 20–300 μm^2^ and circularity = 0.2–1.0 (to include both ramified and amoeboid microglia). Iba1⁺ cell count was presented as “Iba1^+^ cells per mm^2^” or “total number of Iba1^+^ cells” per ROI, with data averaged across 5 ROIs per mouse.

NeuN^+^ cell quantification. NeuN binary masks were generated as described above in (2). Watershed separation was applied to distinguish the attached NeuN signals. NeuN^+^ nuclei were identified using the “analyze particles” function using the following criteria: size filter = 40–300 μm^2^ and circularity = 0.5–1.0. NeuN⁺ cell count was presented as “NeuN⁺ cells per mm^2^” per ROI, with data averaged across 5 ROIs

#### (3-2) Relative Iba1 expression for correlation

Mean Iba1 fluorescence intensity was measured within each ROI. Data were averaged from 5 ROIs per mouse to obtain a single value per biological replicate. For the “relative Iba1 expression” plot, values for each mouse were normalized to the mean value of the corresponding contralateral control group (control = 1.0) evaluated in parallel.

#### (3-3) Neuronal c-Fos expression

NeuN binary masks were generated as described above in (3-1). c-Fos immunofluorescent intensity was measured within each NeuN^+^ mask. Moments thresholding was applied, and nuclei above thresholds were classified as c-Fos positive. Background was calculated from a cell-free area of each image. For each ROI, the number of c-Fos^+^ neurons was calculated. Data were averaged from 5 ROIs per mouse to obtain a single value per biological replicate. The count was presented as “c-Fos^+^ NeuN^+^ neurons cells/mm^2^” and “relative c-Fos expression” was calculated as the values normalized to one of the conditions, which control group was set to 1.0

#### (3-4) TSPO immunofluorescence in Iba1^+^ cells

Iba1 binary masks were generated as described above in (3-1). Mean TSPO immunofluorescence intensity was measured within each Iba1^+^ mask. Data from 5 ROIs per mouse were averaged to obtain a single value per mouse. For “relative TSPO expression” plots, each mouse value was normalized to the mean of the corresponding control group processed in parallel (control = 1.0).

#### (3-5) Microglial synapse engulfment

Confocal z-stacks were acquired using a 63× oil-immersion objective (NA 1.4) on a Zeiss LSM800 in the thalamus, with a z-step of 0.3 μm. Stacked images were processed and analyzed in Imaris (Oxford Instruments) using the *surfaces* and *mask* modules. Microglial cell bodies and processes were segmented from the Iba1 channel using the *surfaces* module (background subtraction, local contrast; identical intensity and size thresholds were applied to all samples within a given experiment). CD68^+^ lysosomes were then segmented within the Iba1 surfaces to generate CD68^+^ volumes per microglial cell. To quantify engulfed synaptic material, PSD95^+^ puncta was first segmented independently using *surfaces* (expected diameter 0.3–0.4 μm; fixed intensity and voxel thresholds across groups). A new channel representing “engulfed synapses” was created by masking the PSD95 signal with the CD68⁺ surfaces (*mask* function in Imaris: “keep voxels inside CD68 surface, set outside to 0”), such that only synaptic puncta fully contained within CD68⁺ lysosomes were retained, analogous to previous work. 3D surfaces were then rendered on this masked channel, and for each Iba1^+^ cell, the number and total volume of engulfed PSD95^+^ puncta were quantified. Engulfment was expressed as “number of engulfed synaptic puncta per microglial cell.”

#### (3-6) Synapse quantification

For synapse density measurements, confocal z-stacks (63×, z-step 0.3 μm) were acquired in the thalamic using identical acquisition settings across animals. VGLUT1 or VGLUT2 (presynaptic markers) and PSD95 (postsynaptic marker) were analyzed. VGLUT1/VGLUT2^+^ and PSD95^+^ puncta were detected independently as “spots” with an expected diameter of 0.3 μm (XY), and a local background subtraction; the same intensity threshold and size filter were applied to all images within an experiment. For each channel, obvious noise and out-of-focus structures were excluded by a lower size cutoff (–0.1 μm^2^). To define putative excitatory synapses, we considered a presynaptic and a postsynaptic punctum to be colocalized when the center-to-center distance between a VGLUT1/VGLUT2 spot and a PSD95 spot was <0.5 μm in 3D space. This distance threshold corresponds approximately to the size of a single synaptic contact and has been used to estimate synapse density in similar studies. Colocalized spots were identified using ImageJ. For each ROI (typically 100 × 100 μm in the thalamus), we quantified: number of VGLUT1^+^/VGLUT2^+^–PSD95^+^ colocalized spots (synapses). Synapse density was expressed as the number of colocalized puncta per ROI. Five ROIs per mouse and *n* = 4–10 mice per group were analyzed. The same thresholds and analysis parameters were applied within each experiment.

#### (3-7) CD9^hi^ Iba1^+^ cells

Iba1 and CD9 binary masks were generated, respectively, using the method described above. Iba1^+^ cells were identified using the “analyze particles” function with the following criteria: size filter = 20–300 μm^2^ and circularity = 0.2–1.0 (to include both ramified and amoeboid microglia). CD9^+^ cells were identified using the “analyze particles” function with the following criteria: size filter = 10–150 μm^2^. Iba1⁺CD9^hi^ cells were defined when Iba1 and CD9 masks overlapped by ≥5% of the Iba1-labeled area within the same cell boundary. Overlap was computed using the “image calculator (AND)” function. The count was presented as “CD9^hi^ Iba1^+^ cells per mm^2^,” with data averaged across 5 ROIs per mouse.

#### (3-8) Spatial mapping of microglial states

For spatial pattern quantification, immunostained thalamic sections were analyzed from three independent mice. Atlas-matched thalamic subregions were delineated. Within each subregion, Iba1^+^ microglia were identified, and marker expression was quantified to classify microglial states using predefined criteria (e.g., MG#4 as CD9^hi^ microglia; MG#6 as IFITM3^hi^ microglia). For each subregion in each mouse, we calculated the fraction of Iba1^+^ microglia assigned to each state, and defined the “dominant” state as the state with the highest fraction in that subregion. These dominant states were consistent across 3 mice.

#### (3-9) VGLUT2^+^ or GAD67^+^ neuronal termini

To measure the density of excitatory or inhibitory inputs, confocal z-stack images (63×, z-step 0.3 μm) were acquired in the thalamus using identical acquisition settings across animals. VGLUT2 (excitatory input) and GAD67 (inhibitory input) were analyzed. VGLUT2^+^ and GAD67^+^ puncta were detected as “spots” with an expected diameter of 0.3 μm, respectively, and local background subtraction was performed. The same intensity threshold and size filter were applied to all images within an experiment. In each channel, obvious noise and out-of-focus structures were excluded by a lower size cutoff (−0.1 μm^2^). The number of VGLUT2^+^ or GAD67^+^ puncta was quantified for each ROI (typically 100 × 100 μm). Data from 5 ROIs per mouse were averaged to obtain a single value, and the values from four individual mice per group were shown.

#### (3-10) IgG extravasation, Claudin5 expression, and Vcam1 expression

For IgG extravasation, the particle sizes ranging from 1 to 10 μm^2^ were quantified. Values for each sample were reported as area fraction per ROI. For Claudin5 and Vcam1 expression, a vascular binary mask was created using CD31. Then, the average immunofluorescent intensities of Claudin5 or Vcam1 signals within the vascular mask were calculated. Data from 5 ROIs per mouse were averaged to obtain a single value, and thalamic data were normalized to the HPC mean from the same mouse.

### Single-cell RNA sequencing (scRNA-seq)

#### Sample preparation and sequencing

Thalamic and hippocampus (HPC) tissues from the injured hemispheres were micro-dissected, and single cell suspensions were obtained following the manufacturer’s protocol and as described previously^103^. Briefly, tissue was transferred into 2 ml of pre-chilled homogenization buffer [15 mM HEPES buffer, 1 mg/mL DNase I (Roche), and Protector RNase inhibitor at 1:200 (Roche)]. Tissues were carefully homogenized 10–12 times with Dounce homogenizers, filtered with 70 μm cell strainers, and centrifuged at 1250 × *g* for 2 min to pellet cells. Then, debris was removed using Debris Remove Solution (Miltenyi). Equal numbers of cells from 4 individual mice were pooled together for the thalamus and the HPC, respectively. Samples were Fc-blocked with anti-mouse CD16/32 antibody (1:100, Biolegend, clone: 93), and stained with 7-AAD (ThermoFisher), FITC-conjugated anti-mouse CD11b (1:100; Biolegend, clone: M1/70, #101206), and APC-conjugated anti-mouse CD45 (1:500; Biolegend, clone: 30-F11, #103112). Then, live CD45^+^ cells were sorted using the FACS Aria II at the UAB Comprehensive Flow Cytometry Core (CFCC). scRNA-seq libraries were constructed on the 10× Genomics platform using the Single Cell 3′ protocol (v.3.1, 10× Genomics). The libraries were sequenced using NovaSeq6000 at the UAB Genomics Core facility.

#### Data analysis

Raw scRNA-seq data were processed using the Cell Ranger software (v.6.0.2, 10× Genomics) for sample demultiplexing, barcode processing, and single-cell counting. Reads were aligned to the GRCm38 (mm10) mus musculus reference genome. The outputs from the Cell Ranger software analysis were then imported into the Seurat package (v.4.0) in R (v.4.1.1), and the subsequent analysis was conducted with Seurat. For quality control, cells containing more than 2% of mitochondrial counts and extreme unique feature counts (over 7500 or less than 200) were removed. Data were normalized with a scaling factor of 10,000. The data from multiple libraries were then combined using FindIntegrationAnchors() and IntegrateData() functions. Principal component analysis (PCA) was performed using the combined data. A scree plot was generated using the ElbowPlot() function to select the principal components (PCs) by localizing the last PC before the explained variance reaches a plateau. Selected PCs were used to calculate nearest-neighbor distances and perform Louvain clustering with FindNeighbors() and FindClusters() functions. Then, uniform manifold approximation and projection (UMAP) was used to visualize clusters. Cell types were initially annotated using SingleR^104^, and then refined based on the expression of known marker genes in the genes significantly enriched for each cluster that was obtained using the FindAllMarkers() function. The Wilcoxon rank-sum test was used to identify genes differentially expressed between injury and sham groups per cluster. Among the 19 identified clusters, six clusters were annotated as microglia and two clusters were annotated as macrophages for further analysis.

Gene set enrichment analysis (GSEA) was performed for the microglia cluster data using the gsea() function of Clusterprofiler (v. 4.8.3)^105,106^. To visualize the GSEA result, the Dotplot() and ggplot() functions were employed. Gene sets for homeostatic microglia (HM), DAM, and IRM were created according to previous studies^107–110^. To assess similarity between the MG#4 cluster and previously reported DAM and DAM-related states, we retrieved published differentially expressed gene (DEG) lists from snRNA-seq studies. We quantified similarity between MG#4 and each published gene set using the Jaccard index, defined as the ratio of shared genes to the total number of unique genes across both sets:

$$J\left(A,\,B\right)=\frac{\left|A\cap B\right|}{\left|A\cup B\right|}$$

where *A* represents MG#4 DEGs and *B* represents the published DEG set. We also performed bootstrap subsampling to assess robustness and minimize bias introduced by differences in gene set sizes. In each of 1000 iterations, 80% of genes were randomly sampled without replacement from both gene sets, and the Jaccard index was recalculated. The resulting distributions were visualized using violin plots, with observed Jaccard values from full gene sets overlaid for comparison.

Pseudo-time analysis was performed using Monocle 3^111–113^ on the six microglial clusters. These clusters were first randomly downsampled to 1000 cells per cluster by the subset() function, and microglia cluster 3 was set as the pseudo-time root cluster based on the expression profile matching that of known homeostatic microglia. Pseudo-time was then calculated and plotted for visualization. A list of genes identified as important in calculating pseudo-time was also generated using Monocle 3.

To compare cluster compositions between the thalamus and HPC or the anti-CD9 Ab group thalamus and control Ab group, cell number and normalized ratio in each cluster were counted. The result was visualized using the ggplot() function.

Individual gene expression patterns across cells were visualized by the FeaturePlot() function. The FindMarkers() function was used to identify the DEGs in the MG#4 cluster between the thalamus and the HPC, and MG#4 clusters between the anti-CD9 Ab group thalamus and the control Ab group thalamus. The results were visualized as a volcano plot using the ggplot() function. The single-cell heatmap was generated using the DoHeatmap() function, while the pseudo-bulk heatmap was created by first calculating the average gene expression using the AggregateExpression() function, and then plotting the data using the ggplot() function. To minimize batch effects in the data obtained from experiments using antibody and in the comparison experiments between the thalamus and the hippocampus, the RunHarmony() function was used. The UMAP plot generated using the dataset created with the RunHarmony() function was randomly downsampled to 3000 cells per condition by the subset() function.

### Single-nucleus RNA-sequencing (snRNA-seq)

#### Sample preparation and sequencing

Thalamic tissues from the CAI or sham-operated hemispheres were micro-dissected and frozen until nuclei preparation using a previously reported method for other brain regions^114^. Nuclei were harvested following the manufacturer’s protocol. Briefly, frozen tissue was transferred into 2 ml of pre-chilled homogenization buffer [320 mM sucrose, 0.1% NP40, 0.1 mM EDTA, 5 mM CaCl_2_, 3 mM Mg(Ac)_2_, 10 mM Tris-HCl (pH 7.8), 167 µM β-mercaptoethanol, 1× protease inhibitor (Roche), and Protector RNase inhibitor at 1:200 (Millipore)]. Tissues were carefully homogenized with Dounce homogenizers, filtered with 70 μm cell strainers, and centrifuged at 100 × *g* for 1 min to pellet nuclei. Equal numbers of nuclei from four individual mice were pooled, stained with propidium iodide, and sorted using FACS Aria II at the UAB CFCC. Then, snRNA-seq libraries were constructed on the 10× Genomics platform using the Single Cell 3′ protocol (v.3.1, 10× Genomics). The libraries were sequenced using NovaSeq6000 at the Johns Hopkins University Genetic Resources Core Facility (GRCF).

#### Data analysis

Raw snRNA-seq data were processed using the Cell Ranger software (v.6.0.2, 10× Genomics) for sample demultiplexing, barcode processing, and single-cell counting. Reads were aligned to the GRCm38 (mm10) mus musculus reference genome. The outputs from the Cell Ranger software analysis were then imported into the Seurat package (v.4.0) in R (v.4.1.1), and the subsequent analysis was conducted with Seurat. For quality control, nuclei with mitochondrial content more than 2% and extreme unique feature counts (more than 7500 and less than 200) were removed. Data were normalized with a scaling factor of 10,000. The data from multiple libraries were then combined using FindIntegrationAnchors() and IntegrateData() functions. PCA was performed using the combined data. A scree plot was generated using the ElbowPlot() function to select the PCs by localizing the last PC before the explained variance reaches a plateau. Selected PCs were used to calculate nearest-neighbor distances and perform Louvain clustering with FindNeighbors() and FindClusters() functions. Then, UMAP was used to visualize clusters. Cell types were annotated based on the expression of known marker genes in the genes significantly enriched for each cluster. The Wilcoxon rank-sum test was used to identify genes differentially expressed between injury and sham groups per each cluster. Differential gene expression analysis was performed across all neuronal clusters, comparing Injury and Sham conditions using the FindMarkers() function. Genes with |log2 fold change| ≥ 0.25 and adjusted *P* < 0.05 were considered differentially expressed. Functional enrichment analysis was conducted for clusters containing more than 10 significantly upregulated or downregulated genes using clusterProfiler. Gene Ontology (GO) biological process enrichment was performed with enrichGO() using Bonferroni correction. All significant pathways were retained, and the top 20 pathways ranked by enrichment significance were compared across clusters.

### Flow cytometry

Single-cell suspensions from mouse thalamus and/or hippocampus were prepared using a Dounce homogenizer followed by debris removal using Debris Removal Solution (Miltenyi Biotec). For peripheral blood analyses, red blood cells were lysed using 1× RBC lysis buffer (Thermo Fisher Scientific/eBioscience). Cells were washed with FACS buffer and stained with LIVE/DEAD™ Fixable Aqua Dead Cell Stain Kit for 405 nm excitation (Thermo Fisher Scientific), followed by Fc-block with anti-CD16/32 (1:100, Biolegend, clone: 93, #101320) for 15 min on ice. Then, cells were stained with the following antibodies for 20 min at 4 °C protected from light: BV785-conjugated anti-mouse CD11b (1:100, Biolegend, clone: M1/70, #101243), APC/Cyanine7-conjugated anti-mouse CD45 (1:100, Biolegend, clone: S18009F, #157204), APC-conjugated anti-mouse Ly6C (1:100, Biolegend, clone: HK1.4, #128016), BV421-conjugated anti-mouse Tmem119 (1:300, Invitrogen, clone: V3RT1Gosz, #404-6119-82), PerCP/Cyanine5.5-conjugated anti-mouse CD9 (1:100, Biolegend, clone: MZ3, #124818), PE-conjugated anti-mouse Osteopontin/Spp1 (1:100, R&D Systems, polyclonal, #IC808P), CoraLite®594-conjugated anti-mouse IFITM3 (1:100, Proteintech, polyclonal, #CL594-11714), and PE-Cy-7-conjugated anti-mouse cleaved caspase-3 (1:100, Cell signaling, clone: D3E9, #64772). To minimize potential epitope masking after in vivo anti-CD9 administration, CD9 was detected using an antibody clone distinct from the clone used for in vivo experiments (MZ3 for flow cytometry vs. KMC8 for in vivo experiments). For intracellular staining (e.g., IFITM3, Spp1, and cleaved caspase-3), cells were fixed and permeabilized using True-Nuclear™ Transcription Factor Buffer Set (Biolegend) and stained with antibodies in permeabilization buffer for 30 min at 4 °C. Data were acquired on a BD LSRFortessa flow cytometer with compensation calculated from single-stained controls prepared using UltraComp eBeads™ Compensation Beads (Thermo Fisher). Fluorescence-minus-one controls were used to define positivity thresholds. Flow cytometry data were analyzed using FlowJo software (v10, BD Biosciences). Gates were applied consistently across experiments, and biological replicates (*n*) represent individual mice unless otherwise stated. Myeloid cells were identified as CD11b⁺CD45⁺ cells. Microglia were defined as CD11b^+^CD45^+^Ly6C^−^ cells, and infiltrating monocytes/macrophages were identified as CD11b^+^CD45^+^Ly6C^+^ cells. For microglial subset analyses, thalamic/hippocampal microglia were further stratified based on Tmem119, CD9, and IFITM3 expression as indicated in the corresponding figures. MG#4 was defined as CD9^hi^ Tmem119^lo^ microglia; MG#2/3 as Tmem119^hi^ CD9^lo^ microglia; and within the Tmem119^lo^ CD9^lo^ fraction, MG#6 as IFITM3⁺ cells and MG#1 as IFITM3⁻ cells.

### Behavioral assays

#### Novel object recognition (NOR) test

The NOR test evaluates the tendency of rodents to discriminate between new and familiar objects and is used to assess recognition memory in mice^115^. The test mice were individually habituated for 10 min in an open field box (45 × 45 cm; Harvard Apparatus) for 2 consecutive days. The next day, the test mice were exposed to the familiar open field with two identical objects placed at an equal distance in a symmetrical position for 10 min. Twenty-four hours later, the test mice were allowed to explore the same open field with the same object (familiar object) and a new object with a different shape (novel object). The exploratory behaviors of the test mice for 10 min were recorded and analyzed using EthoVision XT17 (Noldus). Exploration of the object was counted when the animal’s nose entered a 2 cm radius around the object. The discrimination index was calculated as ([time spent at the novel object] – [time spent at the familiar object])/([time spent at the novel object] + [time spent at the familiar object]).

#### Y-maze test

The Y-maze was performed and completed 21 days post-CAI. The Y-maze apparatus was composed of 2 arms connected to a runway (home arm), each 45 × 15 × 15 cm^31^. The arms contained different visual cues, consisting of black and white symbols/shapes on paper. The maze was positioned on the ground, and the mice were tracked by an overhead camera. During training, the animals were placed in the home arm with access to only one arm for 5 min. Following a 1 h intermission, mice were placed back into the Y-maze for 5 min with access to the training and novel arms. The time spent in the novel and the training arms was analyzed using EthoVision XT17 (Noldus). The % of novelty arm preference was calculated as ([time spent at the novel arm]/([time spent at the novel arm] + [time spent at the familiar arm]) × 100).

Raw data for behavioral assays are available in the Supplementary Data S7.

### Statistical analysis

Data were analyzed with Student’s *t* test, Dunnett’s test, Tukey–Kramer’s test, and ANOVA using Microsoft Excel and GraphPad Prism 8 (GraphPad Software) unless stated otherwise. Post hoc analyses for one-way ANOVA were performed using Dunnett’s test method. Significant differences were considered at *p* < 0.05. Further details of the statistical analysis for each figure are available in the Supplementary Data S8.

### Reporting summary

Further information on research design is available in the Nature Portfolio Reporting Summary linked to this article.

## Supplementary information

Supplementary Information

Supplementary Data

Reporting Summary

Transparent Peer Review File

## Source data

Source Data

## Data Availability

Source data are provided with this paper. scRNA-seq data are available at the NCBI Gene Expression Omnibus under accession numbers GSE200264, GSE260805, and GSE275711. Source data are provided with this paper.
