# Supplementary material for: FcγR- and CD9-dependent synapse-engulfing microglia in the thalamus drive cognitive impairment following cortical brain damage in mice
Source: Nat Commun. 2026 Jul 9;17:8514. doi: 10.1038/s41467-026-74904-1 (PMC13484472; doi:10.1038/s41467-026-74904-1)
Supplement: Supplementary file 1 [file 41467_2026_74904_MOESM1_ESM.pdf]

**Supplementary Information**

# FcγR- and CD9-dependent synapse-engulfing microglia in the thalamus drive cognitive impairment following cortical brain damage in mice

Ken Matoba[1], Takahiro Kochi[1,2], Oluwaseun Fatoba[1], Md Sorwer Alam Parvez[1], Inssaf Berkiks[1], Yassin R Mreyoud[1], Harrison Strong[1], Jana H. Badrani[1], Hency Patel[1], Yoshiko Nagaoka-Kamata[3], Masakazu Kamata[4,5], Hiroshi Tsujioka[6], Toshihide Yamashita[6], David K. Crossman[7], Minae Niwa[1,8,9], and Shin-ichi Kano[1,8]*

[1]Department of Psychiatry and Behavioral Neurobiology, University of Alabama at Birmingham Heersink School of Medicine, Birmingham, AL, 35233, USA
[2]Department of Mental Disorder Research, National Institute of Neuroscience, National Center of Neurology and Psychiatry, Tokyo, 187-0031, Japan
[3]Department of Pathology, University of Alabama at Birmingham Heersink School of Medicine, Birmingham, AL, 35233, USA
[4]Department of Microbiology, University of Alabama at Birmingham Heersink School of Medicine, Birmingham, AL, 35233, USA
[5]O'Neal Comprehensive Cancer Center, University of Alabama at Birmingham Heersink School of Medicine, Birmingham, AL, 35233, USA
[6]Department of Molecular Neuroscience, Graduate School of Medicine, The University of Osaka, Osaka, 565-0871, Japan
[7]Department of Genetics, University of Alabama at Birmingham Heersink School of Medicine, Birmingham, AL, 35233, USA
[8]Department of Neurobiology, University of Alabama at Birmingham Heersink School of Medicine, Birmingham, AL, 35233, USA
[9]Department of Biomedical Engineering, University of Alabama at Birmingham School of Engineering, Birmingham, AL, 35294, USA

*Corresponding should be addressed to S.K. (sikano@uabmc.edu)

# Supplementary Fig. 1

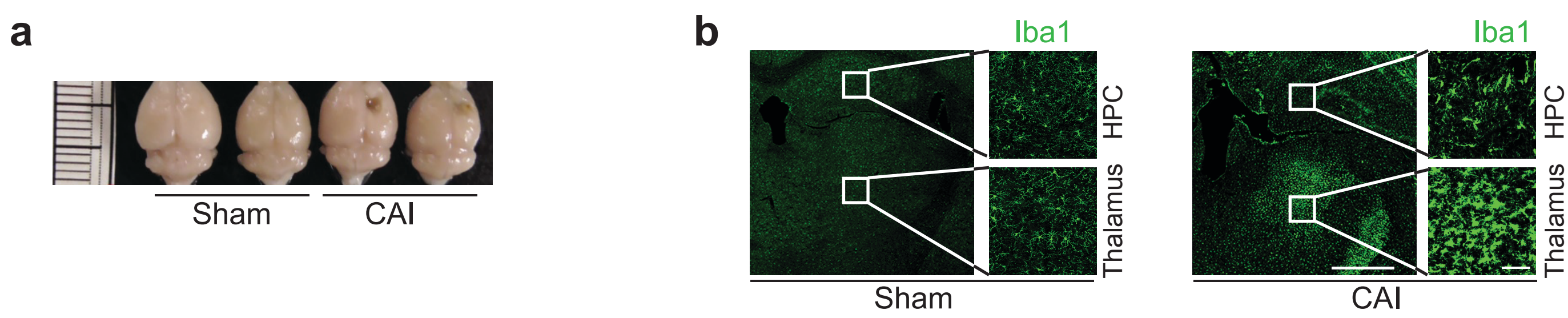


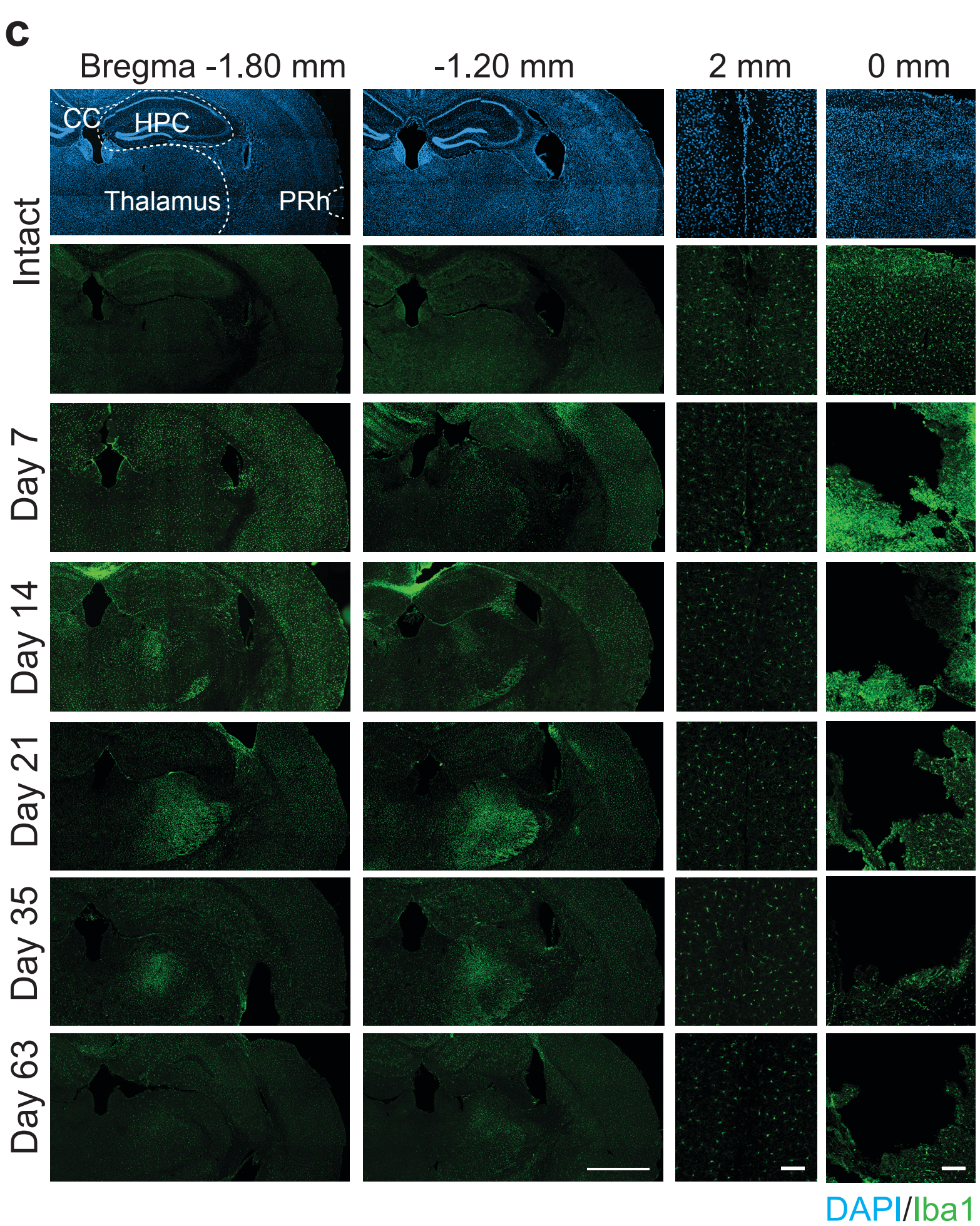


d

| | mPFC | CC | PRh | HPC | Thalamus AD/AV | MD/MV | CM | VM | Po/VL | VPL | VPM |
|---|---|---|---|---|---|---|---|---|---|---|---|
| Intact | - | - | - | - | - | - | - | - | - | - | - |
| Day 7 | - | + | - | ± | - | - | - | - | ± | - | - |
| Day 14 | - | +++ | - | ± | - | - | - | - | + | + | - |
| Day 21 | - | ++ | - | + | + | + | + | + | ++ | + | + |
| Day 35 | - | + | - | ± | + | + | + | + | ++ | + | + |
| Day 63 | - | ± | - | ± | - | - | - | - | + | + | + |

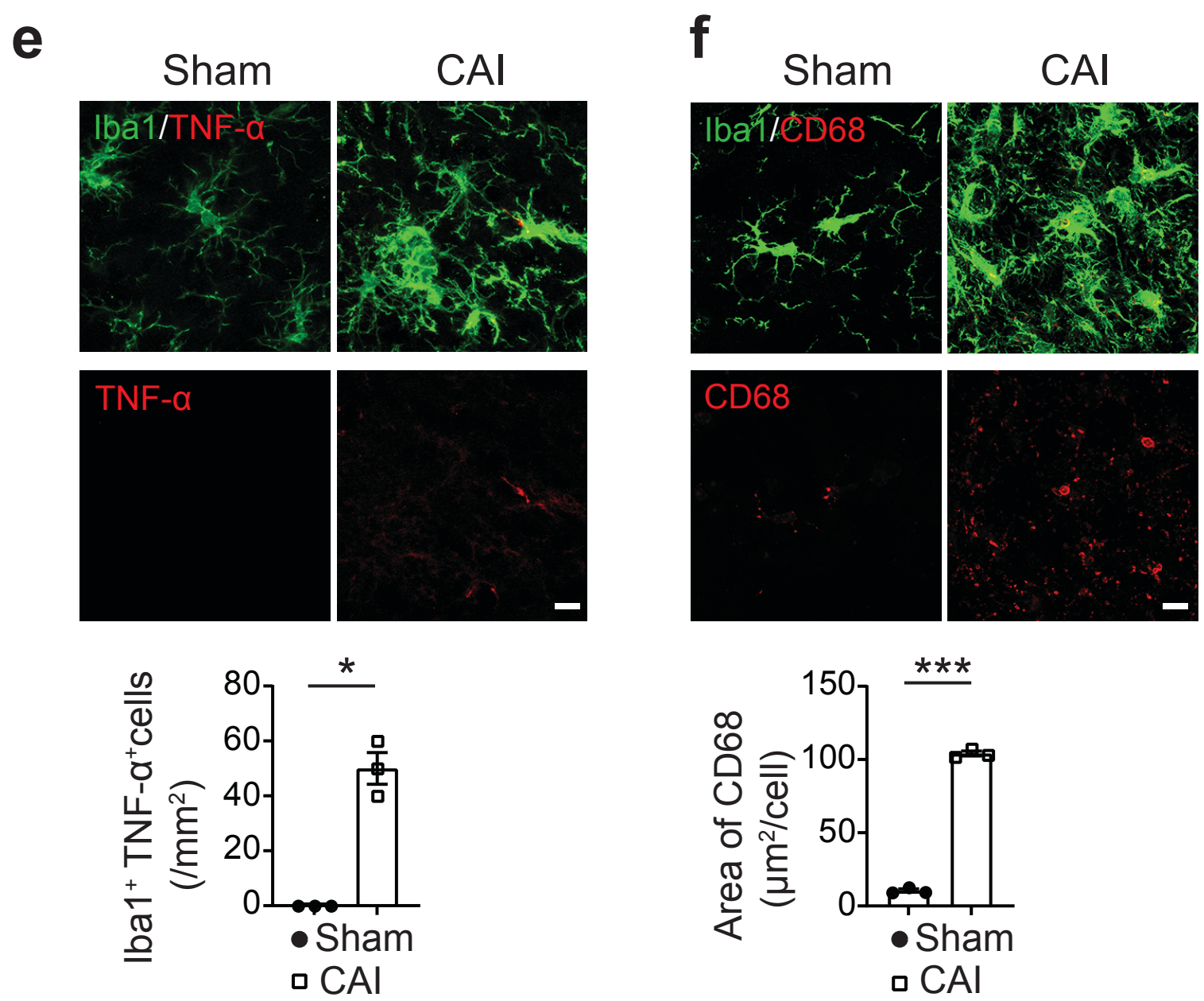


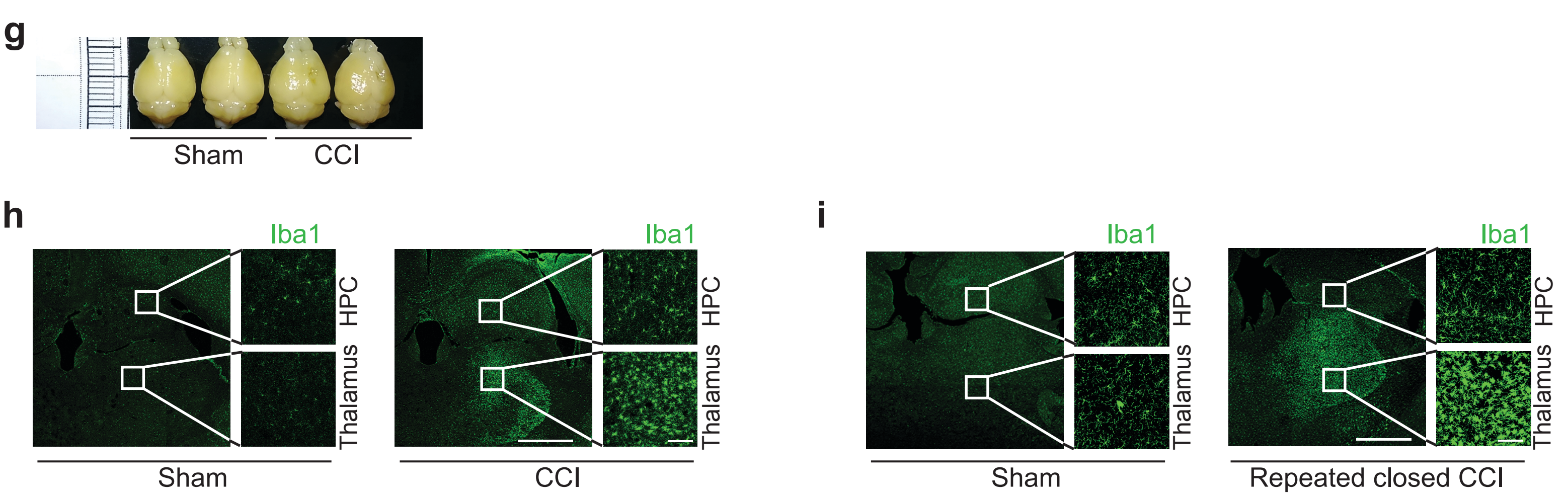

**Supplementary Fig. 1. Cortical brain damage induces delayed microglial changes.**

**a**, Representative images of the gross brain morphology 21 days after the unilateral CAI and sham surgery.

**b**, Representative images of Iba1$^+$ cells 21 days after CAI and sham surgery. Scale bars: 1 mm (in low-magnification image) and 100 µm (in high-magnification image). HPC, hippocampus.

**c**, Representative images of Iba1$^+$ cells in the intact brain and the brains on day 7, 14, 21, 35, and 63 post-CAI. Scale bar: 500 µm. CC, corpus callosum; PRh, perirhinal cortex.

**d**, Summary of delayed Iba1$^+$ cells reactive changes across various brain regions. The severity of Iba1$^+$ cells activation is indicated as follows: -, no activation; ±, faint activation; +, mild activation; ++, moderate activation; and +++, massive activation. mPFC, medial prefrontal cortex; CC, corpus callosum; PRh, perirhinal cortex; HPC, hippocampus; AD, anterodorsal nucleus; AV, anteroventral nucleus; MD, mediodorsal nucleus; MV, medioventral nucleus; CM, central medial nucleus; VM, ventral medial nucleus; VL, ventral lateral nucleus; Po, posterior nucleus; VPL, ventral posterolateral nucleus; VPM, ventral posteromedial nucleus.

**e**, Representative images (top) and quantification (bottom) of TNF-α expression in Iba1$^+$ cells in the thalamus 21 days after CAI and sham surgery. Scale bar, 10 µm. Quantification data show the density of TNF-α expressing Iba1$^+$ cells (n = 3 mice for sham; n = 3 mice for CAI).

**f**, Representative images (top) and quantification (bottom) of CD68 expression in Iba1$^+$ cells in the thalamus 21 days after CAI and sham surgery. Scale bar, 10 µm. Quantification data show average CD68$^+$ area in Iba1$^+$ cells (n = 3 mice for sham; n = 3 mice for CAI).

**g**, Representative images of the gross brain morphology 21 days after controlled cortical impact (CCI) and sham surgery.

**h**, Representative images of Iba1$^+$ cells 21 days after open CCI and sham surgery. Scale bars: 1 mm (in low-magnification image) and 100 µm (in high-magnification image).

**i**, Representative images of Iba1$^+$ cells 21 days after repeated closed CCI and sham surgery. Scale bars: 1 mm (in low-magnification image) and 100 µm (in high-magnification image).

In bar graphs, data are shown as the mean ± s.e.m, and each dot represents an individual animal. n indicates individual mice. Data were collected from a single cohort. *$p < 0.05$, ***$p < 0.001$ (unpaired two-tailed *t*-test). Further details of the statistical analysis are available in the **Supplementary Data S8**. Source data are provided as a Source Data file.

# Supplementary Fig. 2

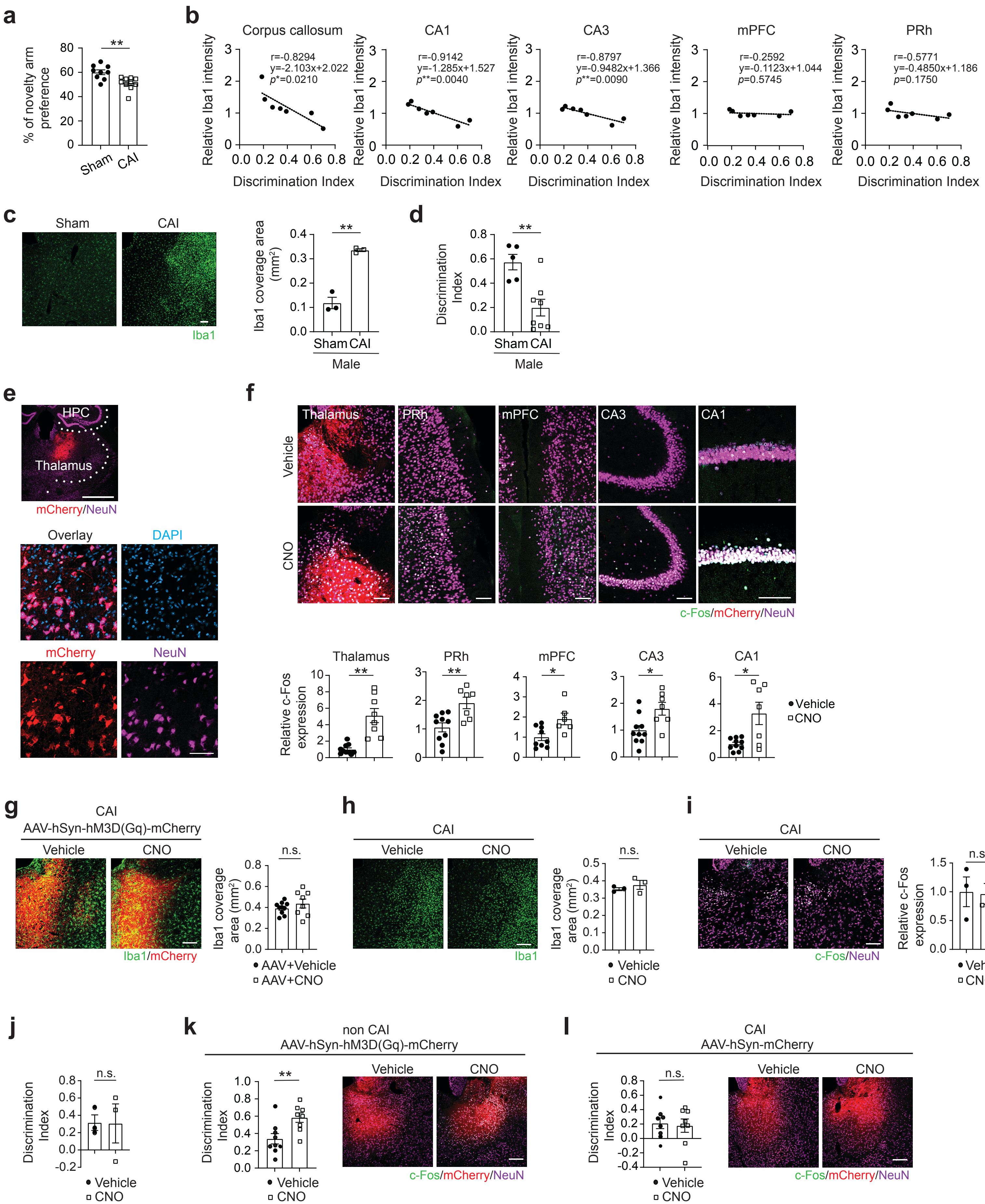

**Supplementary Fig. 2. Chemogenetic activation of thalamic neurons ameliorates the NOR deficits in mice after CAI.**

**a,** Y-maze test data. Percentages of time spent in the novel arm are shown for CAI and sham group (n = 9 mice per group).

**b**, Correlation plots of the NOR discrimination indices and Iba1 intensities in the corpus callosum, HPC, mPFC, and PRh 21 days after CAI and sham surgery (n = 7 mice). Pearson's correlation coefficient (r) and two-tailed *p* values are shown in the plots.

**c**, Representative images (left) and quantification (right) of $Iba1^+$ cells in the thalamus 21 days after CAI and sham surgery. Scale bar: 100 µm. Quantification data show Iba1 coverage area (n = 3 mice per group).

**d**, NOR test data. Discrimination index is shown for sham (n = 5 mice) and CAI (n = 8 mice) group.

**e**, Representative images showing the expression of AAV-derived mCherry in thalamic neurons 21 days after injection. Scale bars, 1 mm (left picture) and 100 µm (right picture).

**f**, Representative images (top) and quantification (bottom) of neuronal c-Fos expression in the thalamus, PRh, mPFC, and HPC (CA1, CA3) 21 days after CAI and vehicle or CNO injection. Scale bars, 100 µm. Quantification data show neuronal c-Fos expression in the CNO-treated group (n = 8 mice) relative to the vehicle-treated mice (n = 11 mice).

**g**, Representative images (left) and quantification (right) of $Iba1^+$ cells in AAV-hSyn-hM3D(Gq)-mCherry-injected areas (indicated by mCherry expression) in the thalamus of mice with CNO or vehicle administration. Scale bar, 100 µm. Quantification data show Iba1 coverage area (n = 10) mice for the vehicle group; n = 8 mice for the CNO group).

**h,** Representative images (left) and quantification (right) of $Iba1^+$ cells in the thalamus of injured mice without AAV injection after CNO or vehicle administration. Scale bar, 100 µm. Quantification of Iba1 coverage area is shown on the right (n = 3 mice per group).

i, Representative images (left) and quantification (right) of neuronal c-Fos expression in the thalamus of injured mice without AAV injection after CNO or vehicle administration. Scale bar, 100 µm. Quantification data show neuronal c-Fos expression in the CNO group relative to the Veh group (n = 3 mice per group).

**j**, Discrimination index in the NOR test for vehicle and CNO-treated injured mice without AAV injection (n = 3 mice per group).

**k**, Left, NOR test discrimination index for Veh- and CNO-treated uninjured mice with AAV-hSyn-hM3D(Gq)-mCherry injection into the thalamus (n = 8, 9 mice per group). Right, representative images of neuronal c-Fos expression after NOR test. Scale bar, 100 µm.
**l**, Left, representative images of neuronal c-Fos expression in the thalamus 21 days after AAV-hSyn- -mCherry, CAI, and vehicle or CNO injection. Scale bar, 100 µm. Right, NOR discrimination index for Veh- and CNO-treated CAI mice receiving control AAV (n = 8 mice per group).
In bar graphs, data are shown as the mean ± s.e.m, and each dot represents an individual animal. n indicates individual mice. For **a**, **f**, **g, k,** and **l**, data were collected from 3 independent cohorts and pooled for analysis. For **d**, data were collected from 2 independent cohorts and pooled for analysis. For the others, data were collected from a single cohort. *$p < 0.05$, **$p < 0.01$; n.s., not significant (unpaired two-tailed *t*-test). Further details of the statistical analysis are available in the **Supplementary Data S8**. Source data are provided as a Source Data file.

# Supplementary Fig. 3

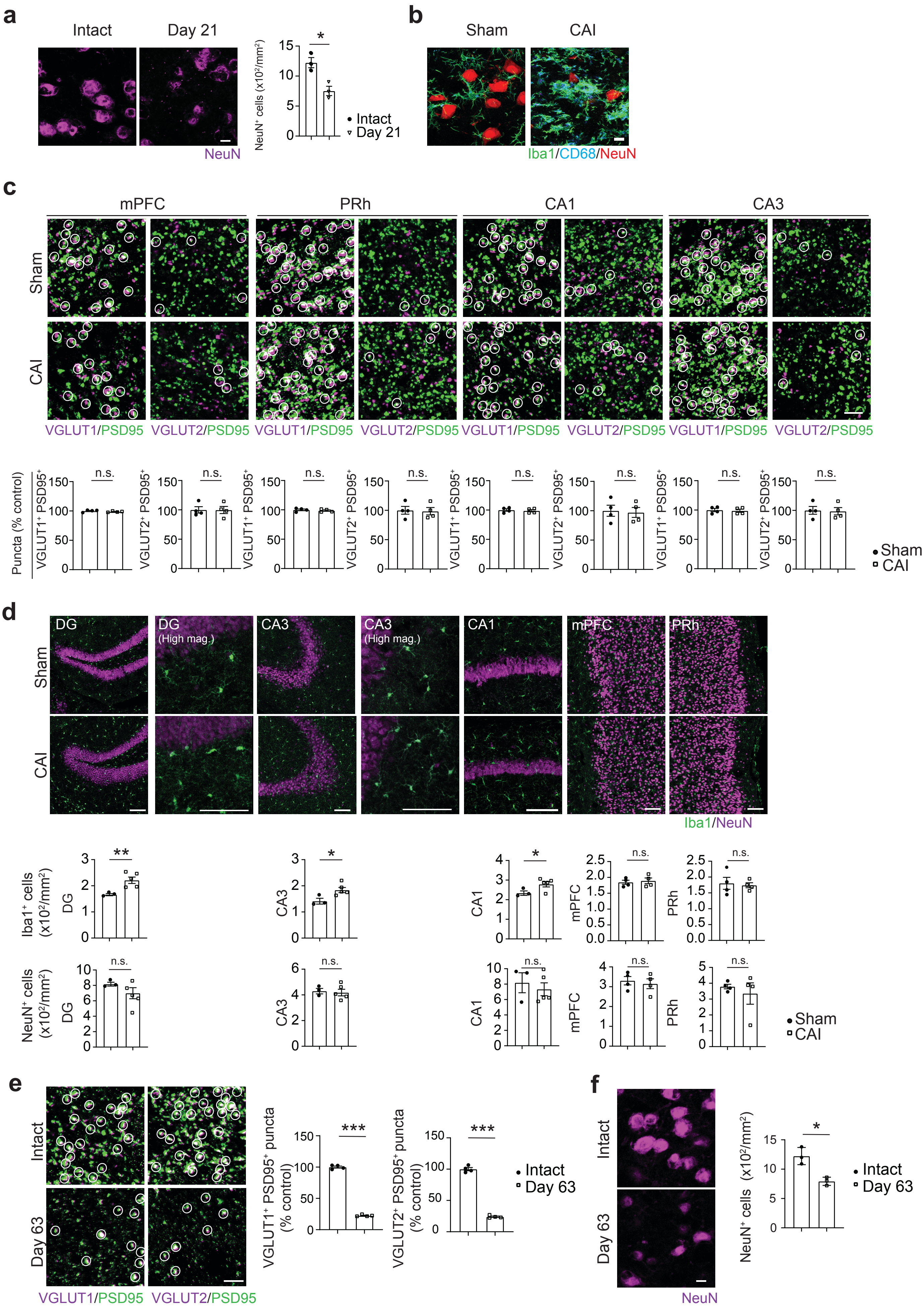

**Supplementary Fig. 3. CAI has little effect on microglial reactive changes, neuronal loss, and synaptic loss in brain regions other than the thalamus.**

**a,** Representative images (left) and quantification (right) of $NeuN^+$ neurons in the thalamus of intact mice and CAI mice on day 21. Scale bar, 10 µm. Quantification data show the number of $NeuN^+$ cells in the thalamus (n = 3 mice per group).

**b,** Representative images of $Iba1^+$ and $NeuN^+$ cells showing no overlap in the thalamus 21 days after CAI and sham surgery. $CD68^+$ area in $Iba1^+$ cells correspond to lysosomes. Scale bar, 10 µm.

**c,** Representative images (left) and quantification (right) of VGLUT1/PSD95 and VGLUT2/PSD95 synapses in the mPFC, PRh, and HPC 21 days after CAI or sham surgery. Scale bar, 2 µm. Quantification data show the synapse density in the mPFC, PRh, and HPC of the CAI group relative to that of the sham group (n = 4 mice per group).

**d,** Representative images (top) and quantification (bottom) of $Iba1^+$ cells and $NeuN^+$ neurons in the HPC (DG, CA3, and CA1), the mPFC, and the PRh 21 days after CAI or sham surgery. Scale bars, 100 µm. Quantification data show their density in each region (n = 3 mice for sham; n = 5 mice for CAI).

**e,** Representative images (top) and quantification (bottom) of VGLUT1/PSD95 and VGLUT2/PSD95 synapses in the thalamus of mice 63 days after CAI and of intact mice. Scale bar, 2 µm. Quantification data show the synapse density in the thalamus of the CAI mice relative to that of intact mice (n = 3 mice per group).

**f,** Representative images (top) and quantification (bottom) of $NeuN^+$ neurons in the thalamus of mice 63 days after CAI and of intact mice. Scale bar, 10 µm. Quantification data show the number of NeuN (n = 3 mice per group).

In bar graphs, data are shown as the mean ± s.e.m, and each dot represents an individual animal. n indicates individual mice. Data were collected from a single cohort with multiple mice. $*p < 0.05$, $**p < 0.01$, $***p < 0.001$; n.s., not significant (unpaired two-tailed *t*-test). Further details of the statistical analysis are available in the **Supplementary Data S8**. Source data are provided as a Source Data file.

# Supplementary Fig. 4

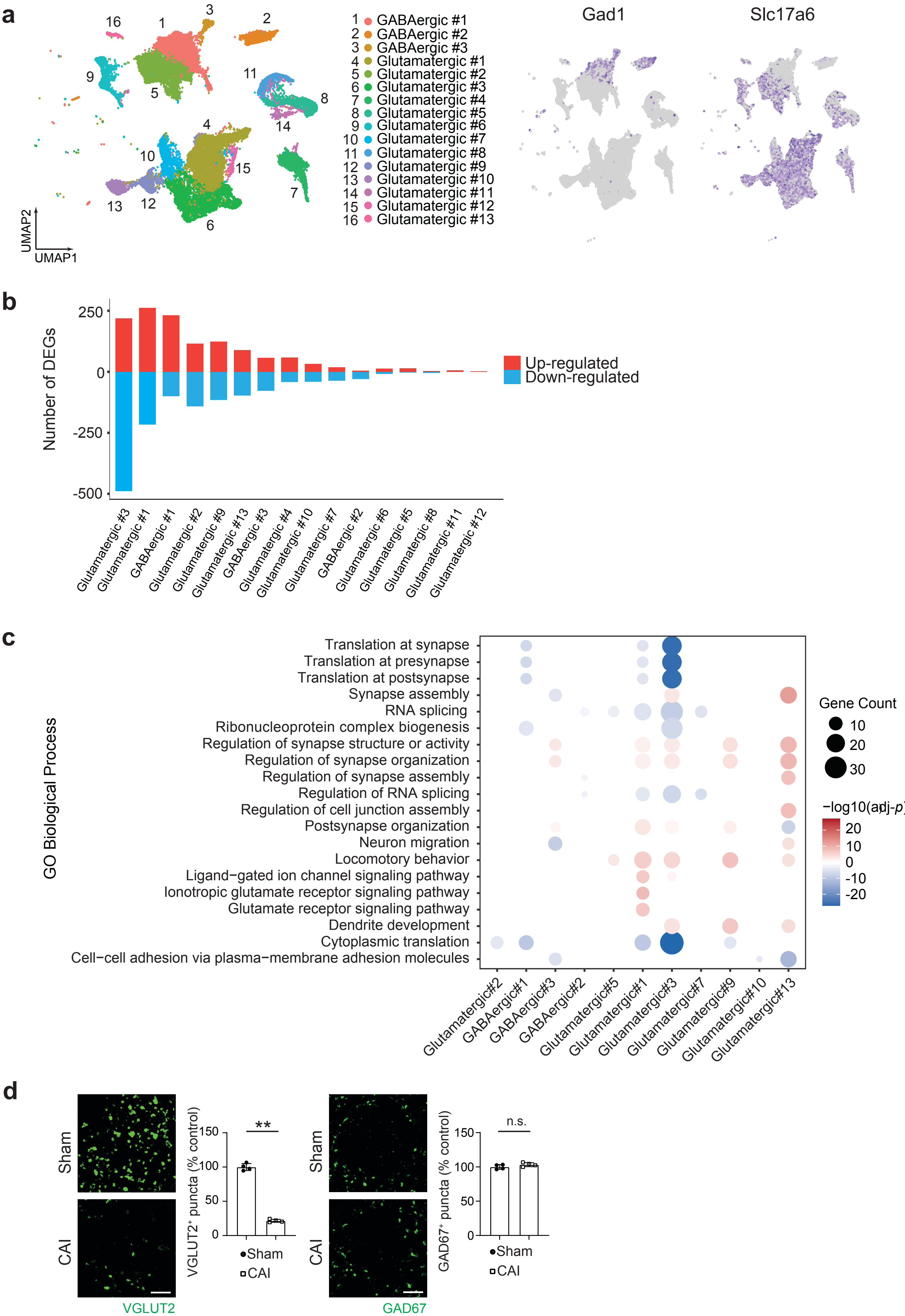

**Supplementary Fig. 4. CAI preferentially affects excitatory neurons in the thalamus.**

**a**, UMAP plots of neuronal clusters identified from snRNA-seq data and feature plots showing the expression of *Gad1* (gene for GAD67) and *Slc17a6* (gene encoding VGLUT2) across clusters.

**b,** Bar plot showing the number of differentially expressed genes (DEGs), both up-regulated and down-regulated, by CAI per cluster.

**c**, Enrichment of gene ontology (GO) biological process categories across the DEGs across neuronal clusters. Dot color represents enrichment score (−log10 adjusted *P* value; directionality indicated by color scale), and dot size represents the number of DEGs contributing to each pathway.

**d,** Representative images (left) and quantification (right) for $VGLUT2^+$ excitatory or $GAD67^+$ inhibitory neuron termini in the thalamus 21 days after CAI or sham surgery. Scale bars, 2 µm. Quantification data show the density of $VGLUT2^+$ and $GAD67^+$ puncta in the CAI group relative to that of the sham group (n = 4 mice per group).

In bar graphs, data are shown as the mean ± s.e.m, and each dot represents an individual animal. n indicates individual mice. Data were collected from a single cohort with multiple mice. $^{**}p < 0.01$; n.s., not significant (unpaired two-tailed *t*-test). Further details of the statistical analysis are available in the **Supplementary Data S8**. Source data are provided as a Source Data file.

# Supplementary Fig. 5

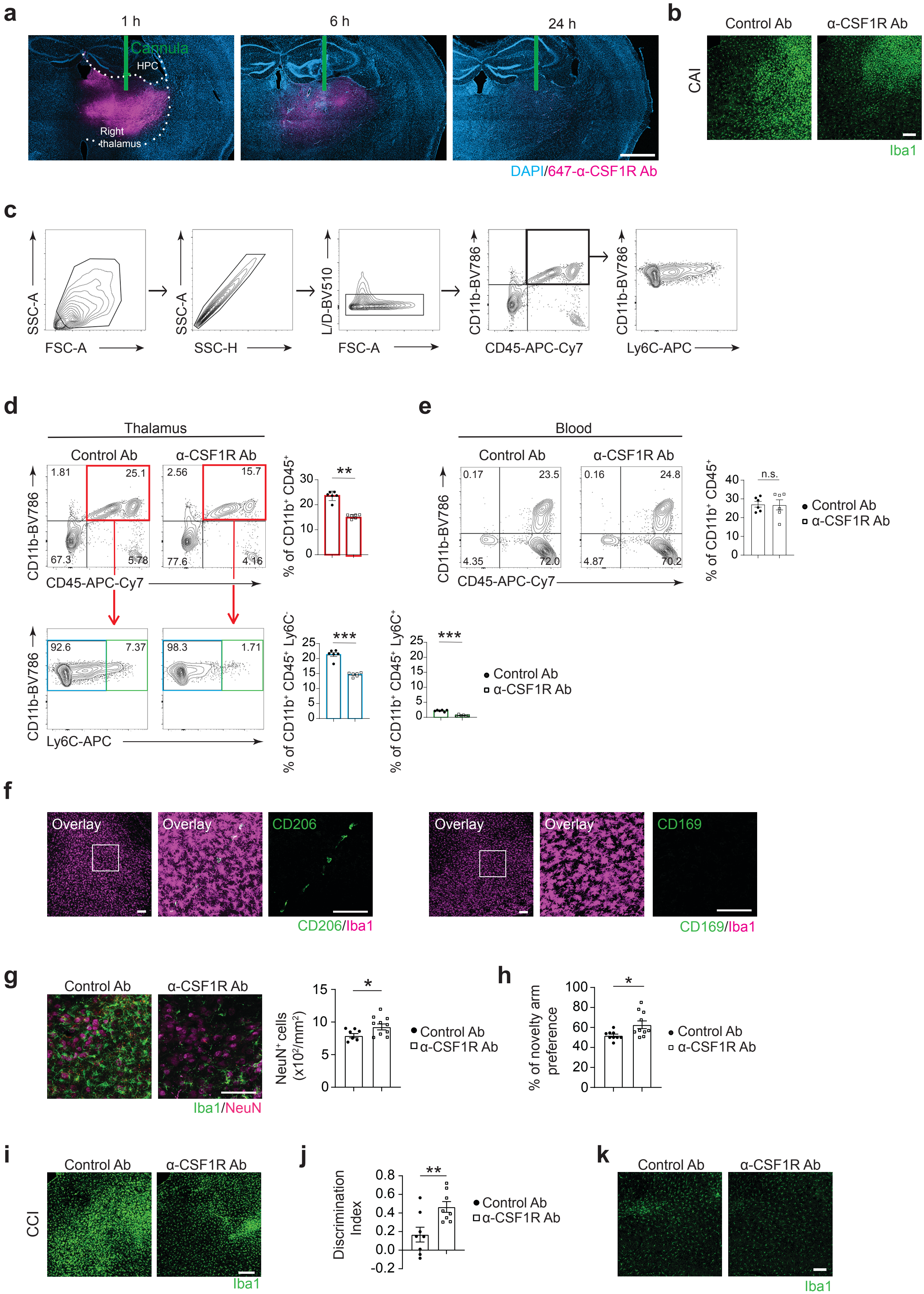

**Supplementary Fig. 5. Local anti-CSF1R Ab injections significantly reduce microglia and improve cognitive impairment in both CAI and CCI mice.**

**a,** Representative images showing the distribution of Alexa Fluor 647-labelled anti-CSF1R Ab 1, 6, and 24 h after intra-thalamic injection via an implanted cannula. Scale bar, 1 mm.

**b**, Representative images of $Iba1^+$ cells in the thalamus of mice receiving intra-thalamic injections of control Ab or anti-CSF1R Ab after CAI. Scale bar, 100 μm.

**c**, Flow cytometry gating strategy used in this study. Microglia and infiltrating macrophages are defined as $CD11b^+CD45^+$ $Ly6C^–$ and $CD11b^+CD45^+$ $Ly6C^+$ cells, respectively.

**d**, Flow cytometry analysis showing the effects of intra-thalamic anti-CSF1R Ab or control Ab injections on total $CD11b^+CD45^+$ cells, $CD11b^+CD45^+Ly6C^+$ cells, and $CD11b^+CD45^+Ly6C^-$ cells in the thalamus of mice after CAI. Left: representative contour and histogram plots. Right: quantification data for mice receiving control Ab and anti-CSF1R Ab (n = 6 per group).

**e**, Flow cytometry analysis showing the effects of intra-thalamic anti-CSF1R Ab or control Ab injections on total $CD11b^+CD45^+$ cells in the blood. Left: representative contour plots. Right: quantification data for mice receiving control Ab and anti-CSF1R Ab (n = 6 per group).

**f**, Representative images of $CD206^+$ $Iba1^+$ border associated macrophages and $CD169^+Iba1^+$ choroid plexus macrophages in the thalamus 21 days after CAI. Note that $CD169^+Iba1^+$ cells are not detected in the thalamic inflammatory area. Scale bars, 100 μm.

**g**, Representative images of $Iba1^+$ cells and $NeuN^+$ neurons in the thalamus of mice receiving intra-thalamic anti-CSF1R Ab or control Ab injections after CAI. Quantification data show the number of $NeuN^+$ neurons (n = 7 mice for control Ab group; n = 10 mice for anti-CSF1R Ab group). Scale bar, 100 μm.

**h**, Y-maze test data. Percentages of time spent in the novel arm are shown for mice receiving control Ab or anti-CSF1R Ab group after CAI (n = 7 mice for control Ab; n = 10 mice for anti-CSF1R Ab).

**i**, Representative images of $Iba1^+$ cells in the thalamus of mice receiving intra-thalamic injections of control Ab or anti-CSF1R Ab after CCI. Scale bar, 100 μm.

**j**, NOR test data. Discrimination index is shown for mice receiving intra-thalamic injections of control Ab and anti-CSF1R Ab (n =8 mice per group) after CCI.

**k**, Representative images of $Iba1^+$ cells in the HPC of mice receiving intra-hippocampal injections of control Ab or anti-CSF1R Ab after CAI. Scale bar, 100 μm.

In bar graphs, data are shown as the mean ± s.e.m, and each dot represents an individual animal. n indicates individual mice. For **g**, **h**, and **i**, data were collected from 3 independent cohorts and pooled for analysis. For **d and e**, data were collected from 2 independent cohorts and pooled for analysis. For the others, data were collected from a single cohort. $^*p < 0.05$, $^{**}p < 0.01$, $^{***}p < 0.001$; n.s., not significant (unpaired two-tailed *t*-test). Further details of the statistical analysis are available in the **Supplementary Data S8**. Source data are provided as a Source Data file.

# Supplementary Fig. 6

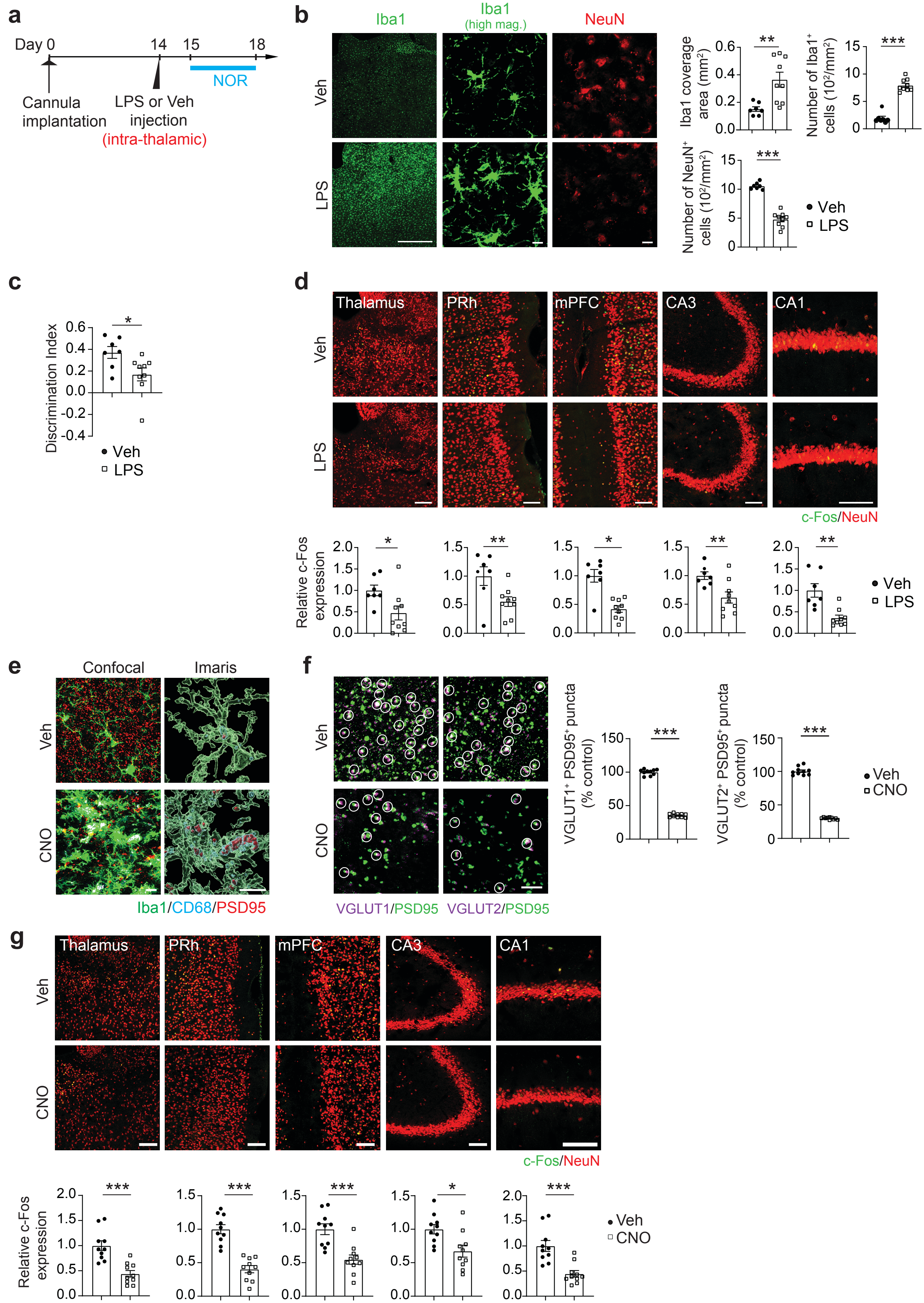

**Supplementary Fig. 6. Reactive microglial changes induced by intra-thalamic LPS injection and chemogenetics cause cognitive impairment in uninjured mice.**

**a**, Experimental timeline for cannula implantation, intra-thalamic LPS or vehicle (Veh) injection, and the NOR test.

**b**, Representative images (left) and quantification (right) of Iba1$^{+}$ cells and NeuN$^{+}$ neurons in the thalamus on day 18 (4 days after vehicle or LPS injection). Scale bars: 500 µm (left low magnification image) and 10 µm (middle and right high magnification images). Quantification data show the Iba1 coverage area, the number of Iba1$^{+}$ cells, and the number of NeuN$^{+}$ neurons in the thalamus (n = 7 mice for the vehicle-injected group; n = 9 mice for the LPS-injected group).

**c**, Discrimination index in the NOR test (n = 7 mice for the vehicle-injected group; n = 9 mice for the LPS-injected group).

**d**, Representative images (top) and quantification (bottom) of neuronal c-Fos expression in the thalamus, PRh, mPFC, and HPC (CA3 and CA1) 4 days after vehicle or LPS injection. Scale bars: 100 µm. Quantification data show neuronal c-Fos expression in the LPS-injected group (n = 9 mice) relative to the vehicle-injected mice (n = 7 mice).

**e**, Representative confocal and Imaris-reconstructed 3D images of PSD95 puncta inside thalamic Iba1$^{+}$ cells after vehicle or CNO injection. Scale bars, 10 µm.

**f**, Representative images (left) and quantification (right) of VGLUT1/PSD95 and VGLUT2/PSD95 synapses in the thalamus of the vehicle-injected or CNO-injected mice. Scale bar, 2 µm. Quantification data show the synapse density in the thalamus of the CNO-injected group relative to that of the vehicle-injected group (n = 10 mice per group).

**g**, Representative images (top) and quantification (bottom) of neuronal c-Fos expression in the thalamus, PRh, mPFC, and HPC (CA3, CA1) of the vehicle-injected or CNO-injected mice. Scale bars, 100 µm. Quantification data show neuronal c-Fos expression in the CNO-injected group relative to the vehicle-injected mice (n = 10 mice per group).

In bar graphs, data are shown as the mean ± s.e.m, and each dot represents an individual animal. n indicates individual mice. Data were collected from 3 independent cohorts and pooled for analysis. *$p < 0.05$, **$p < 0.01$, ***$p < 0.001$ (unpaired two-tailed *t*-test). Further details of the statistical analysis are available in the **Supplementary Data S8**. Source data are provided as a Source Data file.

# Supplementary Fig. 7

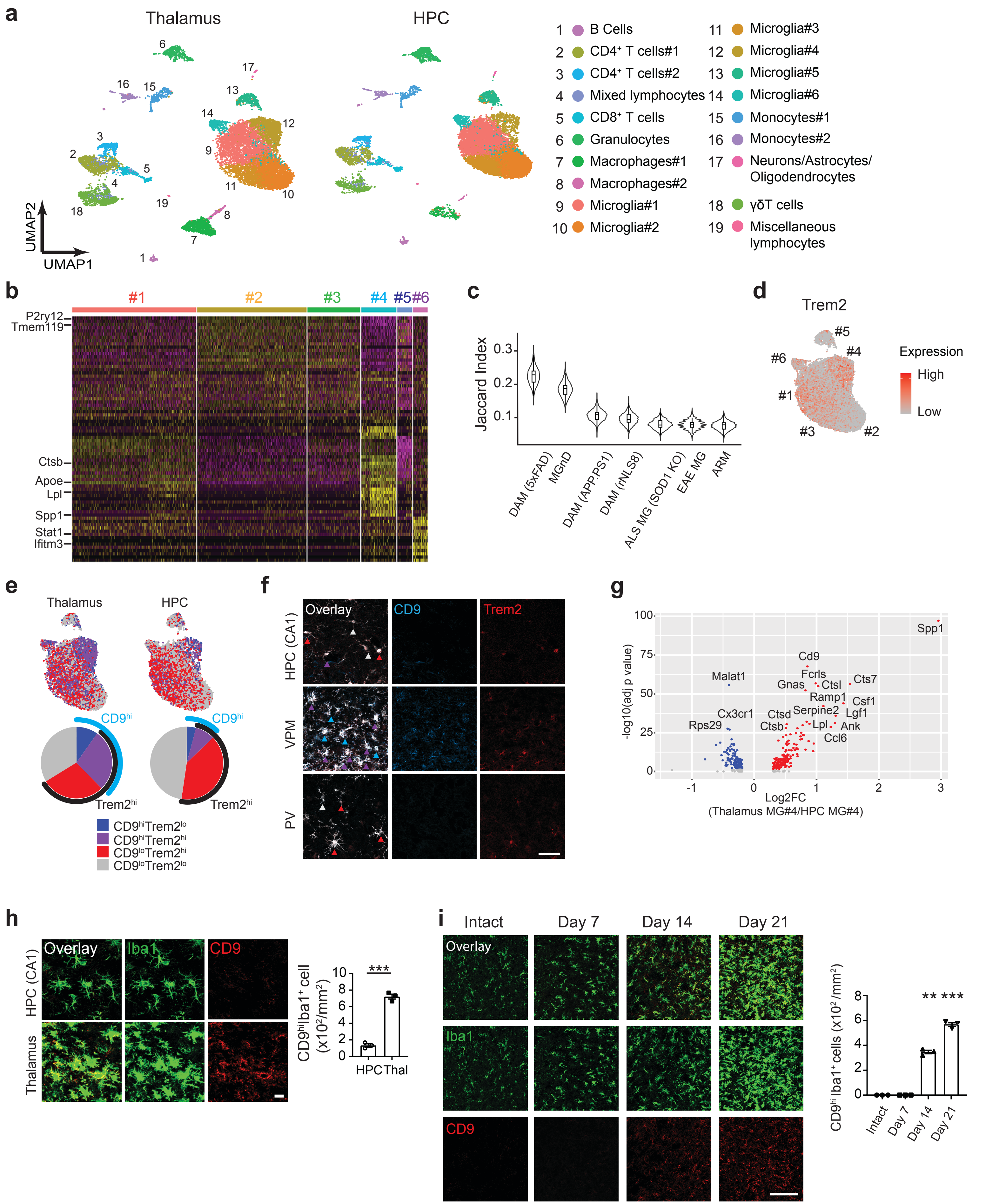

**Supplementary Fig. 7. Microglia with distinct transcriptional states appear in the thalamus and HPC of mice after cortical damage.**

**a**, UMAP plots showing 19 clusters with 6 microglia subclusters in the hippocampus and the thalamus after CAI.

**b**, Heatmap showing unbiased top markers defining each microglia state after CAI.

**c**, Similarity of MG#4 to previously reported DAM and DAM-related gene signatures. Higher Jaccard indices suggest more similarity.

**d**, Feature plot showing Trem2 expression in all microglia clusters of the thalamus and HPC after CAI.

**e**, Feature plots showing CD9 (blue) expression and Trem2 (red) expression in all microglia clusters of the thalamus and HPC after CAI. Pie charts showing the proportion of $CD9^{hi}Trem2^{lo}$, $CD9^{hi}Trem2^{hi}$, $CD9^{lo}Trem2^{hi}$, and $CD9^{lo}Trem2^{lo}$ microglia clusters of the thalamus and HPC after CAI.

**f**, Spatial distribution of $CD9^{hi}$ microglia and $Trem2^{hi}$ microglia in the thalamus after CAI. Shown are representative images of $CD9^{hi}$ $Trem2^{hi}$ microglia (purple arrowheads), $CD9^{hi}$ $Trem2^{lo}$ microglia (blue arrowheads), $CD9^{lo}$ $Trem2^{hi}$ microglia (red arrow heads), and $CD9^{lo}$ $Trem2^{lo}$ microglia (gray arrowheads) in the HPC, ventral posteromedial nucleus (VPM), and paraventricular nucleus (PV). Scale bar, 50 μm.

**g**, Volcano plot of differentially expressed genes in the thalamic MG#4 compared to the HPC MG#4. Red: genes upregulated in the thalamic MG#4. Blue: genes downregulated in the thalamic MG#4.

**h**, Representative images of $CD9^{hi}Iba1^{+}$ cells in the HPC and thalamus after CAI. Scale bar, 10 μm. Quantification data show the number of $CD9^{hi}$ $Iba1^{+}$ cells in the HPC (n = 3) and the thalamus (n = 3).

**i**, Time-course analysis of $CD9^{hi}Iba1^{+}$ cell dynamics in the thalamus. Representative images (left) and quantification (right) of $CD9^{hi}Iba1^{+}$ cells in the thalamus of mice 7, 14, and 21 days after CAI and of intact mice. Scale bar, 100 μm. Quantification data show the number of $CD9^{hi}$ $Iba1^{+}$ cells (n = 3 mice).

In bar graphs, data are shown as the mean ± s.e.m, and each dot represents an individual animal. Data were collected from a single cohort with multiple mice. $**p < 0.01$, $***p < 0.001$ [unpaired two-tailed *t*-test (h) and one-way ANOVA with post-hoc Dunnett's test (i)]. Further details of the

statistical analysis are available in the **Supplementary Data S8**. Source data are provided as a Source Data file.

# Supplementary Fig. 8

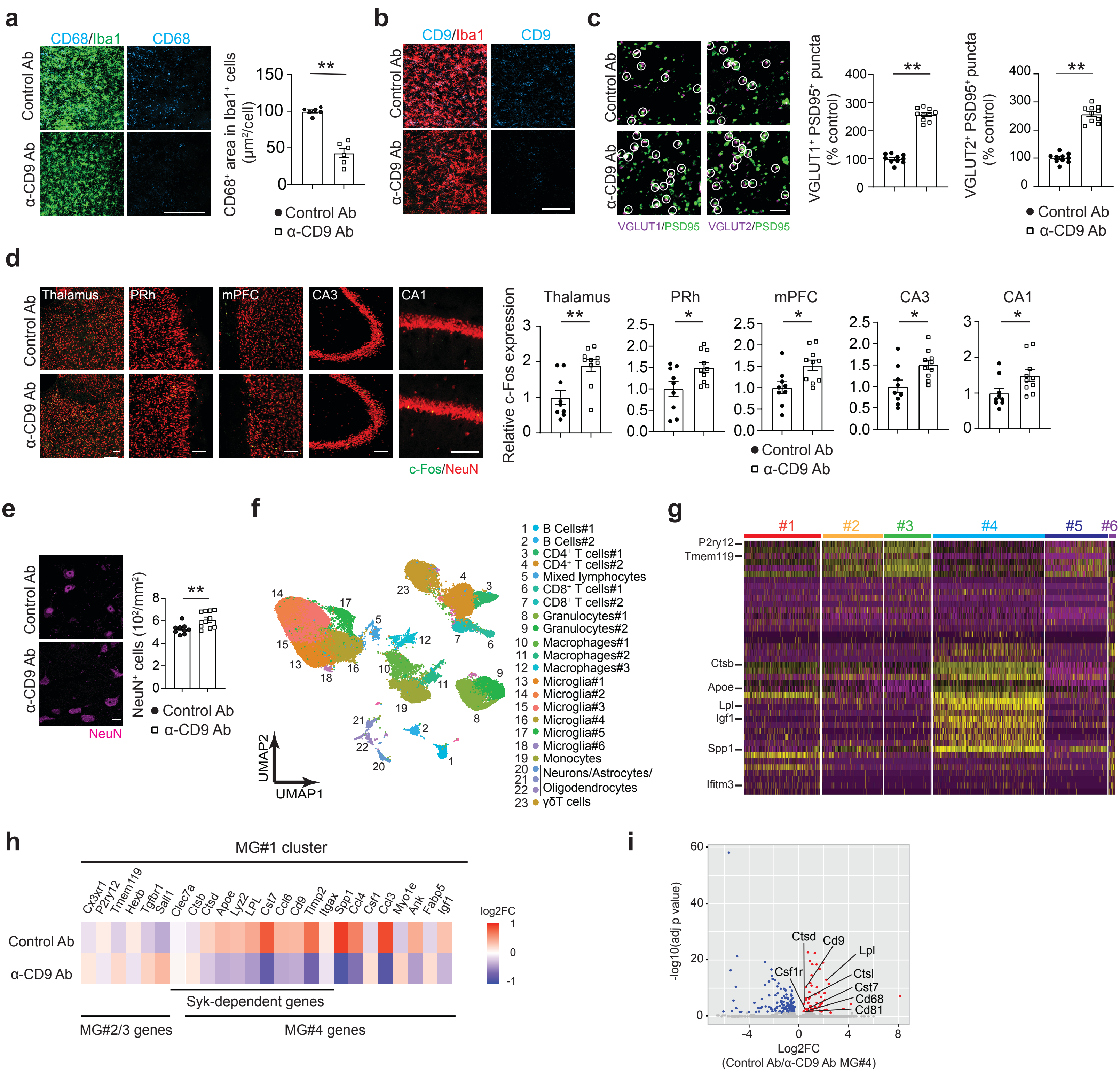

**Supplementary Fig. 8. CD9 blockade inhibits MG#4 induction in the thalamus of mice after cortical damage.**

**a**, Representative images (left) and quantification (right) of $CD68^+$ lysosomes in $Iba1^+$ cells in the thalamus from the control Ab- or anti-CD9 Ab-injected mice 21 days after CAI. Scale bar, 100 µm. Quantification data show average $CD68^+$ area per cell for control Ab-injected (n= 9 mice) and anti-CD9 Ab-injected group (n = 10 mice).

**b**, Representative images of $CD9^{hi}Iba1^+$ cells in the thalamus from the control Ab-injected or anti-CD9 Ab-injected mice 21 days after CAI. Scale bar, 100 µm. Antibodies used to detect CD9 are from different clones than those used for injections.

**c**, Representative images (left) and quantification (right) of VGLUT1/PSD95 and VGLUT2/PSD95 synapses in the thalamus from the control Ab-injected or anti-CD9 Ab-injected mice 21 days after CAI. Scale bar, 2 µm. Quantification data show the synapse density in the thalamus of the anti-CD9 Ab-injected group (n = 10 mice) relative to that of the control Ab-injected group (n = 9 mice).

**d**, Representative images (left) and quantification (right) of neuronal c-Fos expression in the thalamus, PRh, mPFC, and HPC (CA3 and CA1) from the control Ab-injected or anti-CD9 Ab-injected mice 21 days after CAI. Scale bars, 100 µm. Quantification data show neuronal c-Fos expression of the anti-CD9 Ab-injected group (n = 10 mice) relative to the control Ab-injected group (n = 9 mice).

**e,** Representative images (left) and quantification (left) of $NeuN^+$ neurons in the thalamus from the control Ab-injected and anti-CD9 Ab-injected mice 21 days after CAI. Scale bar, 10 µm. Quantification data show the number of $NeuN^+$ cells in the thalamus (n = 9 mice for the control Ab-injected group; n = 10 mice for the anti-CD9 Ab-injected group).

**f,** UMAP plot showing 23 cell clusters, including 6 microglia subclusters in the thalamus of the control Ab- and anti-CD9 Ab-injected mice 21 days after CAI.

**g**, Heatmap showing unbiased top markers defining each microglia subtype in the thalamus of the control Ab- and anti-CD9 Ab-injected mice 21 days after CAI.

**h**, Pseudo-bulk heat map showing log2FC of mean gene expression in thalamic MG#1 between the anti-CD9 Ab group and the control Ab group, highlighting up- or down-regulated genes that are Syk-dependent or enriched in MG#2/3, MG#4, and MG#6. Positive values indicate higher expression in red and negative values indicate lower expression in blue.

**i**, Volcano plot of differentially expressed genes in the thalamic MG#4 microglia in the anti-CD9 Ab group compared to the thalamic MG#4 microglia in the control Ab group. Red: genes downregulated in the anti-CD9 Ab group. Blue: genes upregulated in anti-CD9 Ab group.
In bar graphs, data are shown as the mean ± s.e.m, and each dot represents an individual animal. n indicates individual mice. For **c**, **d**, and **e**, data were collected from 3 independent cohorts and pooled for analysis. For **a**, data were collected from 2 independent cohorts and pooled for analysis. For the others, data were collected from a single cohort. n.s., not significant; $*p < 0.05$, $**p < 0.01$ (unpaired two-tailed *t*-test). Further details of the statistical analysis are available in the **Supplementary Data S8**. Source data are provided as a Source Data file.

# Supplementary Fig. 9

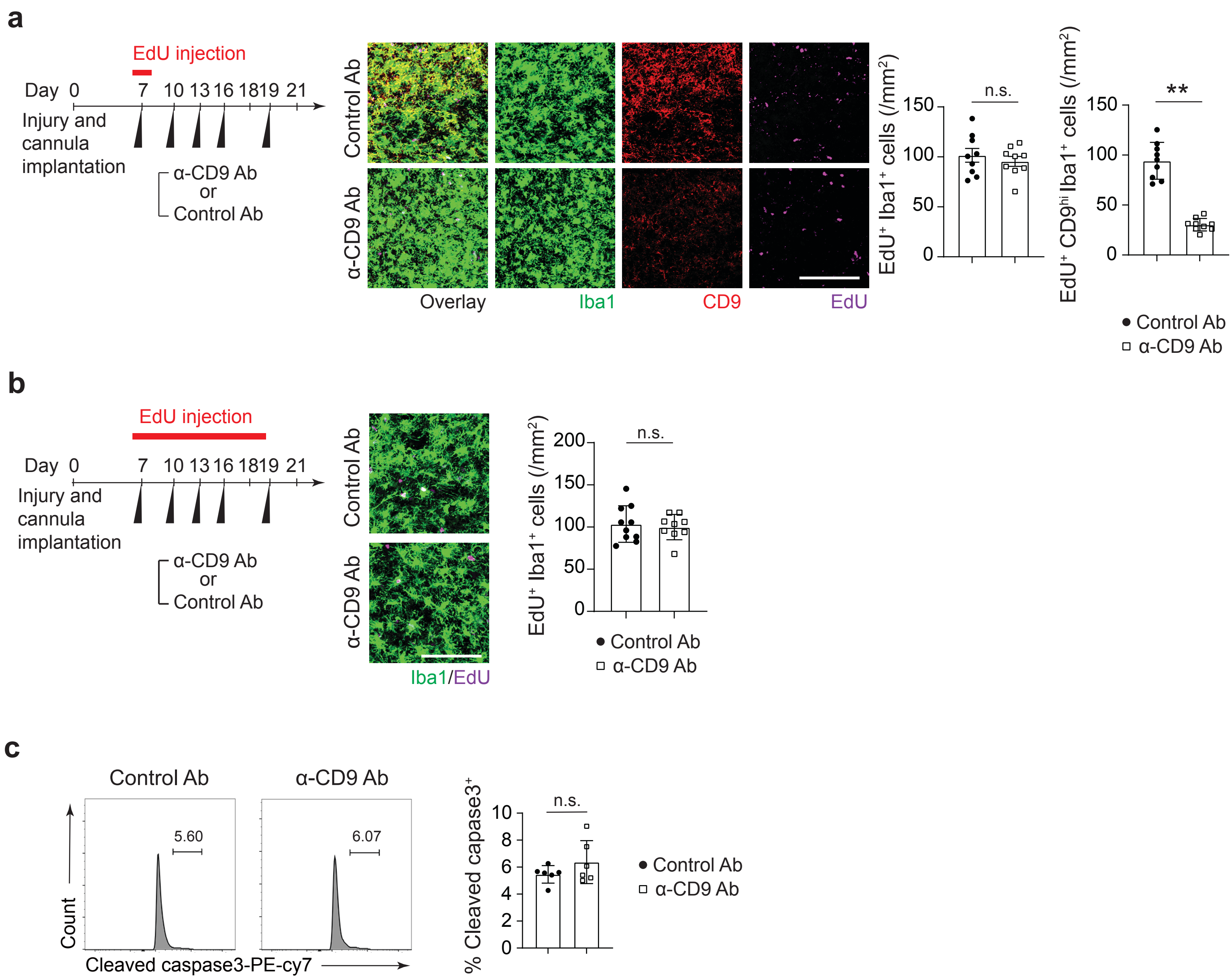

**Supplementary Fig. 9. CD9 plays a critical role in the generation of phagocytic microglia in the thalamus of mice with cortical damage.**

**a,** Experimental timeline for single EdU injection and anti-CD9 Ab or control Ab injections. Representative images show $EdU^+CD9^{hi}Iba1^+$ cells in the thalamus of mice receiving control Ab or anti-CD9 Ab 21 days after CAI. Scale bar, 100 µm. Quantification data show the numbers of $EdU^+Iba1^+$ cells and $EdU^+CD9^+Iba1^+$ cells in the thalamus for control Ab- injected and anti-CD9 Ab-injected group (n = 9 mice per group).

**b,** Experimental timeline for repeated EdU injections and anti-CD9 Ab or control Ab injections. Representative images show $EdU^+Iba1^+$ cells in the thalamus of mice receiving control Ab and anti-CD9 Ab 21 days after CAI. Scale bar, 100 µm. Quantification data show the number of $EdU^+Iba1^+$ cells in the thalamus for control Ab-injected (n = 9 mice) and anti-CD9 Ab-injected (n = 10 mice) group.

**c**, Representative histogram plots (left) and quantification (right) of cleaved Caspase-$3^+$ MG#4 ($CD9^{hi}Tmem119^{lo}CD11b^+CD45^+Ly6C^-$ cells) in the thalamus of control Ab- and anti-CD9 Ab-injected mice 21 days after CAI. Quantification data show % of cleaved Caspase-$3^+$ cells within MG#4 population for the control Ab-injected and anti-CD9 Ab-injected group (n = 9 mice per group).

In bar graphs, data are shown as the mean ± s.e.m, and each dot represents an individual animal. For **a**, and **b**, data were collected from 3 independent cohorts and pooled for analysis. For **c**, data were collected from 2 independent cohorts and pooled for analysis. $**p < 0.01$, $***p < 0.001$; n.s., not significant (unpaired two-tailed *t*-test). Further details of the statistical analysis are available in the **Supplementary Data S8**. Source data are provided as a Source Data file.

# Supplementary Fig. 10

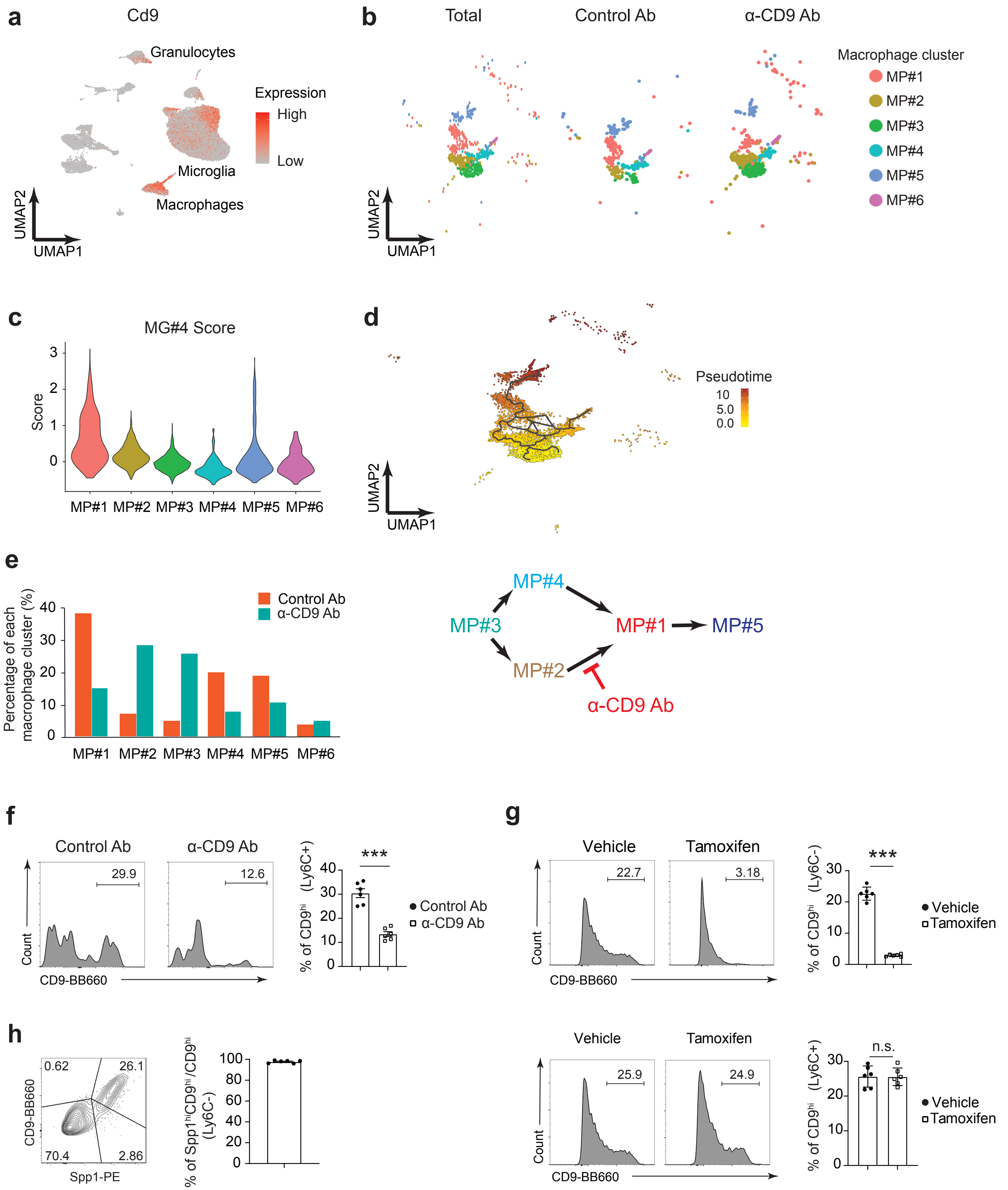

**Supplementary Fig. 10. CD9 also plays a critical role in the generation of phagocytic macrophages, in addition to microglia, in the thalamus of mice with cortical damage.**

**a,** Feature plots showing Cd9 expression across all clusters in the thalamus.

**b,** UMAP plots highlighting 6 macrophage clusters in the thalamus of the control Ab-injected group and the anti-CD9 Ab-injected mice 21 days after CAI.

**c,** The MG#4 scores showing the similarities of gene expression patterns in macrophage clusters (MP#1-6) to MG#4.

**d,** Pseudo-time analysis showing a cell transition trajectory from MP#2 and MP#3 to MP#1 and MP#5. A schematic diagram highlights the potential transition step that anti-CD9 Ab blocks.

**e,** Bar graphs showing the percentages of each macrophage cluster in the control Ab group and in the anti-CD9 Ab group, with the percentages reaching 100% for each group.

**f,** Representative flow cytometry plots (left) and quantification (right) of $CD9^{hi}$ cells in $Ly6C^+$ cells in the thalamus (gated from $CD11b^+CD45^+$ cells) of control Ab- and anti-CD9 Ab-injected mice 21 days after CAI. Quantification data show % of $CD9^{hi}$ cells within $Ly6C^+$ population (n = 6 mice per group).

**g**, Representative flow cytometry plots (left) and quantification (right) of $CD9^{hi}$ cells in $Ly6C^-$ and $Ly6C^+$ cells in the thalamus (gated from $CD11b^+CD45^+$ cells) of vehicle- and tamoxifen-injected *Tmem119*$^{CreERT2}$*Cd9*$^{flox/flox}$ mice 21 days after CAI. Quantification data show % of $CD9^{hi}$ cells within $Ly6C^-$ and $Ly6C^+$ population (n = 6 mice per group).

**h**, Representative flow cytometry plots (left) and quantification (right) of $CD9^{hi}$ and $Spp1^{hi}$ cells within $CD11b^+CD45^+$ $Ly6C^–$ cells in the thalamus 21 days after CAI. Quantification data show % of $Spp1^{hi}$ cells within $CD9^{hi}$ cells (n = 4 mice).

In bar graphs, data are shown as the mean ± s.e.m, and each dot represents an individual animal. n indicates individual mice. For **f,** and **g**, data were collected from 2 independent cohorts and pooled for analysis. For the others, data were collected from a single cohort. ***$p < 0.001$; n.s., not significant (unpaired two-tailed *t*-test). Further details of the statistical analysis are available in the **Supplementary Data S8**. Source data are provided as a Source Data file.

# Supplementary Fig. 11

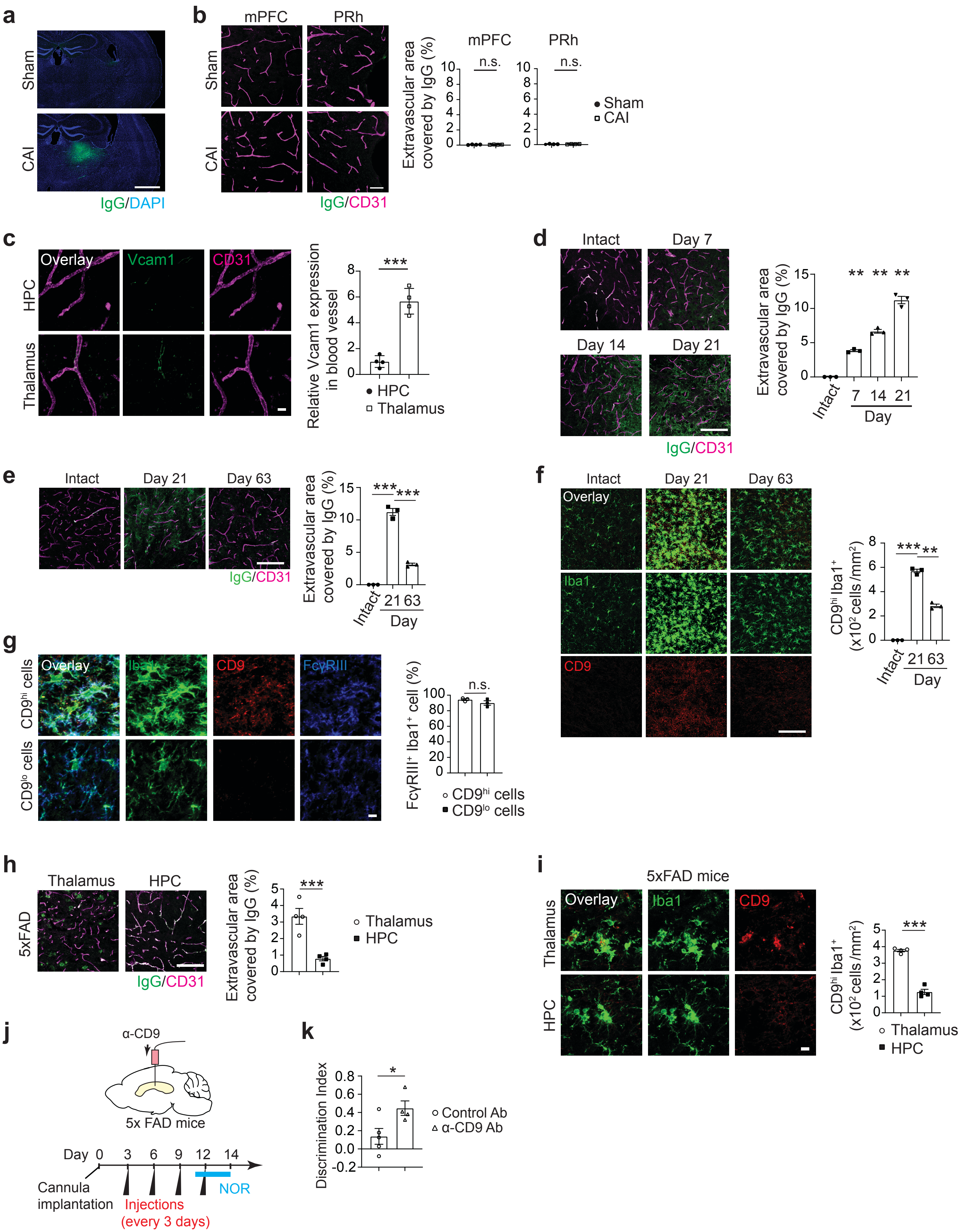

**Supplementary Fig. 11. IgG extravasation and endothelial changes are associated with the emergence of $CD9^{hi}Iba1^{+}$ cells in the thalamus.**

**a**, Representative low-magnification images of IgG and DAPI in the ipsilateral hemisphere 21 days after CAI or sham surgery. Scale bar, 1 mm.

**b**, Representative images (left) and quantification (right) of IgG and $CD31^{+}$ blood vessels in the mPFC and PRh 21 days after CAI or sham surgery. Scale bar, 50 µm. Quantification data show the percentages of area covered by extravascular IgG relative to total IgG for the CAI and the sham group (n = 4 mice per group).

c, Representative images (left) and quantification (right) of Vcam1 expression in $CD31^{+}$ blood vessels in the HPC and the thalamus 21 days after CAI. Scale bar, 10 µm. Quantification data show the Vcam1 expression in $CD31^{+}$ blood vessels in the thalamus relative to that in the HPC (n = 4 mice per region).

**d**, Representative images (left) and quantification (right) of IgG and $CD31^{+}$ blood vessels in the thalamus of mice 7, 14, and 21 days after CAI and of intact mice. Scale bar, 100 µm. Quantification data show the percentages of area covered by extravascular IgG relative to total IgG for each time point (n = 3 mice).

**e**, Representative images (left) and quantification (right) of IgG and $CD31^{+}$ blood vessels in the thalamus of mice 21 and 63 days after CAI and of intact mice. Scale bar, 100 µm. Quantification data show the percentages of area covered by extravascular IgG relative to total IgG for each time point (n = 3 mice).

**f**, Representative images (left) and quantification (right) of $CD9^{hi}$ $Iba1^{+}$ cells in the thalamus of mice 21 and 63 days after CAI and of intact mice. Scale bar, 100 µm. Quantification data show the density of $CD9^{hi}$ $Iba1^{+}$ cells in the thalamus for each time point (n = 3 mice).

**g,** Representative images (left) and quantification (right) of $Fc\gamma RIII^{+}$ cells within the $Iba1^{+}CD9^{hi}$ and $Iba1^{+}CD9^{lo}$ cells in the thalamus 21 days after CAI. Scale bar, 10 µm. Quantification data show the percentages of $Fc\gamma RIII^{+}$ cells within $Iba1^{+}$ $CD9^{+}$ and $Iba1^{+}$ $CD9^{-}$ cells (n = 3 mice per cell type).

**h**, Representative images (left) and quantification (right) of IgG and $CD31^{+}$ blood vessels in the thalamus and the HPC in 6-month-old female 5xFAD mice. Scale bar, 100 µm. Quantification data show the percentages of area covered by extravascular IgG relative to total IgG for the thalamus and the HPC (n = 4 mice per region).

**i**, Representative images (left) and quantification (right) of $CD9^{hi}$ $Iba1^{+}$ cells in the thalamus and the HPC of 5xFAD mice. Scale bar, 10 µm. Quantification data show the density of $CD9^{hi}$ $Iba1^{+}$ cells for the thalamus and the HPC (n = 4 mice per region).
**j**, Experimental timeline for anti-CD9 Ab or control Ab injection for 5xFAD mice.
**k,** Discrimination index in the NOR test (n = 4 mice for the control Ab-injected group; n = 5 mice for the anti-CD9 Ab-injected group).
In bar graphs, data are shown as the mean ± s.e.m, and each dot represents an individual animal. n indicates individual mice. For **k**, data were collected from 2 independent cohorts and pooled for analysis. For the others, data were collected from a single cohort. *$p < 0.05$, **$p < 0.01$, ***$p < 0.001$ [unpaired two-tailed *t*-test (b, c, g, h, i, k), one-way ANOVA with post-hoc Dunnett's test (d), and one-way ANOVA with post-hoc Tukey-Kramer's test (e, f)]. Further details of the statistical analysis are available in the **Supplementary Data S8**. Source data are provided as a Source Data file.

**Supplementary Data**

Data S1. List of genes whose expression was significantly upregulated in MG#1-6.

Data S2. Genes used for Cluster Profiler analysis.

Data S3. Outputs of pseudotime analysis with Monocle 3.

Data S4. List of differentially expressed genes between the thalamus and HPC MG#4.

Data S5. List of differentially expressed genes in MG#1 between control Ab- and anti-CD9 Ab-treated mice.

Data S6. List of differentially expressed genes in MG#4 between control Ab- and anti-CD9 Ab-treated mice.

Data S7. Raw data of NOR test.

Data S8. Details of the statistical analyses.
