# Supplementary material for: FcγR- and CD9-dependent synapse-engulfing microglia in the thalamus drive cognitive impairment following cortical brain damage in mice
Source: Nat Commun. 2026 Jul 9;17:8514. doi: 10.1038/s41467-026-74904-1 (PMC13484472; doi:10.1038/s41467-026-74904-1)
Supplement: Supplementary file 2 [file 41467_2026_74904_MOESM3_ESM.pdf]

nature portfolio

Corresponding author(s): Shin-ichi Kano

Last updated by author(s): May 7, 2026

Reporting Summary

Nature Portfolio wishes to improve the reproducibility of the work that we publish. This form provides structure for consistency and transparency in reporting. For further information on Nature Portfolio policies, see our Editorial Policies and the Editorial Policy Checklist.

Statistics

For all statistical analyses, confirm that the following items are present in the figure legend, table legend, main text, or Methods section.

n/a	Confirmed	
☐	☒	The exact sample size (n) for each experimental group/condition, given as a discrete number and unit of measurement
☐	☒	A statement on whether measurements were taken from distinct samples or whether the same sample was measured repeatedly
☐	☒	The statistical test(s) used AND whether they are one- or two-sided<br>*Only common tests should be described solely by name; describe more complex techniques in the Methods section.*
☒	☐	A description of all covariates tested
☐	☒	A description of any assumptions or corrections, such as tests of normality and adjustment for multiple comparisons
☐	☒	A full description of the statistical parameters including central tendency (e.g. means) or other basic estimates (e.g. regression coefficient) AND variation (e.g. standard deviation) or associated estimates of uncertainty (e.g. confidence intervals)
☐	☒	For null hypothesis testing, the test statistic (e.g. F, t, r) with confidence intervals, effect sizes, degrees of freedom and P value noted<br>*Give P values as exact values whenever suitable.*
☒	☐	For Bayesian analysis, information on the choice of priors and Markov chain Monte Carlo settings
☒	☐	For hierarchical and complex designs, identification of the appropriate level for tests and full reporting of outcomes
☐	☒	Estimates of effect sizes (e.g. Cohen's d, Pearson's r), indicating how they were calculated

Our web collection on statistics for biologists contains articles on many of the points above.

Software and code

Policy information about availability of computer code

Data collection	Zen software (Zeiss) -- Confocal microscopy image data<br>Accuri C6 software, CellQuest, FACSDiva (BD Biosciences) -- Flow cytometry data<br>PAS software -- Behavioral video and quantification data (Novel Object Recognition test and Y-maze test)<br>EthoVision XT17 (Noldus) -- Behavioral video and quantification data (Novel Object Recognition test and Y-maze test)<br>NovaSeq6000-- single-cell and nucleus RNA-seq data
Data analysis	ImageJ/Fiji (ver. 2.3.0/1.53f51)-- immunohistochemical analyses and quantification<br>FlowJo -- flow cytometry data analysis<br>Seurat package (v.4.0) in R (v.4.1.1)-- single-cell RNA-seq and single-nucleus RNA-seq data analysis<br>GraphPad Prism (ver. 7, 8, 9; GraphPad Software, Inc) and R (ver. 4.1.1)-- statistical analysis

For manuscripts utilizing custom algorithms or software that are central to the research but not yet described in published literature, software must be made available to editors and reviewers. We strongly encourage code deposition in a community repository (e.g. GitHub). See the Nature Portfolio guidelines for submitting code & software for further information.

Data

Policy information about availability of data

All manuscripts must include a data availability statement. This statement should provide the following information, where applicable:

- Accession codes, unique identifiers, or web links for publicly available datasets
- A description of any restrictions on data availability
- For clinical datasets or third party data, please ensure that the statement adheres to our policy

The raw scRNA-sequencing and snRNA-sequencing data will be available at the Gene Expression Omnibus (GEO) before publication.

Research involving human participants, their data, or biological material

Policy information about studies with human participants or human data. See also policy information about sex, gender (identity/presentation), and sexual orientation and race, ethnicity and racism.

Reporting on sex and gender	N/A
Reporting on race, ethnicity, or other socially relevant groupings	N/A
Population characteristics	N/A
Recruitment	N/A
Ethics oversight	N/A

Note that full information on the approval of the study protocol must also be provided in the manuscript.

Field-specific reporting

Please select the one below that is the best fit for your research. If you are not sure, read the appropriate sections before making your selection.

☒ Life sciences ☐ Behavioural & social sciences ☐ Ecological, evolutionary & environmental sciences

For a reference copy of the document with all sections, see nature.com/documents/nr-reporting-summary-flat.pdf

Life sciences study design

All studies must disclose on these points even when the disclosure is negative.

Sample size	Sample sizes are determined based on previously published experiments or effect size calculation of pilot data.
Data exclusions	Pre-established exclusion criteria were applied to behavioral assay in order not to analyze the data from the mice who could not complete tasks (e.g., immobile throughout the session, aggressive interactions between the test and stimulus mice).
Replication	All the experiments were conducted with at least 3 biological replicates as shown and described in each Figure and its legend.
Randomization	Mice were randomly assigned into experimental and control groups where applicable.
Blinding	Quantification of immunohistochemistry and mouse behavior data were conducted in a blind manner.

Reporting for specific materials, systems and methods

We require information from authors about some types of materials, experimental systems and methods used in many studies. Here, indicate whether each material, system or method listed is relevant to your study. If you are not sure if a list item applies to your research, read the appropriate section before selecting a response.

Materials & experimental systems

n/a	Involved in the study
[ ]	[x] Antibodies
[x]	[ ] Eukaryotic cell lines
[x]	[ ] Palaeontology and archaeology
[ ]	[x] Animals and other organisms
[x]	[ ] Clinical data
[x]	[ ] Dual use research of concern
[x]	[ ] Plants

Methods

n/a	Involved in the study
[x]	[ ] ChIP-seq
[ ]	[x] Flow cytometry
[x]	[ ] MRI-based neuroimaging

Antibodies

Antibodies used

Immunohistochemistry
rabbit polyclonal anti-Iba1 (1:500; Fuji Film, #019–19741)
Alexa Fluor 488-conjugated rabbit monoclonal anti-Iba1 (1:500; abcam, #ab225260)
mouse monoclonal anti-Iba1 (1:500; Sigma-Aldrich, #MABN92)
Alexa Fluor 647-conjugated mouse monoclonal anti-NeuN (1:500; abcam, #ab190565)
rabbit monoclonal anti-NeuN (1:500; abcam, #ab177487)
rabbit monoclonal anti-c-Fos (1:500; Cell Signaling Technology, #2250)
rabbit monoclonal anti-TSPO (1:100; abcam, #ab109497)
rat monoclonal anti-CD68 (1:2,000; Novus Biologicals, #NBP2-33337)
mouse monoclonal anti-TNF-α (1:100; abcam, #ab1793)
rat monoclonal anti-RFP (1:500; Chromotek, #5F8)
rabbit monoclonal anti-HA-tag (1:500; Cell Signaling Technology, #3724)
armenian hamster monoclonal anti-CD31/PECAM1 (1:100; DSHB, #2H8-s)
rabbit polyclonal CD9 (1:100; Proteintech, #20597-1-AP)
rat monoclonal anti-CD9 (1:100; BD Biosciences, #553758)
rabbit polyclonal anti-FcγRIII (1:100; abcam, #ab203883)
rabbit polyclonal anti-p-Syk (1:200; Cell Signaling, #2710T)
mouse monoclonal anti-PSD95 (1:500; Thermo Fisher Scientific, #MA1-046)
chicken monoclonal anti-Tmem119 (1:500; Synaptic Systems, #400009)
rabbit polyclonal anti-Ifitm3 (1:100; Proteintech, #11714-1-AP)
rabbit polyclonal anti-Claudin5 (1:100; Thermo Fisher Scientific, #34-1600)
guinea pig monoclonal anti-GAD67 (1:100; Synaptic Systems, #198308)
rat monoclonal anti-Vcam1 (1:100; BD Biosciences, #550547)
rabbit polyclonal anti-VGLUT1 (1:100; Synaptic Systems, #135303)
rabbit polyclonal anti-VGLUT2 (1:100; Synaptic Systems, #135403)
Alexa Fluor 488-conjugated rat monoclonal anti-CD206 (1:100; Biolegend, #141709)
Alexa Fluor 647-conjugated rat monoclonal anti-CD169 (1:100; Biolegend, #142407)
Goat polyclonal anti-Axl (1:100; R and D, #AF854)
APC-conjugated rat monoclonal anti-Ly6C (1:50; Biolegend, #128015)

In vivo blocking
anti-CSF1R (CD115) (3.0 μg/μl; Bio X Cell, #BE0213)
rat IgG2a isotype control (3.0 μg/μl; Bio X Cell, #BE0089)
anti-CD9 (1.0 μg/μl; BD Biosciences, #553758)
normal rat IgG control (1.0 μg/μl; BD Biosciences, #553926)
anti-FcγRIII (0.5 μg/μl; R&D Systems, #MAB19601)
normal rat IgG control (0.5 μg/μl; R&D Systems, #MAB006)

Flow-cytometry
CD16/32 (1:100; BioLegend, #101320)
BV785-conjugated anti-mouse CD11b (1:100; BioLegend, #101243)
APC/Cyanine7-conjugated anti-mouse CD45 (1:100; BioLegend, #157204)
APC-conjugated anti-mouse Ly6C (1:100; BioLegend, #128016)
BV421-conjugated anti-mouse Tmem119 (1:300; Invitrogen, #404-6119-82)
PerCP/Cyanine5.5-conjugated anti-mouse CD9 (1:100; BioLegend, #124818,)
PE-conjugated anti-mouse Osteopontin/Spp1 (1:100; R&D Systems, #IC808P)
CoraLite594-conjugated anti-mouse IFITM3 (1:100; Proteintech, #CL594-11714)
PE/Cy7-conjugated anti-mouse cleaved caspase-3 (1:100; Cell Signaling Technology, #
64772)
Flow-cytometry (sorting prior to scRNA-seq)
FITC-conjugated anti-mouse CD11b (1:100; BioLegend, #101206)
APC-conjugated anti-mouse CD45 (1:500; BioLegend, #103112)

Validation

All antibodies are from commercially available sources and have been validated from the manufacturer with supporting information

on the manufacture's websites. We used them with the concentrations and conditions described there. For antibodies used in immunohistochemistry and flow cytometry, validation experiments were performed in our laboratory by verifying that staining patterns were in agreement with expected patterns. For antibodies used in Western blot, the expected band sizes were verified.

Animals and other research organisms

Policy information about studies involving animals; ARRIVE guidelines recommended for reporting animal research, and Sex and Gender in Research

Laboratory animals	Species: Mouse<br>Strain: C57BL/6 wild-type, Tmem119 CreERT2hM3Dq-mCitrineLSL/LSL mice, 5xFAD mice, and Tmem119 CreERT2 Cd9fl/fl mice<br>Age: 12 weeks old<br>Sex: male and female
Wild animals	N/A
Reporting on sex	Female mice were used for most experiments unless otherwise stated; selected validation experiments were performed in male mice, as described in the manuscript and figure legends
Field-collected samples	N/A
Ethics oversight	All the described procedures were approved the Institutional Animal Care and Use Commmitee at the University of Alabama at Birmingham.

Note that full information on the approval of the study protocol must also be provided in the manuscript.

Plants

Seed stocks	N/A
Novel plant genotypes	N/A
Authentication	N/A

Flow Cytometry

Plots

Confirm that:

- [x] The axis labels state the marker and fluorochrome used (e.g. CD4-FITC).
- [x] The axis scales are clearly visible. Include numbers along axes only for bottom left plot of group (a 'group' is an analysis of identical markers).
- [ ] All plots are contour plots with outliers or pseudocolor plots.
- [x] A numerical value for number of cells or percentage (with statistics) is provided.

Methodology

Sample preparation	Sample preparation procedures are described in the method section.
Instrument	BD LSRFortessa and FACSAria II
Software	Accuri C6 software, CellQuest, FACSDiva (BD Biosciences)<br>FlowJo
Cell population abundance	Cells were sorted using FACSAria II.
Gating strategy	Myeloid cell populations were gated on FSC/SSC plots and doublets were removed on FSC-A/FSC-H plot. Additional information is provided in the manuscript.

- [x] Tick this box to confirm that a figure exemplifying the gating strategy is provided in the Supplementary Information.
