# Supplementary material for: FcγR- and CD9-dependent synapse-engulfing microglia in the thalamus drive cognitive impairment following cortical brain damage in mice
Source: Nat Commun. 2026 Jul 9;17:8514. doi: 10.1038/s41467-026-74904-1 (PMC13484472; doi:10.1038/s41467-026-74904-1)
Supplement: Supplementary file 3 [file 41467_2026_74904_MOESM4_ESM.pdf]

Peer Review File

FcγR- and CD9-dependent synapse-engulfing microglia in the thalamus drive cognitive impairment following cortical brain damage in mice

Corresponding Author: Dr Shin-ichi Kano

This manuscript has been previously reviewed at another journal. This document only contains information relating to versions considered at Nature Communications.

This file contains all reviewer reports in order by version, followed by all author rebuttals in order by version.

Version 0:

Reviewer comments:

Reviewer #3

(Remarks to the Author)
The authors have thoroughly addressed my concerns. The new microglia-specific genetic tools, Axl/PS mechanistic data, snRNA-seq characterization of neuronal vulnerability, extended timepoints, and additional behavioral assays substantially strengthen the manuscript.

I encourage the authors to expand their Discussion on what is driving thalamic BBB selective impairments in this model (e.g., what is driving increased VCAM1, IgG extravasation).

I recommend acceptance.

Reviewer #4

(Remarks to the Author)
Matoba et al. investigate the mechanisms driving secondary cognitive impairment following primary cortical injury. They identify a specific neuroimmune axis in the thalamus responsible for chronic recognition memory deficits.

Key findings include:
- Cortical injury induces delayed thalamic microgliosis that correlates with novel object recognition (NOR) deficits.
- scRNA-seq identifies a unique, phagocytic, CD9hi microglial state (MG4) enriched in the thalamus.
- CD9hi microglia pathologically engulf glutamatergic synapses (VGLUT1/2-PSD95), causing excitatory synapse loss and neuronal hypoactivity.
- Blood-brain barrier (BBB) disruption permits IgG extravasation, which signals via microglial FcRIII to induce the CD9^hi state.
- Pharmacological or genetic targeting of CD9 or FcRIII prevents thalamic synapse loss and rescues memory.

Novelty and Significance
While secondary neurodegeneration and microglial activation in remote regions post-injury are well documented, this study defines a specific, targetable mechanism linking vascular permeability to synaptic loss. Identifying a CD9hi microglial subpopulation distinct from TREM2+ disease-associated microglia, and linking physical IgG extravasation to an FcRIII-mediated upregulation of CD9, represents an undescribed pathogenic axis in neurotrauma.

Evaluation of Revisions
The authors provided substantial new experimental data and methodological clarifications that directly address the initial critiques.
1. To address microglial specificity, new data from Tmem119-CreERT2 Cd9-floxed mice confirms that microglial CD9 drives synapse loss and cognitive deficits. Optimized DREADD experiments and flow cytometry effectively rule out off-target effects

on peripheral macrophages.
2. Regarding the direct role of CD9, EdU labeling demonstrates that CD9 blockade halts differentiation to the MG4 state without altering cell survival. Mechanistic data now links CD9hi microglia to Axl-mediated phagocytosis of phosphatidylserine-exposing synapses.
3. To validate synapse engulfment, improved Imaris 3D reconstructions verify PSD95 localization within CD68+ lysosomes. New snRNA-seq data confirms selective stress and VGLUT2 terminal loss in excitatory thalamic neurons.
4. To address BBB dysfunction, new sham controls confirm VCAM1 upregulation in thalamic endothelium. The addition of 5xFAD mice broadens the relevance of the IgG- CD9hi axis beyond physical trauma to general chronic neuroinflammation.
5. For methodological rigor, the authors included raw behavioral data and flow cytometry validation for the scRNA-seq clusters.

Recommendation: The rebuttal provides rigorous transgenic, transcriptomic, and behavioral data that resolve the reviewers' technical and conceptual concerns. The manuscript is significantly strengthened, and I recommend it for acceptance.

Open Access This Peer Review File is licensed under a Creative Commons Attribution 4.0 International License, which permits use, sharing, adaptation, distribution and reproduction in any medium or format, as long as you give appropriate credit to the original author(s) and the source, provide a link to the Creative Commons license, and indicate if changes were made.
In cases where reviewers are anonymous, credit should be given to 'Anonymous Referee' and the source.
The images or other third party material in this Peer Review File are included in the article's Creative Commons license, unless indicated otherwise in a credit line to the material. If material is not included in the article's Creative Commons license and your intended use is not permitted by statutory regulation or exceeds the permitted use, you will need to obtain permission directly from the copyright holder.
To view a copy of this license, visit https://creativecommons.org/licenses/by/4.0/

Reviewer #3 (Remarks to the Author):

The authors have thoroughly addressed my concerns. The new microglia-specific genetic tools, Axl/PS mechanistic data, snRNA-seq characterization of neuronal vulnerability, extended timepoints, and additional behavioral assays substantially strengthen the manuscript.
-- We appreciate this reviewer's suggestions and are glad to improve our manuscript.

I encourage the authors to expand their Discussion on what is driving thalamic BBB selective impairments in this model (e.g., what is driving increased VCAM1, IgG extravasation).
-- We have added further discussion on the potential mechanisms driving thalamic susceptibility to BBB disruptions.

I recommend acceptance.
-- We appreciate this reviewer's recommendation.

Reviewer #4 (Remarks to the Author):

Matoba et al. investigate the mechanisms driving secondary cognitive impairment following primary cortical injury. They identify a specific neuroimmune axis in the thalamus responsible for chronic recognition memory deficits.

Key findings include:
- Cortical injury induces delayed thalamic microgliosis that correlates with novel object recognition (NOR) deficits.
- scRNA-seq identifies a unique, phagocytic, CD9hi microglial state (MG4) enriched in the thalamus.
- CD9hi microglia pathologically engulf glutamatergic synapses (VGLUT1/2-PSD95), causing excitatory synapse loss and neuronal hypoactivity.
- Blood-brain barrier (BBB) disruption permits IgG extravasation, which signals via microglial FcRIII to induce the CD9^hi state.
- Pharmacological or genetic targeting of CD9 or FcRIII prevents thalamic synapse loss and rescues memory.

Novelty and Significance
While secondary neurodegeneration and microglial activation in remote regions post-injury are well documented, this study defines a specific, targetable mechanism linking vascular permeability to synaptic loss. Identifying a CD9hi microglial subpopulation distinct from TREM2+ disease-associated microglia, and linking physical IgG extravasation to an FcRIII-mediated upregulation of CD9, represents an undescribed pathogenic axis in neurotrauma.

Evaluation of Revisions
The authors provided substantial new experimental data and methodological clarifications that directly address the initial critiques.
1. To address microglial specificity, new data from Tmem119-CreERT2 Cd9-floxed mice confirms that microglial CD9 drives synapse loss and cognitive deficits. Optimized DREADD experiments and flow cytometry effectively rule out off-target effects on peripheral macrophages.
2. Regarding the direct role of CD9, EdU labeling demonstrates that CD9 blockade halts differentiation to the MG4 state without altering cell survival. Mechanistic data now links CD9hi microglia to Axl-mediated phagocytosis of phosphatidylserine-exposing synapses.

3. To validate synapse engulfment, improved Imaris 3D reconstructions verify PSD95 localization within CD68+ lysosomes. New snRNA-seq data confirms selective stress and VGLUT2 terminal loss in excitatory thalamic neurons.
4. To address BBB dysfunction, new sham controls confirm VCAM1 upregulation in thalamic endothelium. The addition of 5xFAD mice broadens the relevance of the IgG- CD9hi axis beyond physical trauma to general chronic neuroinflammation.
5. For methodological rigor, the authors included raw behavioral data and flow cytometry validation for the scRNA-seq clusters.

Recommendation: The rebuttal provides rigorous transgenic, transcriptomic, and behavioral data that resolve the reviewers' technical and conceptual concerns. The manuscript is significantly strengthened, and I recommend it for acceptance.

-- We appreciate this reviewer for making extensive evaluation of our revised manuscript.
